# Juvenile peripheral LPS exposure overrides female resilience to prenatal VPA effects on adult sociability in mice

**DOI:** 10.1038/s41598-024-62217-6

**Published:** 2024-05-19

**Authors:** Araceli Seiffe, Nadia Kazlauskas, Marcos Campolongo, Amaicha Mara Depino

**Affiliations:** 1 Facultad de Ciencias Exactas y Naturales, Departamento de Fisiología, Biología Molecular y Celular, Universidad de Buenos Aires, C1428EHA Buenos Aires, Argentina; 2 Facultad de Ciencias Exactas y Naturales, Departamento de Biodiversidad y Biología Experimental, Universidad de Buenos Aires, C1428EHA Buenos Aires, Argentina; 3 Instituto de Fisiología, Biología Molecular y Neurociencias (IFIBYNE), CONICET-UBA, Int. Guiraldes 2160, Ciudad Universitaria, Pabellón 2, 2do piso, C1428EHA Buenos Aires, Argentina

**Keywords:** Inflammation, Autism spectrum disorder, Social behaviour, Autism spectrum disorders

## Abstract

Autism spectrum disorder (ASD) exhibits a gender bias, with boys more frequently affected than girls. Similarly, in mouse models induced by prenatal exposure to valproic acid (VPA), males typically display reduced sociability, while females are less affected. Although both males and females exhibit VPA effects on neuroinflammatory parameters, these effects are sex-specific. Notably, females exposed to VPA show increased microglia and astrocyte density during the juvenile period. We hypothesized that these distinct neuroinflammatory patterns contribute to the resilience of females to VPA. To investigate this hypothesis, we treated juvenile animals with intraperitoneal bacterial lipopolysaccharides (LPS), a treatment known to elicit brain neuroinflammation. We thus evaluated the impact of juvenile LPS-induced inflammation on adult sociability and neuroinflammation in female mice prenatally exposed to VPA. Our results demonstrate that VPA-LPS females exhibit social deficits in adulthood, overriding the resilience observed in VPA-saline littermates. Repetitive behavior and anxiety levels were not affected by either treatment. We also evaluated whether the effect on sociability was accompanied by heightened neuroinflammation in the cerebellum and hippocampus. Surprisingly, we observed reduced astrocyte and microglia density in the cerebellum of VPA-LPS animals. These findings shed light on the complex interactions between prenatal insults, juvenile inflammatory stimuli, and sex-specific vulnerability in ASD-related social deficits, providing insights into potential therapeutic interventions for ASD.

## Introduction

Autism spectrum disorder (ASD) is a neurodevelopmental psychiatric condition characterized by deficits in sociability and communication, as well as repetitive and stereotyped behaviors^1^. One notable epidemiological factor is the significantly higher prevalence of ASD in males compared to females, with males being four times more likely to develop ASD^2^. While this discrepancy may in part stem from a bias in diagnosis favoring males^3^, evidence suggests the involvement of sex-specific biology in the development of ASD^4^.

Various biological mechanisms have been proposed to account for the increased susceptibility of boys to ASD. While ASD risk genes do not show differential expression between sexes, other genes exhibiting altered expression patterns in ASD brains display sex-specific differences^5^. Remarkably, markers for astrocytes and microglia were found to be more abundant in the male cortex, both prenatally and in adulthood. Alternatively, biological mechanisms of female resilience have been suggested. Among these, the “female protective model” posits that females require a greater number of contributing factors during development to exhibit ASD traits compared to males^4^.

While the investigation into sex differences in ASD has only recently gained attention, many mouse models of ASD demonstrate variations in phenotypes based on sex^6^. Notably, we have shown that female mice prenatally exposed to valproic acid (VPA) do not exhibit the reduced sociability observed in their male littermates^7^. However, female mice exposed to VPA in utero do display signs of neuroinflammation in the cerebellum and hippocampus during both the juvenile period^8^ and adulthood^7^, which differ from the astrocyte and microglia alterations observed in males. Specifically, we observed increased astrocyte density in adult VPA-exposed females in the cerebellum, as well as in the hippocampus at postnatal day (PD) 21 and 28. Meanwhile, microglia cell density was higher in VPA-exposed females in the adult cerebellum, and also in the hippocampus between PD21 and PD35. In addition, at PD35, VPA-exposed females exhibited lower densities of both astrocytes and microglial cells in the cerebellum. The cerebellum and hippocampus are particularly relevant to ASD, as individuals with ASD show alterations in anatomy and connectivity^9,10^, and signs of neuroinflammation^11^ in these structures.

Evidence suggests that environmental factors play a significant role in the onset of ASD. Perinatal inflammation resulting from infection, exposure to environmental allergens or pollutants, or autoimmune disease increases the risk of developing ASD^12–15^. Moreover, individuals diagnosed with ASD show signs of neuroinflammation^11,16,17^. Animal models recapitulate this epidemiological and clinical evidence, as maternal immune activation serves as an established model of ASD in rodents^18–21^ and non-immune murine models of ASD also exhibit alterations in neuroinflammation^8,22,23^. Furthermore, microglia play a role in shaping the developing brain, which can impact sociability, a core behavioral symptom of ASD^24,25^.

In this study, our aim was to assess whether the specific alterations observed in astrocytes and microglia during the juvenile period (PD21-PD35) contribute to the resilience of female mice to ASD-related behavior in adulthood. To achieve this, we administered a peripheral inflammatory stimulus (bacterial lipopolysaccharides, LPS) intraperitoneally every other day between PD22 and PD34, and then evaluated sociability and repetitive behaviors in adulthood. Additionally, we examined the neuroinflammatory state in the cerebellum and hippocampus of the four experimental groups, not only in adulthood, but also immediately after the first LPS injection (at PD22) and two days after the final LPS injection (at PD36).

## Results

### Juvenile LPS injection elicits peripheral and central inflammatory responses, but chronic exposure to LPS does not result in chronic sickness behavior

**Figure 1:**
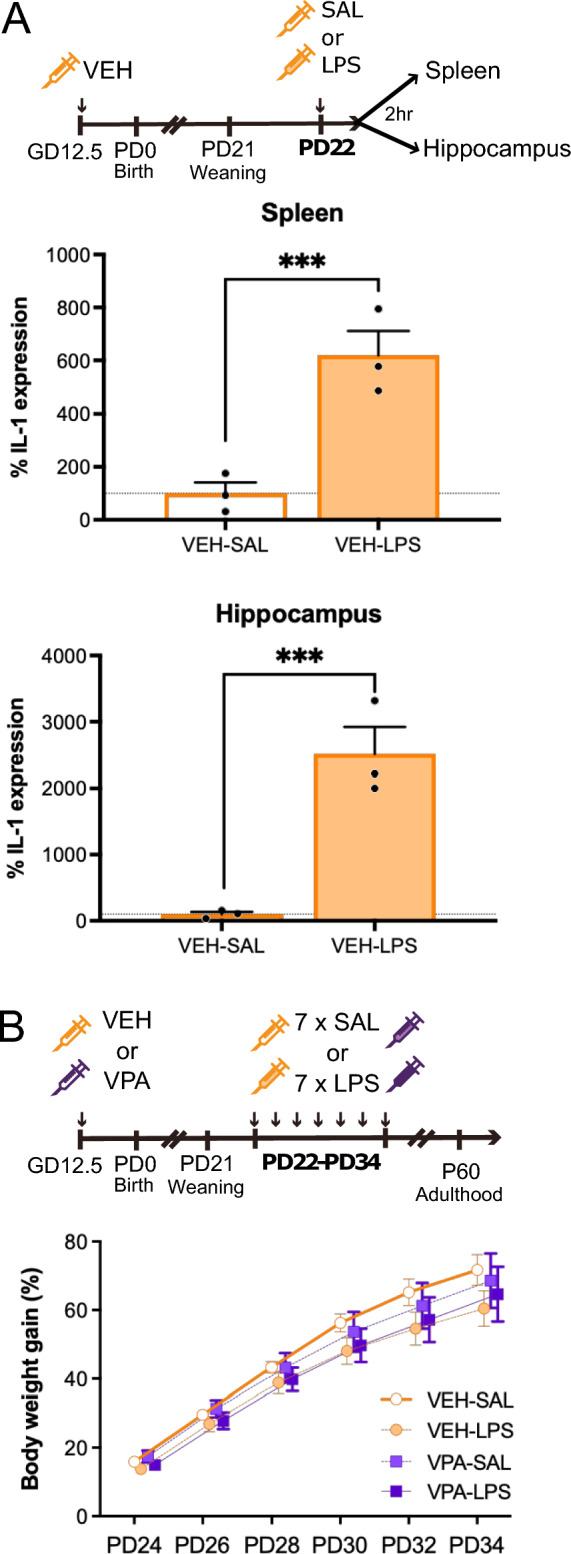
Intraperitoneal LPS injection induces an inflammatory response in young animals, and the every-other-day protocol of chronic inflammation allows animals to gain weight normally. (**A**) Animals prenatally exposed to the vehicle exhibit an increase in IL-1 expression 2 h after ip LPS injection, both in the spleen and in the hippocampus. ANOVA, Treatment effect: ***p < 0.001. n = 3 animals/group. (**B**) Throughout the juvenile chronic LPS injections, all animals exhibited similar weight gain patterns. n = 12–13 animals/group.

To assess the impact of LPS on peripheral and central inflammatory responses, we examined the expression of the proinflammatory cytokine interleukin-1beta (IL-1β) in the spleen and hippocampus two hours after intraperitoneal (ip) injection of LPS, as these areas are key components of the inflammatory response. Our results revealed a significant upregulation of IL-1β expression following LPS treatment, showing a six-fold increase in the spleen (χ^2^ = 26.975, Df = 1, p < 0.0001) and a twenty-five-fold increase in the hippocampus (χ^2^ = 34.838, Df = 1, p < 0.0001) (Fig. 1A). Worth to mention, in our prior investigation, we established that a single ip injection of 25 μg/kg LPS induced comparable plasma corticosterone levels in female offspring from dams treated with either vehicle (Prenatal VEH treatment) or valproic acid (VPA; Prenatal VPA treatment) at PD21, PD28 and PD35^8^. This finding suggests that both VEH and VPA animals may exhibit similar inflammatory responses induced by LPS across these developmental stages, although additional analysis is required.

To induce neuroinflammation throughout the juvenile period, coinciding with the observed alterations in neuroinflammation among females prenatally exposed to VPA^8^, we implemented a chronic LPS treatment. Specifically, we administered LPS between PD22 and PD34 at 48-h intervals, allowing animals to recover from each LPS injection and preventing chronic sickness behavior that might otherwise influence other parameters such as body weight gain. Indeed, using this protocol, all experimental groups exhibited similar patterns of body weight gain (ANOVA, Prenatal treatment x Juvenile treatment x Age interaction: χ^2^ = 3.996, Df = 5, p = 0.5500) (Fig. 1B). It is worth mentioning that when we analyzed the absolute body weight, we observed that VPA-treated mice were lighter (weight at PD34, prenatal treatment effect: χ^2^ = 4.1671, Df = 1, p = 0.0412), consistent with previous findings reported by our group^8^.

### Juvenile chronic LPS exposure impedes normal social behavior in adult VPA females

**Figure 2:**
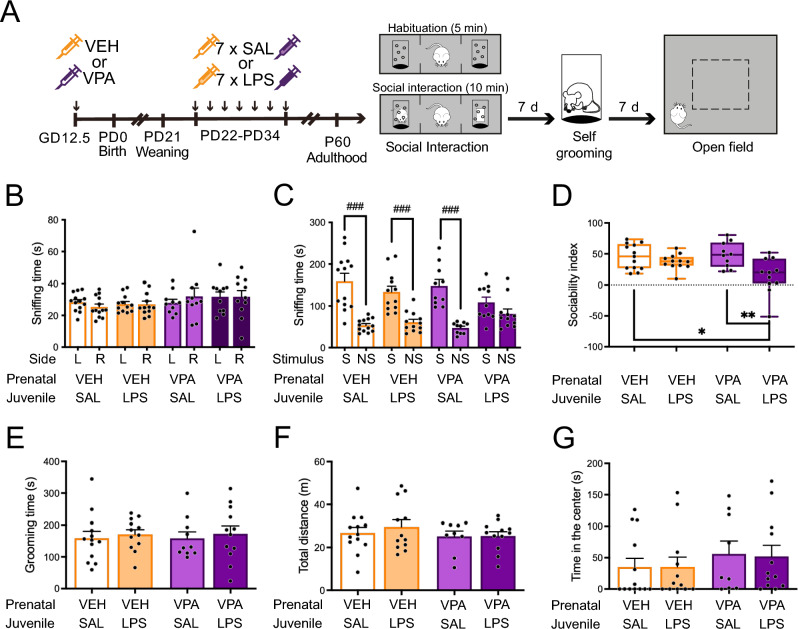
Juvenile LPS-exposure results in reduced sociability but normal repetitive behavior, exploration, and anxiety. (**A**) Animals were prenatally exposed to VPA or vehicle (VEH), and then injected every other day with LPS or saline (SAL) between PD22 and PD34. Behavior was evaluated in adulthood (> PD60). Animals were tested in the social interaction test, self-grooming test, and open field test, with one-week inter-test intervals. (**B**–**D**) Social interaction: (**B**) Time spent sniffing left (L) and right (R) cylinders during a 5-min habituation stage; (**C**) Time spent sniffing the cylinder containing the social stimulus (S) or the novel object (nonsocial, NS) during the 10-min test. ANOVA, Stimulus effect: ^###^p < 0.001. (**D**) Sociability index was calculated as (S-NS)/(S + NS). ANOVA followed by Tukey’s posthoc analysis: *p < 0.05, **p < 0.01. (**E**) Time spent grooming in the 10-min self-grooming test. (**F**,**G**) Open field: (**F**) Total distance walked during a 10-min open field exposure; (**G**) Time spent in the center of the open field. Individual data are depicted by black dots, and group information is represented by the mean ± s.e.m. (**B**), (**C**) and (**E**–**G**)) or a boxplot (**D**). n = 10–13 animals/group.

Animals prenatally exposed to VPA or VEH and treated with either LPS or SAL between PD22 and PD36 were assessed as adults in three behavioral tests: the three-chambered social interaction test, the self-grooming test, and the open field test (Fig. 2A). We observed that adult VPA-LPS females exhibited no preference for social stimuli, spending an equivalent amount of time sniffing the cylinder with a novel mouse and the cylinder containing a novel object (ANOVA: χ^2^ = 2.6901, Df = 1, p = 0.101; Fig. 2C). Conversely, all other groups displayed the expected preference for the social stimulus. Analysis of the sociability index revealed an interaction between prenatal and juvenile treatments (χ^2^ = 4.3017, Df = 1, p = 0.0381), with VPA-LPS mice showing reduced sociability compared to VEH-SAL and VPA-SAL animals (Fig. 2D). This effect was not attributable to differences in exploration among groups, as all animals explored both cylinders similarly during the habituation stage of the social interaction test (Fig. 2B) and exhibited similar exploration patterns in the open field (Fig. 2F). Moreover, we found no differences in anxiety-related behavior, with VPA-LPS animals spending a comparable amount of time in the center of the open field compared to all other experimental groups (Fig. 2G).

We also evaluated repetitive behavior, a trait altered in male mice prenatally exposed to VPA, through the self-grooming test^26^. We observed that VPA-SAL females did not exhibit increased self-grooming time compared to controls. Furthermore, juvenile LPS exposure did not influence this behavior (Fig. 2E).

### The long-lasting cerebellar neuroinflammatory effect of prenatal VPA exposure is reversed by juvenile exposure to LPS

Previously, we observed signs of neuroinflammation in the cerebellum of male mice prenatally exposed to VPA, which exhibited decreased sociability^22^. Furthermore, the same study demonstrated that eliciting inflammation in the lobule 7 of the cerebellum resulted in adult mice displaying reduced social interaction. To investigate whether cerebellar inflammation could contribute to the observed reduction in sociability in VPA-LPS females, we analyzed the GFAP-positive area and Iba1-positive cells in the molecular and granular cell layers of the lobule 7 of the cerebellum in adult animals from the four experimental groups.

**Figure 3:**
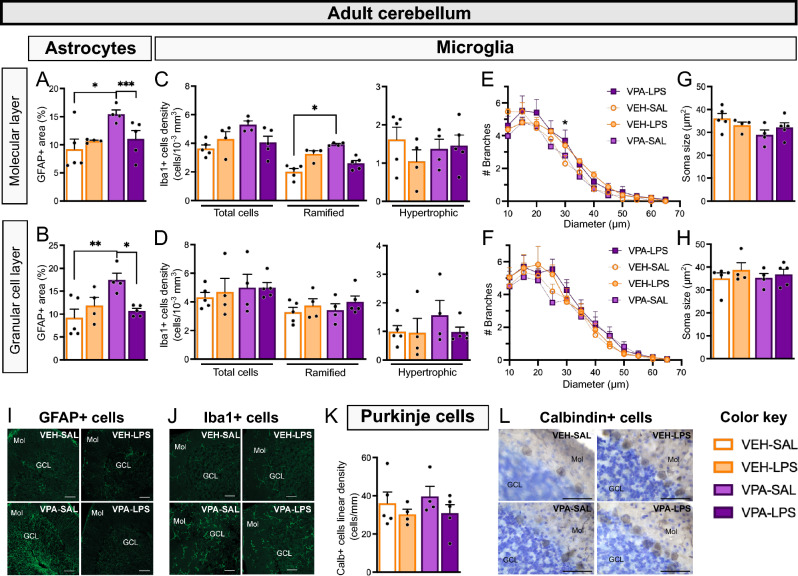
Juvenile LPS exposure reverses VPA effects on cerebellar astrocytes and microglia. GFAP-positive area was determined in the molecular layer (**A**) and granular cell layer (**B**) of the lobule 7 of the cerebellum. Microglial (Iba1-positive) cell density was quantified in the molecular layer (**C**) and granular cell layer (**D**) of the lobule 7 of the cerebellum. In each region, total cells, ramified cells, and hypertrophic cells are reported. (**E**,**F**) Sholl analysis of Iba1-positive cells is reported for both regions. (**G**,**H**) Soma size was estimated for Iba1-positive cells. (**I**) Representative images of anti-GFAP immunofluorescence. Scale bar, 50 μm. (**J**) Representative images of anti-Iba1 immunofluorescence. Scale bar, 50 μm. (**K**) Linear density of calbindin-positive Purkinje cells in the lobule 7 of the cerebellum is shown. (**L**) Representative images of anti-calbindin immunohistochemistry. Scale bar, 50 μm. n = 4–5 animals/group. ANOVA followed by Tukey’s posthoc analysis: *p < 0.05, **p < 0.01, ***p < 0.001.

We found that VPA-SAL animals exhibited a larger GFAP-positive area in both the molecular layer (Fig. 3A and I; prenatal x juvenile treatments interaction: χ^2^ = 1333.47, Df = 1, p < 0.0001) and granular cell layer of the cerebellum (Fig. 3B and I; prenatal x juvenile treatments interaction: χ^2^ = 8.6454, Df = 1, p = 0.0033) compared to VEH-SAL and VPA-LPS animals. These findings replicate previous reports of increased astrocytes in the cerebellum of VPA females^7^, and show that juvenile exposure to LPS reduces this parameter. In the hippocampus, only a main effect of prenatal treatment was observed in the pyramidal cell layer of the CA1 (χ^2^ = 7.0906, Df = 1, p = 0.0077), while other analyzed areas of the CA1 showed similar GFAP-positive cell densities in all experimental groups (Supplementary Fig. 1A–C). In the dentate gyrus (DG), a significant interaction between prenatal and juvenile treatments in the density of GFAP-positive cells in the molecular layer was observed (χ^2^ = 10.2964, Df = 1, p = 0.0013), although no differences between experimental groups were detected with *posthoc* analysis (Supplementary Fig. 2A). The granular cell layer and hilus exhibited similar GFAP-positive cell densities across all experimental groups (Supplementary Fig. 2B,C), demonstrating the specificity of the effect on cerebellar astrocytes.

Regarding cerebellar microglia in the molecular layer (Fig. 3C and J), we observed an interaction between prenatal and juvenile treatments in the density of Iba1-positive cells (χ^2^ = 6.4598, Df = 1, p = 0.0110) and the density of ramified microglial cells (χ^2^ = 44.9501, Df = 1, p < 0.0001). VPA-SAL animals displayed higher ramified microglial density than control VEH-SAL animals, while VPA-LPS cerebella showed intermediate values. No differences between groups were observed in microglial cells in the granular cell layer (Fig. 3D and J). Additionally, few hypertrophic microglial cells were observed in both regions, with no differences between groups detected. Sholl analysis revealed that in the molecular layer, microglia of LPS-treated animals exhibited more branches (Juvenile treatment x diameter interaction: χ^2^ = 22.9295, Df = 11, p = 0.0181; Fig. 3E) and had a smaller cell area (Juvenile treatment effect: χ^2^ = 17.5323, Df = 1, p < 0.0001; Supplementary Fig. 3A) compared to SAL-treated animals, while no differences were observed in the granular cell layer (Fig. 3F). No differences were detected in the size of microglial soma (Fig. 3G and H), nor in sphericity or maximal radius (Supplementary Fig. 3). These results indicate a pattern of microglial cells similar to that observed for astrocytes, supporting the observation of a long-lasting neuroinflammatory effect of VPA that is reversed when animals are exposed to LPS during the juvenile period. Again, this effect is specific to the cerebellum, as no differences in microglial cell density were observed in the hippocampus, including both the CA1 (Supplementary Fig. 1D–F) and the DG (Supplementary Fig. 2D–F).

### Neither VPA prenatal exposure nor LPS juvenile treatment alter the linear density of Purkinje cells in the lobule 7 of the cerebellum

Maternal immune activation has been associated with offspring exhibiting diminished sociability^20^ and a reduction in the number of Purkinje cells in the lobule 7 (L7) of the cerebellum^27^. A loss of Purkinje cells has also been reported in the brains of individuals with ASD, correlating with clinical parameters^28^. Previously, we demonstrated that VPA exposure did not affect Purkinje cells in either females^8^ or males^7^. In this study, we measured the linear density of calbindin-positive Purkinje cells to assess the impact of juvenile LPS exposure on this neuronal population. Our findings indicate no significant effect of prenatal VPA exposure or juvenile LPS treatment on the density of these cells (Fig. 3K and L).

### Mild microglial activation is observed in the cerebellum at PD22, 2 h after an LPS injection

**Figure 4:**
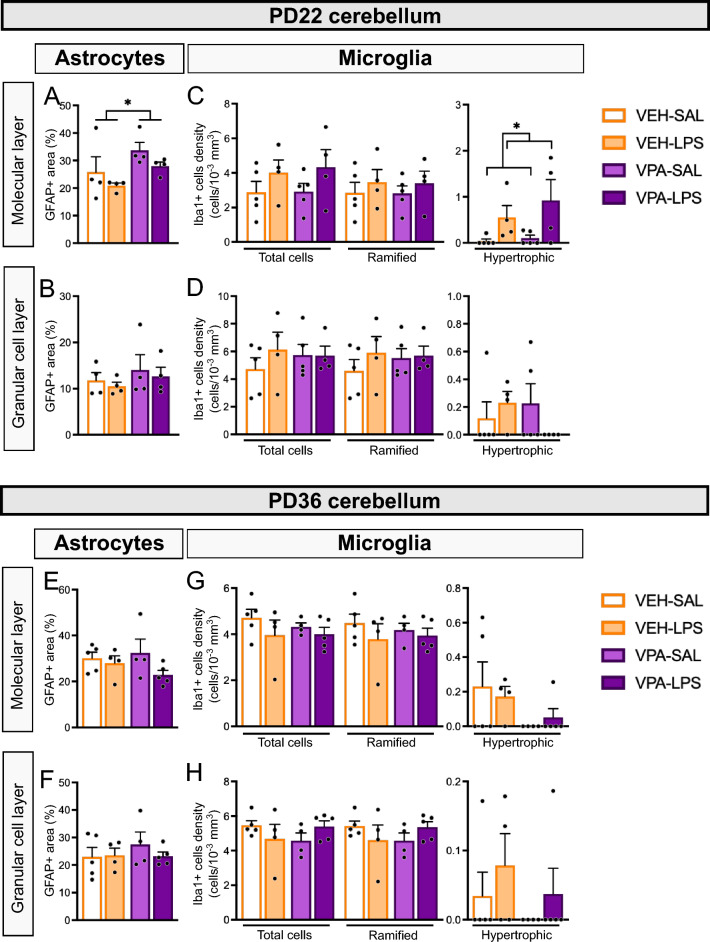
Acute juvenile LPS effects on cerebellar lobule 7 astrocytes and microglia. (**A**–**D**) Two hours after SAL or LPS injection at PD22, GFAP and Iba1-positive cells were analyzed in the lobule 7 of the cerebellum. GFAP-positive area was determined in the molecular layer (**A**) and granular cell layer (**B**) of the lobule 7 of the cerebellum. Microglial (Iba1-positive) cell density was quantified in the molecular layer (**C**) and granular cell layer (**D**) of the lobule 7 of the cerebellum. Total cells, ramified cells, and hypertrophic cells are reported for each region. (**E**–**H**) At PD36 -two days after the last SAL or LPS injection-, GFAP and Iba1-positive cells were analyzed in the lobule 7 of the cerebellum. GFAP-positive area was determined in the molecular layer (**E**) and granular cell layer (**F**) of the lobule 7 of the cerebellum. Microglial (Iba1-positive) cell density was quantified in the molecular layer (**G**) and granular cell layer (**H**) of the lobule 7 of the cerebellum. Total cells, ramified cells, and hypertrophic cells are reported for each region. n = 4–5 animals/group. Two-way ANOVA main effects: *p < 0.05.

We assessed astrocytes in the cerebellum 2 h after the first SAL or LPS injection at PD22 by calculating the GFAP-positive area. In the molecular layer, we observed a significant effect of prenatal treatment, with VPA-exposed animals presenting a larger area [ANOVA prenatal effect: χ^2^ = 4.3990, Df = 1, p = 0.0360; Fig. 4A]. However, we did not observe differences between groups in the GFAP-positive area in the granular cell layer of the cerebellum at this age (Fig. 4B). In the hippocampus, a decrease in GFAP-positive cell density was observed in animals injected with LPS, particularly in the *stratum radiatum* of CA1 [ANOVA, PD22 treatment effect: χ^2^ = 12.6173, Df = 1, p < 0.001; Supplementary Fig. 4C], while an interaction between prenatal and PD22 treatments was observed in the molecular layer of the DG (χ^2^ = 4.2071, Df = 1, p = 0.0402; Supplementary Fig. 5A). No differences between groups were observed in other areas of CA1 (Supplementary Figs. 4A and B) or DG (Supplementary Fig. 5B and C).

We also evaluated the density of microglial cells at PD22. We found a significant effect of PD22 treatment on the density of hypertrophic Iba1-positive cells, with peripheral LPS eliciting a neuroinflammatory effect in this area (ANOVA PD22 effect: χ^2^ = 5.4489, Df = 1, p = 0.0196; Fig. 4C). However, no differences between groups were observed in the granular cell layer of the cerebellum (Fig. 4D). In the hippocampus, we observed a significant interaction between prenatal and PD22 treatments in the density of microglial cells in the pyramidal cell layer of CA1 (χ^2^ = 4.4096, Df = 1, p = 0.0357; Supplementary Fig. 4E) and in the molecular layer of the DG (χ^2^ = 13.1047, Df = 1, p =  < 0.001; Supplementary Fig. 5D), although no differences between groups were observed in Tukey *posthoc* comparisons. Additionally, there were no differences between groups in microglial cells in CA1 *stratum oriens* (Supplementary Fig. 4D) or the *stratum radiatum* (Supplementary Fig. 4F), nor in the DG granular cell layer (Supplementary Fig. 5E) or the hilus (Supplementary Fig. 5F).

These results indicate a similar neuroinflammatory response in VEH and VPA mice following a peripheral inflammatory stimulus at PD22.

### Similar neuroinflammatory parameters are observed at PD36 in VEH and VPA brains after 7 peripheral injections of LPS

We also analyzed the GFAP-positive area and Iba1-positive cell density in the lobule 7 of the cerebellum at PD36, two days after the last SAL or LPS injection, to evaluate the short-term effects of this chronic inflammatory protocol on astrocytes and microglia. However, we found no significant effects of the prenatal or juvenile treatments on astrocytes (Fig. 4E and F) or microglia (Figs. 4G and H) in the cerebellum. Similarly, in the hippocampus, no differences were observed between groups in the density of GFAP-positive cells in the CA1 (Supplementary Figs. 4G–I) or the DG (Supplementary Figs. 5G–I).

Moreover, in the pyramidal cell layer of the CA1, we observed an interaction between prenatal and juvenile treatments in total microglial cell density (χ^2^ = 6.879, Df = 1, p = 0.0087) and ramified microglial cell density (χ^2^ = 9.4742, Df = 1, p = 0.0021), with a higher density of cells in VEH-SAL animals compared to the VEH-LPS group (Supplementary Fig. 4K). We did not observe differences in the *stratum oriens* (Supplementary Fig. 4J) or the *stratum radiatum* (Supplementary Fig. 4L). In the DG, we observed an interaction between prenatal and juvenile treatments in the density of microglial cells in the granular cell layer (χ^2^ = 6.7469, Df = 1, p = 0.0094), and VPA-LPS animals showed more microglial cells than VPA-SAL animals in this area (Supplementary Fig. 5K). No differences were observed in the molecular layer (Supplementary Fig. 5J) or the *hilus* (Supplementary Fig. 5L).

These results show that neuroinflammatory parameters were mainly unaltered at the end of the chronic inflammatory protocol.

## Discussion

Our findings reveal that chronic inflammation during the juvenile period affects the development of sociability in female mice prenatally exposed to VPA, resulting in adult animals with reduced social interaction and a lack of social preference. Notably, this effect does not coincide with alterations in exploration, the expression of repetitive behavior, or anxiety. Additionally, we observed changes in the cerebellar neuroinflammatory state, indicating that the secondary exposure to LPS actually mitigates the proinflammatory effect of VPA in this region.

There are various murine models of ASD, many of which exhibit sex differences in their phenotypes^6^. Interestingly, akin to observations in humans, mouse models demonstrate that being male is a risk factor for developing altered sociability following environmental and genetic challenges. For instance, male mice subjected to LPS challenge at PD9 display reduced sociability later in life, while female mice show resilience to this behavioral effect of early-life immune activation^29^. Similarly, our previous findings indicated that male mice prenatally exposed to VPA exhibit reduced sociability, while female mice are spared from this behavioral effect^7^.

Recent evidence suggests that female animals, when subjected to a protocol of brain masculinization by postnatal exposure to estradiol, may become predisposed to the effects of LPS injection at PD9, consequently resulting in diminished sociability^30^. This has led to the proposition of a double-hit model for the developmental determination of this behavior in females. Our study results similarly support the concept that VPA may render animals susceptible to a second hit. In males, this secondary event may be the masculinization induced by postnatal gonadal steroids, which is absent in females^30,31^. Alternatively, we identified a later critical period, between PD21 and PD35, during which we previously observed that VPA-exposed males, when subjected to social enrichment^26^ or handling^32^, exhibit normal sociability in adulthood, thus mitigating the effects of VPA on this behavior. Interestingly, during this period, male and female mice are differentially exposed to gonadal steroids, as ovaries are already producing estrogens, while testicles are only marginally synthetizing testosterone^6^. Neonatally masculinized females do not develop functional ovaries^31^, altering the typical female pattern of gonadal hormones throughout life. Accordingly, an alternative interpretation of Bordt et al.^30^ results is that female gonadal hormones participate in the resilience to the effects of LPS at PD9 on sociability, and that this is impeded in masculinized animals. To evaluate this, it would be interesting to assess how removing gonadal hormones during the juvenile period from females injected with LPS at PD9 affects sociability.

During development, sexes not only differ in the abundance of gonadal steroids but also in various other parameters of the internal milieu^33^. Among these factors, neuroinflammatory molecules and cells have emerged as relevant^34^. Astrocytes and microglia play significant roles in the development and refinement of neuronal circuits^35,36^, and they exhibit sex differences during development^37,38^. Additionally, microglia are crucial for brain masculinization^39^, and modulation of neuroinflammation during the juvenile period can alter brain masculinization and affect social behavior^40^. We previously reported sex differences in the neuroinflammatory effects of VPA on the adult brain^7^, and observed signs of increased neuroinflammation in the brains of VPA-exposed females during the juvenile period^8^. We hypothesized that these primed astrocytes and microglia could be activated by a peripheral pro-inflammatory stimulus, as demonstrated in models of neurodegenerative disorders^41–43^. Consequently, these activated glial cells could affect neuronal development and lead to altered ASD-related behaviors. Indeed, we found reduced sociability in VPA-LPS females, while other behaviors remained unaffected. Surprisingly, we found no evidence of exacerbated neuroinflammation in the brains of VPA-LPS animals, neither after the initial LPS injection at PD22 nor upon completion of the chronic LPS treatment at PD36. Moreover, LPS-treated animals did not exhibit long-lasting astrogliosis or microgliosis. Instead, VPA-LPS animals showed reduced GFAP-positive area and fewer Iba1-positive cells in the cerebellum compared to VPA-VEH females. This suggests that the juvenile inflammatory stimulus decreases the neuroinflammation caused by prenatal VPA exposure.

Several explanations could account for this phenomenon. First, different brain regions can respond differently to inflammatory stimuli, as demonstrated in studies involving cytokine injection in the brain^44^ and peripheral LPS stimulation^22^. We focused on the cerebellum due to observed glial alterations in VPA-exposed males and our previous findings that eliciting inflammation in the lobule 7 resulted in reduced sociability^22^. However, it’s important to acknowledge that other brain regions, such as the prefrontal cortex and hypothalamus^45,46^, are also relevant to rodent social behavior and could be influenced by VPA and LPS exposure. Therefore, our study is constrained by its focus on examining only the cerebellum, the DG, and CA1, representing a limitation in the scope of our investigation. Secondly, studying the density and morphology of glial cells may preclude the identification of functional alterations in these cells that do not affect their number and geometry. Further characterization of gene expression and evaluation of cell function may reveal relevant differences in both astrocytes and microglia in animals exposed to VPA and LPS^47^. Lastly, we evaluated the effects of LPS and VPA at three time points—PD22, PD36, and adulthood. However, differential effects on neuroinflammation may occur at other time points, potentially leading to long-lasting alterations in neuronal circuits. We are convinced that the model presented in this study can be used to further identify relevant regions and critical ages crucial for determining sociability in females.

Our study was limited to analyzing female offspring, which represents a limitation. However, studying males would require a different experimental design as the deficit of social behavior in VPA-exposed animals can be rescued by the handling of animals required for the administration of LPS^32^. Using delivery systems such as osmotic minipumps^48^ may be needed.

In conclusion, we propose that social behavior is programmed perinatally but consolidated during the juvenile period. Genetic factors as well as prenatal factors such as VPA exposure or maternal immune activation might affect the development of the circuits responsible for social behavior, but their final effect would depend on the exposure of those circuits to further stimuli, such as gonadal steroids, inflammatory stimuli, or a social environment. The identification of these stimuli might be crucial to our understanding of ASD and the development of therapeutics. Viral and bacterial infection are not only extremely common during the first years of life but also inevitable. Understanding their role in eliciting ASD in vulnerable individuals may help develop treatments to reverse their effects. In this regard, the identification of critical periods and relevant molecular pathways is fundamental. We hope that our work contributes to such developments.

## Materials and methods

### Animals

Outbred CrlFcen:CF1 adult mice, both female and male, were obtained from the animal house at the Faculty of Exact and Natural Sciences, University of Buenos Aires (Buenos Aires, Argentina). We selected this outbred stock due to its superior breeding performance compared to inbred strains and its consistent postnatal behavior. Moreover, prior research has demonstrated that female CF1 mice prenatally exposed to VPA do not display the reduction in sociability observed in males^7^. All mice were housed in the animal facility in open-top cages, under a 12:12 light:dark cycle and maintained at a controlled temperature of 18–22 °C, with access to food and water ad libitum. For experimental design and preparation of the manuscript, we followed the recommendations in the ARRIVE guidelines. All experimental procedures involving animals adhered to the regulations for the use of laboratory animals of the National Institute of Health, Washington, DC, USA, and were approved by the institutional animal care and use committee of the Faculty of Exact and Natural Sciences, University of Buenos Aires (CICUAL Protocol Nr. 6/2012).

Male mice aged 8–10 weeks were paired with nulliparous female mice. The females were monitored daily to detect the presence of a vaginal plug, with the day of detection considered gestational day (GD) 0.5.

#### Prenatal treatment

On GD12.5, pregnant mice were subcutaneously injected with either 600 mg/kg of valproic acid sodium salt (VPA; Cat. Nr. P4543, Sigma, St. Louis, MO, USA) dissolved in saline solution or with vehicle, in a volume of 3 ml per kg of mouse weight. Therefore, two prenatal treatments were administered: VPA or vehicle (VEH). The day of parturition was recorded as postnatal day 0 (PD0), and the bedding in the cages was left unchanged during the first postnatal week to prevent disturbances to the nests. Four independent cohorts of animals were utilized for this study:

Cohort 1: Offspring from 3 VEH dams were employed to evaluate the acute response to LPS at PD22 (see Fig. 1A).

Cohort 2: Offspring from 18 dams (9 VEH, 9 VPA) were utilized for adult behavioral and histological analyses (see Figs. 1B, 2 and 3).

Cohort 3: Offspring from 13 dams (7 VEH, 5 VPA) were assessed at PD22 (see Fig. 4A–D).

Cohort 4: Offspring from 6 dams (4 VEH, 2 VPA) were used for PD36 analyses (see Fig. 4E–H).

We observed no differences between VEH and VPA litters in terms of gestational length, litter size, or male to female ratio (Supplementary Fig. 6). On PD21, litters were weaned into cages containing 4–5 animals of the same sex and treatment. Offspring within the same treatment group were mixed at weaning to mitigate the litter effect and same-cage influence. Only female offspring were examined in this study.

#### Juvenile treatment

Following weaning, animals in each cage were randomly assigned to receive either LPS or saline treatment. Juvenile LPS treatment involved seven intraperitoneal (ip) injections of 25 μg/kg LPS (*Escherichia coli* LPS, serotype 0111:B4, Cat. Nr. L2630, Sigma-Aldrich, St. Louis, USA) in sterile saline solution, administered every other day from PD22 to PD34, in a volume of 5 ml per kg of mouse weight. Control animals received seven injections of sterile saline solution (Juvenile SAL treatment). The chosen dose was based on its ability to induce a peripheral inflammatory response reaching the brain without causing lasting sickness behavior^22^. Injections were administered at the same time each day, between 10:00 am and 12:00 pm.

### Analysis of IL-1β expression

The expression of IL-1β was assessed using Real-time PCR, following previously established protocols^22^. In brief, animals from cohort 1 were euthanized at PD22, two hours intraperitoneal (ip) injection of either SAL or LPS, and their spleens and hippocampi were promptly dissected and frozen in liquid nitrogen. Total RNA extraction was performed using Trizol (TRI Reagent, Molecular Research Center Inc., OH, USA), followed by reverse transcription using an MMLV enzyme kit (Promega, WI, USA), as outlined in previous studies^22^. The levels of beta-actin and IL-1β were determined using previously published primers^49^, employing the SYBR-green I fluorescent method, and generating a reference curve for each molecule. The StepOne Real-Time PCR System and Software v2.3 (Applied Biosystems, CA, USA) were utilized for analysis. Only animals receiving vehicle (VEH) were included in the study, as we previously demonstrated that prenatal exposure to VPA does not alter the peripheral inflammatory response in young animals^8^. Three animals per group were analyzed.

### Behavioral testing

Adult mice (PD70) underwent sequential testing in the social interaction, self-grooming and open field tests, with one-week intervals between tests to minimize the interference between tests. A total of 13 VEH-SAL, 12 VEH-LPS, 10 VPA-SAL, and 12 VPA-LPS animals from cohort 2 were included in the study. Testing sessions were conducted during the light period (between 9:00 and 16:00 h). Prior to testing, mice were acclimated to the light levels in the testing room for 30 min. Following testing, each mouse was identified and placed in a holding cage until all animals within a cage had completed testing. The testing order for each cage was randomly determined. All apparatuses were cleaned with 20% ethanol between sessions to eliminate odors and waste. Manual scoring was conducted by an experimenter (N.K.), who was blinded to the treatment groups.

### Social interaction test

The social interaction test was conducted according to established protocols^7,26,50^. Briefly, animals were habituated for 5 min to a three-chamber cage (45 cm × 15 cm) containing a Plexiglas tube in each lateral chamber. Subsequently, a young (PD21-PD28) female CF1 mouse was introduced in one tube (social side), while a white object was placed in the other (nonsocial side). The tubes, with a diameter of 7.5 cm, featured several holes to facilitate visual, olfactory, and auditory investigation between the stimulus and test mice. Trials were recorded, and the time spent sniffing the tubes during the habituation session or the social stimulus/novel object during the testing session was manually quantified using a video-tracking system (ANY-maze, Stoelting, IL, USA). The sociability index was calculated as the time spent sniffing the social stimulus minus the time spent sniffing the nonsocial stimulus (novel object), divided by the total time of sniffing. One VPA-LPS animal was excluded from the analysis due to tracking failure. Dim lighting conditions (10 lx) were maintained during this test.

#### Self-grooming test

The self-grooming test was conducted as previously described^26,50^. Animals were habituated to being enclosed in a 5.5-cm diameter and 20-cm high Plexiglas tube during two 60-min sessions on consecutive days. The test was performed on the third day, after a 10-min habituation period, and lasted for 10 min. Dim lighting conditions (10 lx) were maintained during this test. Testing sessions were recorded, and the time spent grooming during the session was measured from the video.

#### Open field test

The open field test was performed as previously described^22,26^. Briefly, animals were exposed to a 30 cm × 45 cm × 45 cm black arena for 10 min under bright light conditions (100 lx). The arena was divided into two sections: the center, defined as the central 23 cm × 23 cm square, and the periphery. Total distance traveled and time spent in the center were measured using a video-tracking system (ANY-maze). One VPA-SAL animal was excluded from the analysis due to abnormal hyperactivity.

### Histological analyses

Four to five animals per experimental group were randomly selected from cohorts 2, 3, and 4 for the analysis of astrocyte and microglia cells, following previously established methods^7,8^. Briefly, animals were deeply anesthetized and transcardially perfused with 4% paraformaldehyde. The brains were then processed and sectioned using a cryostat. Sections with a thickness of 40 μm were subjected to immunofluorescence staining using the anti-GFAP antibody (DAKO, Glostrup, Denmark) to detect astrocytes, and the anti-Iba1 antibody (WAKO, Osaka, Japan) for microglia. Microglia cells were morphologically classified as ramified or hypertrophic, following previously described criteria^7,8^. Confocal microscopy images were obtained at 400 × magnification with 1 μm Z-stacking using either an Olympus FV300/BX61 or FV1000 microscope. Image analysis was performed using Fiji software^51^ to quantify cellular density or GFAP-positive area. Sholl analysis was conducted using the Fiji tool^52^.

### Statistics and data availability

Graphs were generated with Prism 8 (GraphPad Software, LLC). Statistical analyses were conducted using R software. In all cases, mixed-effects models were fitted to account for the litter effect. We used the lme function (nlme package) and assumed normality. Whenever normality assumptions were not met, we applied data transformations. If data still did not conform to a normal distribution, we employed the glmmTMB function (glmmTMB package) to model gamma distribution or zero-inflated data. The significance of fixed effects was assessed using the Anova function (car package) with Type III tests, followed by Tukey post hoc comparisons using the emmeans function (emmeans package). Normality and homoscedasticity assumptions were assessed graphically using QQ plots and Pearson’s residuals vs Predicted values plots, and statistically tested using the Shapiro–Wilk and Levene tests. Full statistical results are provided as a supplementary file (Complete_statistics_results file). The R markdown script containing the complete statistical analysis is provided as a supplementary file (Statistical_code file), along with the original data (Original_data file).

### Supplementary Information

Supplementary Figures.

Supplementary Information 1.

Supplementary Information 2.

Supplementary Information 3.

## Data Availability

All data generated or analyzed during this study are included in this published article (and its Supplementary Information files).
