# Supplementary material for: Juvenile peripheral LPS exposure overrides female resilience to prenatal VPA effects on adult sociability in mice
Source: Sci Rep. 2024 May 19;14:11435. doi: 10.1038/s41598-024-62217-6 (PMC11102908; doi:10.1038/s41598-024-62217-6)
Supplement: Supplementary file 1 — Supplementary Figures. [file 41598_2024_62217_MOESM1_ESM.docx]

**SUPPLEMENTARY INFORMATION**

**Juvenile peripheral LPS exposure overrides female resilience to prenatal VPA effects on adult sociability in mice**

Araceli Seiffe^1,2^, Nadia Kazlauskas^1,2^, Marcos Campolongo^1,2^, Amaicha Mara Depino^1,2,3^

^1^ Universidad de Buenos Aires, Facultad de Ciencias Exactas y Naturales, Departamento de Fisiología, Biología Molecular y Celular, C1428EHA, Buenos Aires, Argentina

^2^ CONICET-Universidad de Buenos Aires, Instituto de Fisiología, Biología Molecular y Neurociencias (IFIBYNE), Buenos Aires, Argentina.

^3^ Universidad de Buenos Aires, Facultad de Ciencias Exactas y Naturales, Departamento de Biodiversidad y Biología Experimental, C1428EHA, Buenos Aires, Argentina

Corresponding author: Amaicha Mara Depino, Ph.D., Instituto de Fisiología, Biología Molecular y Neurociencias (IFIBYNE), CONICET-UBA. Int. Guiraldes 2160, Ciudad Universitaria, Pabellón 2, 2do piso, C1428EHA, Buenos Aires, Argentina. Email: adepino@conicet.gov.ar. Tel: +5411-4576-3300, ext. 469. Fax: +5411-4576-3447.

**SUPPLEMENTARY FIGURES**

**Supplementary Figure 1. Effects of prenatal VPA and juvenile LPS exposure on astrocytes and microglia in the CA1 of the hippocampus**. The density of astrocytes (GFAP-positive cells) was determined in the *stratum oriens* (A), pyramidal cell layer (B), and *stratum radiatum* (C) of the CA1. Microglial (Iba1-positive) cell density was quantified in the *stratum oriens* (D), pyramidal cell layer (E), and *stratum radiatum* (F) of the CA1. Total cells, ramified cells, and hypertrophic cells are reported for each region. n = 4-5 animals/group. Two-way ANOVA main effect: ** p < 0.01.

**Supplementary Figure 2. Effects of prenatal VPA and juvenile LPS exposure on astrocytes and microglia in the DG of the hippocampus**. The density of astrocytes (GFAP-positive cells) was determined in the molecular layer (A), granular cell layer (B), and hilus (C) of the DG. Microglial (Iba1-positive) cell density was quantified in the molecular layer (D), granular cell layer (E), and hilus (F) of the DG. Total cells, ramified cells, and hypertrophic cells are reported for each region. n = 4-5 animals/group.

**Supplementary Figure 3. Effects of prenatal VPA and juvenile LPS exposure on microglia in lobule 7 of the cerebellum**. Cell area, sphericity, and maximal radio were determined for microglial cells (Iba1-positive) in the molecular layer (A, C and E) and granular cell layer (B, D and F) of lobule 7 of the cerebellum. ANOVA, Juvenile treatment effect: * p < 0.05. n = 4-5 animals/group.

**Supplementary Figure 4. Acute effects of juvenile LPS on hippocampal CA1 astrocytes and microglia**. (A-F) Two hours after SAL or LPS injection at PD22, GFAP and Iba1-positive cells were analyzed in the CA1. Astrocyte (GFAP-positive cells) density was determined in the *stratum oriens* (A), pyramidal cell layer (B), and *stratum radiatum* (C) of the CA1. Two-way ANOVA main effect: *** p < 0.001. Microglial (Iba1-positive) cell density was quantified in the *stratum oriens* (D), pyramidal cell layer (E), and *stratum radiatum* (F) of the CA1. Total cells, ramified cells, and hypertrophic cells are reported for each region. (G-L) At PD36 - two days after the last SAL or LPS injection - GFAP and Iba1-positive cells were analyzed in the CA1. Astrocyte (GFAP-positive cells) density was determined in the *stratum oriens* (G), pyramidal cell layer (H), and *stratum radiatum* (I) of the CA1. Microglial (Iba1-positive) cell density was quantified in the *stratum oriens* (J), pyramidal cell layer (K), and *stratum radiatum* (L) of the CA1. Total cells, ramified cells, and hypertrophic cells are reported for each region. n = 4-5 animals/group. Tukey´s posthoc comparison: * p < 0.05.

**Supplementary Figure 5. Acute effects of juvenile LPS on hippocampal CA1 astrocytes and microglia**. (A-F) Two hours after SAL or LPS injection at PD22, GFAP and Iba1-positive cells were analyzed in the CA1. Astrocyte (GFAP-positive cells) density was determined in the molecular layer (A), granular cell layer (B), and *hilus* (C) of the DG. Microglial (Iba1-positive) cell density was quantified in the molecular layer (D), granular cell layer (E), and *hilus* (F) of the DG. Total cells, ramified cells, and hypertrophic cells are reported for each region. (G-L) At PD36 - two days after the last SAL or LPS injection - GFAP and Iba1-positive cells were analyzed in the DG. Astrocyte (GFAP-positive cells) density was determined in the molecular layer (G), granular cell layer (H), and *hilus* (I) of the DG. Microglial (Iba1-positive) cell density was quantified in the molecular layer (J), granular cell layer (K), and *hilus* (L) of the DG. Total cells, ramified cells, and hypertrophic cells are reported for each region. n = 4-5 animals/group. Tukey’s posthoc comparison: ** p < 0.01.

**Supplementary Figure 6. Gestation and litter parameters.** (A) Gestational length was estimated from the observation of vaginal plug until delivery. (B) Total number of pups in each litter. (C) Number of males for each female within each litter. n_VEH_ = 22 litters, n_VPA_ = 16 litters.
