# Supplementary material for: Juvenile peripheral LPS exposure overrides female resilience to prenatal VPA effects on adult sociability in mice
Source: Sci Rep. 2024 May 19;14:11435. doi: 10.1038/s41598-024-62217-6 (PMC11102908; doi:10.1038/s41598-024-62217-6)
Supplement: Supplementary file 3 — Supplementary Information 2. [file 41598_2024_62217_MOESM3_ESM.html]

VPAxLPS\_RStatistics


# VPAxLPS\_RStatistics

### Libraries

```
library(reshape2)
library(TMB)
library(readxl)
library(glmmTMB)
```

```
## Warning in checkDepPackageVersion(dep_pkg = "TMB"): Package version inconsistency detected.
## glmmTMB was built with TMB version 1.9.6
## Current TMB version is 1.9.10
## Please re-install glmmTMB from source or restore original 'TMB' package (see '?reinstalling' for more information)
```

```
library(dplyr)
```

```
## 
## Attaching package: 'dplyr'
```

```
## The following objects are masked from 'package:stats':
## 
##     filter, lag
```

```
## The following objects are masked from 'package:base':
## 
##     intersect, setdiff, setequal, union
```

```
library(tidymodels)
```

```
## ── Attaching packages ────────────────────────────────────── tidymodels 1.1.0 ──
```

```
## ✔ broom        1.0.5     ✔ rsample      1.1.1
## ✔ dials        1.2.0     ✔ tibble       3.2.1
## ✔ ggplot2      3.4.3     ✔ tidyr        1.3.0
## ✔ infer        1.0.4     ✔ tune         1.1.1
## ✔ modeldata    1.2.0     ✔ workflows    1.1.3
## ✔ parsnip      1.1.0     ✔ workflowsets 1.0.1
## ✔ purrr        1.0.2     ✔ yardstick    1.2.0
## ✔ recipes      1.0.7
```

```
## ── Conflicts ───────────────────────────────────────── tidymodels_conflicts() ──
## ✖ purrr::discard() masks scales::discard()
## ✖ dplyr::filter()  masks stats::filter()
## ✖ dplyr::lag()     masks stats::lag()
## ✖ recipes::step()  masks stats::step()
## • Use suppressPackageStartupMessages() to eliminate package startup messages
```

```
library(tidyverse)
```

```
## ── Attaching core tidyverse packages ──────────────────────── tidyverse 2.0.0 ──
## ✔ forcats   1.0.0     ✔ readr     2.1.4
## ✔ lubridate 1.9.2     ✔ stringr   1.5.0
```

```
## ── Conflicts ────────────────────────────────────────── tidyverse_conflicts() ──
## ✖ readr::col_factor() masks scales::col_factor()
## ✖ purrr::discard()    masks scales::discard()
## ✖ dplyr::filter()     masks stats::filter()
## ✖ stringr::fixed()    masks recipes::fixed()
## ✖ dplyr::lag()        masks stats::lag()
## ✖ readr::spec()       masks yardstick::spec()
## ℹ Use the conflicted package (<http://conflicted.r-lib.org/>) to force all conflicts to become errors
```

```
library(emmeans)
library(car)
```

```
## Loading required package: carData
## 
## Attaching package: 'car'
## 
## The following object is masked from 'package:purrr':
## 
##     some
## 
## The following object is masked from 'package:dplyr':
## 
##     recode
```

```
library(DHARMa)
```

```
## This is DHARMa 0.4.6. For overview type '?DHARMa'. For recent changes, type news(package = 'DHARMa')
```

```
library(lme4)
```

```
## Loading required package: Matrix
## 
## Attaching package: 'Matrix'
## 
## The following objects are masked from 'package:tidyr':
## 
##     expand, pack, unpack
```

```
library(nlme)
```

```
## 
## Attaching package: 'nlme'
## 
## The following object is masked from 'package:lme4':
## 
##     lmList
## 
## The following object is masked from 'package:dplyr':
## 
##     collapse
```

```
library(reshape2)
library(lmerTest)
```

```
## 
## Attaching package: 'lmerTest'
## 
## The following object is masked from 'package:lme4':
## 
##     lmer
## 
## The following object is masked from 'package:recipes':
## 
##     step
## 
## The following object is masked from 'package:stats':
## 
##     step
```

```
library(tidyr)
library(multcomp)
```

```
## Loading required package: mvtnorm
## Loading required package: survival
## Loading required package: TH.data
## Loading required package: MASS
## 
## Attaching package: 'MASS'
## 
## The following object is masked from 'package:dplyr':
## 
##     select
## 
## 
## Attaching package: 'TH.data'
## 
## The following object is masked from 'package:MASS':
## 
##     geyser
```

# Figure 1

## Figure 1A: INTERLEUKIN-1BETA EXPRESSION AT PD22

```
#Read data
data_IL1 <- read_excel("VPAxLPS_Data.xlsx", sheet = "IL1_PD22")
data_IL1[data_IL1==""]<-NA
```

### SPLEEN

```
# Subset the data to separate animals with "SAL" and "LPS" in the "PD22" column
data_SAL <- subset(data_IL1, PD22 == "SAL")
data_LPS <- subset(data_IL1, PD22 == "LPS")

# Mixed-effects model
model1a <- lme(IL1_Spleen ~ PD22, random = ~ 1 | Dam, data=data_IL1)
Anova(model1a, type="III")
```

```
#Check for normality
shapiro.test(data_IL1$IL1_Spleen)
```

```
## 
##  Shapiro-Wilk normality test
## 
## data:  data_IL1$IL1_Spleen
## W = 0.91884, p-value = 0.497
```

```
#Check for homocedasticity
data_IL1$PD22 <- as.factor(data_IL1$PD22)
leveneTest(IL1_Spleen ~ PD22, data = data_IL1)
```

### HIPPOCAMPUS

```
# Subset the data to separate animals with "SAL" and "LPS" in the "PD22" column
data_SAL <- subset(data_IL1, PD22 == "SAL")
data_LPS <- subset(data_IL1, PD22 == "LPS")

# Mixed-effects model
model1b <- lme(IL1_Hippocampus ~ PD22, random = ~ 1 | Dam, data=data_IL1)
Anova(model1b,type="III")
```

```
#Check for normality
shapiro.test(data_IL1$IL1_Hippocampus)
```

```
## 
##  Shapiro-Wilk normality test
## 
## data:  data_IL1$IL1_Hippocampus
## W = 0.84341, p-value = 0.1391
```

```
#Check for homocedasticity
data_IL1$PD22 <- as.factor(data_IL1$PD22)
leveneTest(IL1_Hippocampus ~ PD22, data = data_IL1)
```

## Figure 1B: WEIGHT GAIN PD22-PD34

```
data_weight <- read_excel("VPAxLPS_Data.xlsx", sheet = "Weight_gain")
data_weight[data_weight==""]<-NA
```

```
#Fit linear model
data_weight_long<- melt(data_weight, id.vars = c('Dam','Mouse', 'Prenatal', 'Juvenile'))
data_weight_long <- data_weight_long %>% rename (juvenile_day = "variable", weight_gain = "value")
model1 <- lmer(log(weight_gain) ~ juvenile_day*Prenatal*Juvenile + (1|Mouse), data=data_weight_long) #Log transformation to adjust normality
Anova(model1, type="III")
```

```
# Evaluate homocedasticity in a residual vs. fitted plot
residuals <- resid(model1)
par(mfrow = c(1, 2))
plot(fitted(model1), residuals, xlab = "Fitted Values", ylab = "Residuals")
abline(h = 0, col = "red", lty = 2)  # Add a horizontal line at y = 0
#Evaluate the normality of residuals
Bi1<-ranef(model1)$Mouse$'(Intercept)' 
qqnorm(Bi1, cex.main=.8)
qqline(Bi1)
```

## ABSOLUTE WEIGHT AT PD34

```
data_weightb <- read_excel("VPAxLPS_Data.xlsx", sheet = "Weight")
data_weightb[data_weightb==""]<-NA
```

```
#Fit mixed-effects model using dam as random factor
model1b<-lme(Weight_PD34~Prenatal*Juvenile, data=data_weight, na.action=na.exclude, random=~1|Dam)
Anova(model1b, type="III")
```

```
#Normality and Homoscedasticity checks
e1b<-resid(model1b) # Pearson's residuals
pre1b<-predict(model1b) #predicted
shapiro.test(e1b)
```

```
## 
##  Shapiro-Wilk normality test
## 
## data:  e1b
## W = 0.97215, p-value = 0.2943
```

```
leveneTest(Weight_PD34~Prenatal*Juvenile, data=data_weight, na.action=na.exclude, random=~1|dam)
```

```
par(mfrow = c(1, 2))
plot(pre1b, e1b, xlab="Predicted", ylab="Pearson's residuals",main="Dispersion of RE vs PRED",cex.main=.8 )
abline(0,0)
qqnorm(e1b, cex.main=.8)
qqline(e1b)
```

# Figure 2 - ADULT BEHAVIOR

```
#Read the data
data_behavior <- read_excel("VPAxLPS_Data.xlsx", sheet = "Behavior")
data_SItest <- read_excel("VPAxLPS_Data.xlsx", sheet = "SI_test")
data_SIhab <- read_excel("VPAxLPS_Data.xlsx", sheet = "SI_habituation")
data_behavior[data_behavior==""]<-NA
data_behavior$Dam <- factor(data_behavior$Dam)
data_SItest[data_SItest==""]<-NA
data_SItest$Mouse <- factor(data_SItest$Mouse)
data_SItest$Stimulus <- factor(data_SItest$Stimulus)
data_SIhab[data_SIhab==""]<-NA
data_SIhab$Mouse <- factor(data_SIhab$Mouse)
data_SIhab$Stimulus <- factor(data_SIhab$Side)
```

## Figure 2B: SOCIAL INTERACTION - HABITUATION

```
# Subset the data to include only relevant observations
VEH_SAL_SIhab <- data_SIhab[data_SItest$Prenatal == "VEH" & data_SItest$Juvenile == "SAL", ]
VEH_LPS_SIhab <- data_SIhab[data_SItest$Prenatal == "VEH" & data_SItest$Juvenile == "LPS", ]
VPA_SAL_SIhab <- data_SIhab[data_SItest$Prenatal == "VPA" & data_SItest$Juvenile == "SAL", ]
VPA_LPS_SIhab <- data_SIhab[data_SItest$Prenatal == "VPA" & data_SItest$Juvenile == "LPS", ]

# Compared sniffing of R and L sides for each mouse using mixed-effects models with Dam as a random effect
model2a <- lme(Time ~ Side, random = ~ 1 | Mouse / Dam, data=VEH_SAL_SIhab)
Anova(model2a, type="III")
```

```
model2b <- lme(Time ~ Side, random = ~ 1 | Mouse / Dam, data=VEH_LPS_SIhab)
Anova(model2b, type="III")
```

```
model2c <- lme(Time ~ Side, random = ~ 1 | Mouse / Dam, data=VPA_SAL_SIhab)
Anova(model2c, type="III")
```

```
model2d <- lme(Time ~ Side, random = ~ 1 | Mouse / Dam, data=VPA_LPS_SIhab)
Anova(model2d, type="III")
```

## Figure 2C: SOCIAL INTERACTION - TEST

```
# Subset the data to include only relevant observations
VEH_SAL_SIlong <- data_SItest[data_SItest$Prenatal == "VEH" & data_SItest$Juvenile == "SAL", ]
VEH_LPS_SIlong <- data_SItest[data_SItest$Prenatal == "VEH" & data_SItest$Juvenile == "LPS", ]
VPA_SAL_SIlong <- data_SItest[data_SItest$Prenatal == "VPA" & data_SItest$Juvenile == "SAL", ]
VPA_LPS_SIlong <- data_SItest[data_SItest$Prenatal == "VPA" & data_SItest$Juvenile == "LPS", ]

# Compared sniffing of "SI_S" and "SI_NS" for each mouse using mixed-effects models nesting Mouse in Dam 
model2d <- lme(Time ~ Stimulus, random = ~ 1 | Mouse / Dam, data=VEH_SAL_SIlong)
Anova(model2d, type="III")
```

```
model2e <- lme(Time ~ Stimulus, random = ~ 1 | Mouse / Dam, data=VEH_LPS_SIlong)
Anova(model2e, type="III")
```

```
model2e <- lme(Time ~ Stimulus, random = ~ 1 | Mouse / Dam, data=VPA_SAL_SIlong)
Anova(model2e, type="III")
```

```
model2g <- lme(Time ~ Stimulus, random = ~ 1 | Mouse / Dam, data=VPA_LPS_SIlong)
Anova(model2g, type="III")
```

## Figure 2D: SOCIABILITY INDEX

```
#Fit mixed-effects model using dam as random factor
model2<-lme(SI_index~Prenatal*Juvenile, data=data_behavior, na.action=na.exclude, random=~1|Dam)
Anova(model2, type="III")
```

```
#Normality and Homoscedasticity checks
e2<-resid(model2) # Pearson's residuals
pre2<-predict(model2) #predicted
shapiro.test(e2)
```

```
## 
##  Shapiro-Wilk normality test
## 
## data:  e2
## W = 0.96466, p-value = 0.1738
```

```
leveneTest(SI_index~Prenatal*Juvenile, data=data_behavior, na.action=na.exclude, random=~1|dam)
```

```
par(mfrow = c(1, 2))
plot(pre2, e2, xlab="Predicted", ylab="Pearson's residuals",main="Dispersion of RE vs PRED",cex.main=.8 )
abline(0,0)
qqnorm(e2, cex.main=.8)
qqline(e2)
```

```
#Posthoc comparisons
emmeans(model2, pairwise~Prenatal*Juvenile)
```

```
## $emmeans
##  Prenatal Juvenile emmean   SE df lower.CL upper.CL
##  VEH      LPS        37.2 6.09 17    24.38     50.1
##  VPA      LPS        14.5 6.36 16     1.03     28.0
##  VEH      SAL        45.2 5.85 17    32.91     57.6
##  VPA      SAL        48.4 6.67 16    34.31     62.6
## 
## Degrees-of-freedom method: containment 
## Confidence level used: 0.95 
## 
## $contrasts
##  contrast          estimate   SE df t.ratio p.value
##  VEH LPS - VPA LPS    22.72 8.80 16   2.581  0.0846
##  VEH LPS - VEH SAL    -8.02 8.44 26  -0.951  0.7781
##  VEH LPS - VPA SAL   -11.22 9.03 16  -1.243  0.6100
##  VPA LPS - VEH SAL   -30.74 8.64 16  -3.559  0.0126
##  VPA LPS - VPA SAL   -33.94 9.21 26  -3.684  0.0055
##  VEH SAL - VPA SAL    -3.20 8.87 16  -0.361  0.9834
## 
## Degrees-of-freedom method: containment 
## P value adjustment: tukey method for comparing a family of 4 estimates
```

## Figure 2E: SELF-GROOMING

```
#Fit mixed-effects model using dam as random factor
model3<-lme(SG_time~Prenatal*Juvenile, data=data_behavior, na.action=na.exclude, random=~1|Dam)
Anova(model3, type="III")
```

```
#Normality and Homoscedasticity checks
e3<-resid(model3) # Pearson's residuals
pre3<-predict(model3) #predicted
shapiro.test(e3)
```

```
## 
##  Shapiro-Wilk normality test
## 
## data:  e3
## W = 0.98133, p-value = 0.6479
```

```
leveneTest(SG_time~Prenatal*Juvenile, data=data_behavior, na.action=na.exclude, random=~1|dam)
```

```
par(mfrow = c(1, 2))
plot(pre3, e3, xlab="Predicted", ylab="Pearson's residuals",main="Dispersion of RE vs PRED",cex.main=.8 )
abline(0,0)
qqnorm(e3, cex.main=.8)
qqline(e3)
```

## Figure 2F: OPEN FIELD - TOTAL DISTANCE

```
#Fit mixed-effects model using dam as random factor
model4<-lme(OF_Distance~Prenatal*Juvenile, data=data_behavior, na.action=na.exclude, random=~1|Dam)
Anova(model4, type="III")
```

```
#Normality and Homoscedasticity checks
e4<-resid(model4) # Pearson's residuals
pre4<-predict(model4) #predicted
shapiro.test(e4)
```

```
## 
##  Shapiro-Wilk normality test
## 
## data:  e4
## W = 0.97365, p-value = 0.3758
```

```
leveneTest(OF_Distance~Prenatal*Juvenile, data=data_behavior, na.action=na.exclude, random=~1|dam)
```

```
par(mfrow = c(1, 2))
plot(pre4, e4, xlab="Predicted", ylab="Pearson's residuals",main="Dispersion of RE vs PRED",cex.main=.8 )
abline(0,0)
qqnorm(e4, cex.main=.8)
qqline(e4)
```

## Figure 2G: OPEN FIELD - CENTER TIME

```
#Fit mixed-effects model using dam as random factor
model5 <- glmmTMB((as.integer(OF_Center_time)) ~ Prenatal * Juvenile + (1 | Dam),
                  data = data_behavior,
                  ziformula = ~ 1,
                  family = nbinom2, na.action = na.exclude)
Anova(model5, type="III")
```

```
#Normality and Homoscedasticity checks
e5<-resid(model5) # Pearson's residuals
pre5<-predict(model5) #predicted
shapiro.test(e5)
```

```
## 
##  Shapiro-Wilk normality test
## 
## data:  e5
## W = 0.98255, p-value = 0.7117
```

```
leveneTest(OF_Center_time~Prenatal*Juvenile, data=data_behavior, na.action=na.exclude, random=~1|dam)
```

```
par(mfrow = c(1, 2))
plot(pre5, e5, xlab="Predicted", ylab="Pearson's residuals",main="Dispersion of RE vs PRED",cex.main=.8 )
abline(0,0)
qqnorm(e5, cex.main=.8)
qqline(e5)
```

# Figure 3 - NEUROINFLAMMATION IN ADULT CEREBELLUM

## Figure 3A and B: GFAP IN ADULT CEREBELLUM

```
data_GFAPAdult <- read_excel("VPAxLPS_Data.xlsx", sheet = "GFAP_Adult")
data_GFAPAdult[data_GFAPAdult==""]<-NA
```

## Figure 3A: GFAP IN THE MOLECULAR LAYER

```
#Fit mixed-effects model with gamma distribution and analysis of the fixed effects
model6 <- glmmTMB(CerMol ~ Prenatal * Juvenile + (1 | Dam),
                     data = data_GFAPAdult,
                     family = Gamma(link = "log"),
                     na.action = na.exclude)
Anova(model6, type="III")
```

```
#Normality and Homocedasticity checks
e6<-resid(model6) # Pearson's residuals
pre6<-predict(model6) #predicted
shapiro.test(e6)
```

```
## 
##  Shapiro-Wilk normality test
## 
## data:  e6
## W = 0.73841, p-value = 0.0002315
```

```
leveneTest(CerMol~Prenatal*Juvenile, data=data_GFAPAdult, random=~1|dam)
```

```
par(mfrow = c(1, 2))
plot(pre6, e6, xlab="Predicted", ylab="Pearson's residuals",main="Dispersion of RE vs PRED",cex.main=.8 )
abline(0,0)
qqnorm(e6, cex.main=.8)
qqline(e6)
```

```
#Posthoc comparisons
emmeans(model6, pairwise~Prenatal*Juvenile)
```

```
## $emmeans
##  Prenatal Juvenile emmean    SE  df asymp.LCL asymp.UCL
##  VEH      LPS        2.55 0.116 Inf      2.32      2.78
##  VPA      LPS        2.45 0.116 Inf      2.22      2.68
##  VEH      SAL        2.07 0.116 Inf      1.85      2.30
##  VPA      SAL        2.56 0.116 Inf      2.34      2.79
## 
## Results are given on the log (not the response) scale. 
## Confidence level used: 0.95 
## 
## $contrasts
##  contrast          estimate     SE  df z.ratio p.value
##  VEH LPS - VPA LPS   0.1001 0.1646 Inf   0.608  0.9295
##  VEH LPS - VEH SAL   0.4796 0.0115 Inf  41.791  <.0001
##  VEH LPS - VPA SAL  -0.0122 0.1647 Inf  -0.074  0.9999
##  VPA LPS - VEH SAL   0.3794 0.1645 Inf   2.306  0.0966
##  VPA LPS - VPA SAL  -0.1124 0.0115 Inf  -9.793  <.0001
##  VEH SAL - VPA SAL  -0.4918 0.1646 Inf  -2.987  0.0149
## 
## Results are given on the log (not the response) scale. 
## P value adjustment: tukey method for comparing a family of 4 estimates
```

## Figure 3B: GFAP IN THE GRANULAR CELL LAYER

```
#Fit mixed-effects model with gamma distribution and analysis of the fixed effects
model7 <- glmmTMB(CerGran ~ Prenatal * Juvenile + (1 | Dam),
                     data = data_GFAPAdult,
                     family = Gamma(link = "log"),
                     na.action = na.exclude)
Anova(model7, type="III")
```

```
#Normality and Homocedasticity checks
e7<-resid(model7) # Pearson's residuals
pre7<-predict(model7) #predicted
shapiro.test(e7)
```

```
## 
##  Shapiro-Wilk normality test
## 
## data:  e7
## W = 0.92638, p-value = 0.1676
```

```
leveneTest(CerGran~Prenatal*Juvenile, data=data_GFAPAdult)
```

```
par(mfrow = c(1, 2))
plot(pre7, e7, xlab="Predicted", ylab="Pearson's residuals",main="Dispersion of RE vs PRED",cex.main=.8 )
abline(0,0)
qqnorm(e7, cex.main=.8)
qqline(e7)
```

```
#Posthoc comparisons
emmeans(model7, pairwise~Prenatal*Juvenile)
```

```
## $emmeans
##  Prenatal Juvenile emmean    SE  df asymp.LCL asymp.UCL
##  VEH      LPS        2.47 0.132 Inf      2.21      2.73
##  VPA      LPS        2.37 0.118 Inf      2.14      2.60
##  VEH      SAL        2.22 0.118 Inf      1.99      2.45
##  VPA      SAL        2.86 0.132 Inf      2.60      3.12
## 
## Results are given on the log (not the response) scale. 
## Confidence level used: 0.95 
## 
## $contrasts
##  contrast          estimate    SE  df z.ratio p.value
##  VEH LPS - VPA LPS   0.0998 0.178 Inf   0.562  0.9434
##  VEH LPS - VEH SAL   0.2513 0.178 Inf   1.415  0.4900
##  VEH LPS - VPA SAL  -0.3874 0.187 Inf  -2.070  0.1631
##  VPA LPS - VEH SAL   0.1515 0.167 Inf   0.905  0.8022
##  VPA LPS - VPA SAL  -0.4872 0.178 Inf  -2.743  0.0309
##  VEH SAL - VPA SAL  -0.6387 0.178 Inf  -3.597  0.0018
## 
## Results are given on the log (not the response) scale. 
## P value adjustment: tukey method for comparing a family of 4 estimates
```

## Figure 3C and D: IBA1 IN ADULT CEREBELLUM

```
data_IBA1Adult <- read_excel("VPAxLPS_Data.xlsx", sheet = "IBA1_Adult")
data_IBA1Adult[data_IBA1Adult==""]<-NA
```

## Figure 3C: IBA1 IN THE MOLECULAR LAYER

### Total cells

```
#Fit mixed-effects model using dam as random factor
model8<-lme(CerMol_total~Prenatal*Juvenile, data=data_IBA1Adult, na.action=na.exclude, random=~1|Dam)
Anova(model8,type="III")
```

```
#Normality and Homocedasticity checks
e8<-resid(model8) # Pearson's residuals
pre8<-predict(model8) #predicted
shapiro.test(e8)
```

```
## 
##  Shapiro-Wilk normality test
## 
## data:  e8
## W = 0.94645, p-value = 0.372
```

```
leveneTest(CerMol_total~Prenatal*Juvenile, data=data_IBA1Adult)
```

```
par(mfrow = c(1, 2))
plot(pre8, e8, xlab="Predicted", ylab="Pearson's residuals",main="Dispersion of RE vs PRED",cex.main=.8 )
abline(0,0)
qqnorm(e8, cex.main=.8)
qqline(e8)
```

```
#Posthoc comparisons
emmeans(model8, pairwise~Prenatal*Juvenile)
```

```
## $emmeans
##  Prenatal Juvenile emmean    SE df lower.CL upper.CL
##  VEH      LPS        4.31 0.395 13     3.45     5.16
##  VPA      LPS        4.07 0.353 12     3.30     4.83
##  VEH      SAL        3.64 0.353  2     2.12     5.16
##  VPA      SAL        5.31 0.395  2     3.61     7.00
## 
## Degrees-of-freedom method: containment 
## Confidence level used: 0.95 
## 
## $contrasts
##  contrast          estimate    SE df t.ratio p.value
##  VEH LPS - VPA LPS    0.240 0.529 12   0.453  0.9678
##  VEH LPS - VEH SAL    0.663 0.529  2   1.252  0.6605
##  VEH LPS - VPA SAL   -1.000 0.558  2  -1.792  0.4647
##  VPA LPS - VEH SAL    0.423 0.499  2   0.848  0.8326
##  VPA LPS - VPA SAL   -1.240 0.529  2  -2.342  0.3279
##  VEH SAL - VPA SAL   -1.663 0.529  2  -3.141  0.2087
## 
## Degrees-of-freedom method: containment 
## P value adjustment: tukey method for comparing a family of 4 estimates
```

### Ramified cells

```
#Fit mixed-effects model using dam as random factor
model9<-lme(CerMol_type1~Prenatal*Juvenile, data=data_IBA1Adult, na.action=na.exclude, random=~1|Dam)
Anova(model9,type="III")
```

```
#Normality and Homocedasticity checks
e9<-resid(model9) # Pearson's residuals
pre9<-predict(model9) #predicted
shapiro.test(e9)
```

```
## 
##  Shapiro-Wilk normality test
## 
## data:  e9
## W = 0.93602, p-value = 0.2475
```

```
leveneTest(log(CerMol_type1)~Prenatal*Juvenile, data=data_IBA1Adult)
```

```
par(mfrow = c(1, 2))
plot(pre9, e9, xlab="Predicted", ylab="Pearson's residuals",main="Dispersion of RE vs PRED",cex.main=.8 )
abline(0,0)
qqnorm(e9, cex.main=.8)
qqline(e9)
```

```
#Posthoc comparisons
emmeans(model9, pairwise~Prenatal*Juvenile)
```

```
## $emmeans
##  Prenatal Juvenile emmean    SE df lower.CL upper.CL
##  VEH      LPS        3.26 0.202 13     2.83      3.7
##  VPA      LPS        2.60 0.180 12     2.21      3.0
##  VEH      SAL        2.02 0.180  2     1.25      2.8
##  VPA      SAL        3.93 0.202  2     3.06      4.8
## 
## Degrees-of-freedom method: containment 
## Confidence level used: 0.95 
## 
## $contrasts
##  contrast          estimate    SE df t.ratio p.value
##  VEH LPS - VPA LPS    0.657 0.271 12   2.428  0.1242
##  VEH LPS - VEH SAL    1.238 0.271  2   4.573  0.1088
##  VEH LPS - VPA SAL   -0.671 0.285  2  -2.353  0.3257
##  VPA LPS - VEH SAL    0.580 0.255  2   2.275  0.3417
##  VPA LPS - VPA SAL   -1.329 0.271  2  -4.909  0.0956
##  VEH SAL - VPA SAL   -1.909 0.271  2  -7.053  0.0483
## 
## Degrees-of-freedom method: containment 
## P value adjustment: tukey method for comparing a family of 4 estimates
```

### Hypertrophic

```
#Fit mixed-effects model using dam as random factor
model10<-lme(CerMol_type2~Prenatal*Juvenile, data=data_IBA1Adult, na.action=na.exclude, random=~1|Dam)
Anova(model10,type="III")
```

```
#Normality and Homocedasticity checks
e10<-resid(model10) # Pearson's residuals
pre10<-predict(model10) #predicted
shapiro.test(e10)
```

```
## 
##  Shapiro-Wilk normality test
## 
## data:  e10
## W = 0.94404, p-value = 0.3392
```

```
leveneTest(CerMol_type2~Prenatal*Juvenile, data=data_IBA1Adult)
```

```
par(mfrow = c(1, 2))
plot(pre10, e10, xlab="Predicted", ylab="Pearson's residuals",main="Dispersion of RE vs PRED",cex.main=.8 )
abline(0,0)
qqnorm(e10, cex.main=.8)
qqline(e10)
```

## Figure 3D: IBA1 IN THE GRANULAR CELL LAYER

### Total cells

```
#Fit mixed-effects model using dam as random factor
model11<-lme(CerGran_total~Prenatal*Juvenile, data=data_IBA1Adult, na.action=na.exclude, random=~1|Dam)
Anova(model11, type="III")
```

```
#Normality and Homocedasticity checks
e11<-resid(model11) # Pearson's residuals
pre11<-predict(model11) #predicted
shapiro.test(e11)
```

```
## 
##  Shapiro-Wilk normality test
## 
## data:  e11
## W = 0.91263, p-value = 0.09575
```

```
leveneTest(CerGran_total~Prenatal*Juvenile, data=data_IBA1Adult)
```

```
par(mfrow = c(1, 2))
plot(pre11, e11, xlab="Predicted", ylab="Pearson's residuals",main="Dispersion of RE vs PRED",cex.main=.8 )
abline(0,0)
qqnorm(e11, cex.main=.8)
qqline(e11)
```

### Ramified cells

```
#Fit mixed-effects model using dam as random factor
model12<-lme(CerGran_type1~Prenatal*Juvenile, data=data_IBA1Adult, na.action=na.exclude, random=~1|Dam)
Anova(model12, type="III")
```

```
#Normality and Homocedasticity checks
e12<-resid(model12) # Pearson's residuals
pre12<-predict(model12) #predicted
shapiro.test(e12)
```

```
## 
##  Shapiro-Wilk normality test
## 
## data:  e12
## W = 0.92558, p-value = 0.1623
```

```
leveneTest(CerGran_type1~Prenatal*Juvenile, data=data_IBA1Adult)
```

```
par(mfrow = c(1, 2))
plot(pre12, e12, xlab="Predicted", ylab="Pearson's residuals",main="Dispersion of RE vs PRED",cex.main=.8 )
abline(0,0)
qqnorm(e12, cex.main=.8)
qqline(e12)
```

### Hypertrophic

```
#Fit mixed-effects model using dam as random factor. log transformation for normality
model13<-lme(log(CerGran_type2)~Prenatal*Juvenile, data=data_IBA1Adult, na.action=na.exclude, random=~1|Dam)
Anova(model13,type="III")
```

```
#Normality and Homocedasticity checks
e13<-resid(model13) # Pearson's residuals
pre13<-predict(model13) #predicted
shapiro.test(e13)
```

```
## 
##  Shapiro-Wilk normality test
## 
## data:  e13
## W = 0.98281, p-value = 0.9743
```

```
leveneTest(log(CerGran_type2)~Prenatal*Juvenile, data=data_IBA1Adult)
```

```
par(mfrow = c(1, 2))
plot(pre13, e13, xlab="Predicted", ylab="Pearson's residuals",main="Dispersion of RE vs PRED",cex.main=.8 )
abline(0,0)
qqnorm(e13, cex.main=.8)
qqline(e13)
```

## Figure 3E: SHOLL ANALYSIS MICROGLIA ADULT MOLECULAR LAYER OF THE CEREBELLUM

```
#Read data
data_ShollCerMol <- read_excel("VPAxLPS_Data.xlsx", sheet = "Sholl_CerMol_Ad")
data_ShollCerMol[data_ShollCerMol==""]<-NA

#Branches in the right format
keycol <- "diameter"
valuecol <- "branches"
gathercols <- c("d10", "d15", "d20", "d25", "d30", "d35", "d40", "d45", "d50", "d55", "d60", "d65")

sholl_CerMol <- pivot_longer(data_ShollCerMol, cols = all_of(gathercols), 
                      names_to = keycol, values_to = valuecol)
branchmediast.Mouse<-aggregate(branches~Prenatal+Juvenile+diameter+Mouse, sholl_CerMol,mean)

#Fit mixed-effects model using Mouse as random factor.
model14 <- lmer(branches ~ Prenatal*Juvenile*diameter + (1|Mouse), data=branchmediast.Mouse)
Anova(model14,type="III")
```

```
emm <- emmeans(model14, ~ Juvenile:diameter)
```

```
## NOTE: Results may be misleading due to involvement in interactions
```

```
pairs(emm, by = "diameter")
```

```
## diameter = d10:
##  contrast  estimate    SE   df t.ratio p.value
##  LPS - SAL  -0.2259 0.375 65.3  -0.603  0.5487
## 
## diameter = d15:
##  contrast  estimate    SE   df t.ratio p.value
##  LPS - SAL   0.0275 0.375 65.3   0.073  0.9417
## 
## diameter = d20:
##  contrast  estimate    SE   df t.ratio p.value
##  LPS - SAL   0.5695 0.375 65.3   1.520  0.1333
## 
## diameter = d25:
##  contrast  estimate    SE   df t.ratio p.value
##  LPS - SAL   0.7079 0.375 65.3   1.889  0.0633
## 
## diameter = d30:
##  contrast  estimate    SE   df t.ratio p.value
##  LPS - SAL   1.0084 0.375 65.3   2.691  0.0090
## 
## diameter = d35:
##  contrast  estimate    SE   df t.ratio p.value
##  LPS - SAL   0.7141 0.375 65.3   1.906  0.0611
## 
## diameter = d40:
##  contrast  estimate    SE   df t.ratio p.value
##  LPS - SAL   0.6428 0.375 65.3   1.715  0.0910
## 
## diameter = d45:
##  contrast  estimate    SE   df t.ratio p.value
##  LPS - SAL   0.3445 0.375 65.3   0.919  0.3613
## 
## diameter = d50:
##  contrast  estimate    SE   df t.ratio p.value
##  LPS - SAL   0.2187 0.375 65.3   0.584  0.5614
## 
## diameter = d55:
##  contrast  estimate    SE   df t.ratio p.value
##  LPS - SAL   0.1335 0.375 65.3   0.356  0.7227
## 
## diameter = d60:
##  contrast  estimate    SE   df t.ratio p.value
##  LPS - SAL   0.1688 0.375 65.3   0.450  0.6539
## 
## diameter = d65:
##  contrast  estimate    SE   df t.ratio p.value
##  LPS - SAL   0.0250 0.375 65.3   0.067  0.9470
## 
## Results are averaged over the levels of: Prenatal 
## Degrees-of-freedom method: kenward-roger
```

## Figure 3F: SHOLL ANALYSIS MICROGLIA ADULT GRANULAR CELL LAYER OF THE CEREBELLUM

```
#Read data
data_ShollCerGran <- read_excel("VPAxLPS_Data.xlsx", sheet = "Sholl_CerGran_Ad")
data_ShollCerGran[data_ShollCerGran==""]<-NA

#Branches in the right format
keycol <- "diameter"
valuecol <- "branches"
gathercols <- c("d10", "d15", "d20", "d25", "d30", "d35", "d40", "d45", "d50", "d55", "d60", "d65")

sholl_CerGran <- pivot_longer(data_ShollCerGran, cols = all_of(gathercols), 
                      names_to = keycol, values_to = valuecol)
branchmediastGran.Mouse<-aggregate(branches~Prenatal+Juvenile+diameter+Mouse, sholl_CerGran,mean)


#Fit mixed-effects model using Mouse as random factor.
model15 <- lmer(branches ~ Prenatal*Juvenile*diameter + (1|Mouse), data=branchmediastGran.Mouse)
Anova(model15, type="III")
```

## Figure 3G: MICROGLIAL CELLS SOMA SIZE IN THE MOLECULAR LAYER OF THE CEREBELLUM

```
#Read data
data_ShollCerMol_Av <- read_excel("VPAxLPS_Data.xlsx", sheet = "Sholl_CerMol_Ad_Av")
data_ShollCerMol_Av[data_ShollCerMol_Av==""]<-NA

#Fit mixed-effects model using Dam as random factor.
model16<-lme(CerMol_Soma_size~Prenatal*Juvenile, data=data_ShollCerMol_Av, na.action=na.exclude, random=~1|Dam)
Anova(model16, type="III")
```

```
#Normality and Homocedasticity checks
e16<-resid(model16) # Pearson's residuals
pre16<-predict(model16) #predicted
shapiro.test(e16)
```

```
## 
##  Shapiro-Wilk normality test
## 
## data:  e16
## W = 0.94581, p-value = 0.3632
```

```
leveneTest(log(CerMol_Soma_size)~Prenatal*Juvenile, data=data_ShollCerMol_Av)
```

```
par(mfrow = c(1, 2))
plot(pre16, e16, xlab="Predicted", ylab="Pearson's residuals",main="Dispersion of RE vs PRED",cex.main=.8 )
abline(0,0)
qqnorm(e16, cex.main=.8)
qqline(e16)
```

## Figure 3H: MICROGLIAL CELLS SOMA SIZE IN THE GRANULAR CELL LAYER OF THE CEREBELLUM

```
#Read data
data_ShollCerGran_Av <- read_excel("VPAxLPS_Data.xlsx", sheet = "Sholl_CerGran_Ad_Av")
data_ShollCerGran_Av[data_ShollCerGran_Av==""]<-NA

#Fit mixed-effects model using Dam as random factor.
model17<-lme(CerGran_Soma_size~Prenatal*Juvenile, data=data_ShollCerGran_Av, na.action=na.exclude, random=~1|Dam)
Anova(model17, type="III")
```

```
#Normality and Homocedasticity checks
e17<-resid(model17) # Pearson's residuals
pre17<-predict(model17) #predicted
shapiro.test(e17)
```

```
## 
##  Shapiro-Wilk normality test
## 
## data:  e17
## W = 0.95773, p-value = 0.5584
```

```
leveneTest(CerGran_Soma_size~Prenatal*Juvenile, data=data_ShollCerGran_Av)
```

```
par(mfrow = c(1, 2))
plot(pre17, e17, xlab="Predicted", ylab="Pearson's residuals",main="Dispersion of RE vs PRED",cex.main=.8 )
abline(0,0)
qqnorm(e17, cex.main=.8)
qqline(e17)
```

## Figure 3K: CALBINDIN-POSITIVE PURKINJE CELLS LINEAR DENSITY

```
data_Calb <- read_excel("VPAxLPS_Data.xlsx", sheet = "Calbindin")
data_Calb[data_Calb==""]<-NA
```

```
#Fit mixed-effects model using dam as random factor
modelCalb<-lme(Calbindin~Prenatal*Juvenile, data=data_Calb, na.action=na.exclude, random=~1|Dam)
Anova(modelCalb,type="III")
```

```
#Normality and Homocedasticity checks
eCalb<-resid(modelCalb) # Pearson's residuals
preCalb<-predict(modelCalb) #predicted
shapiro.test(eCalb)
```

```
## 
##  Shapiro-Wilk normality test
## 
## data:  eCalb
## W = 0.96667, p-value = 0.7332
```

```
leveneTest(Calbindin~Prenatal*Juvenile, data=data_Calb)
```

```
par(mfrow = c(1, 2))
plot(preCalb, eCalb, xlab="Predicted", ylab="Pearson's residuals",main="Dispersion of RE vs PRED",cex.main=.8 )
abline(0,0)
qqnorm(eCalb, cex.main=.8)
qqline(eCalb)
```

# Figure 4

## Figure 4A and B: GFAP IN THE CEREBELLUM AT PD22

```
data_GFAPpd22 <- read_excel("VPAxLPS_Data.xlsx", sheet = "GFAP_PD22")
#my_data <- read_excel("my_file.xlsx", sheet = "data")
data_GFAPpd22[data_GFAPpd22==""]<-NA
```

## Figure 4A: GFAP density in the molecular layer of the cerebellum at PD22

```
#Fit mixed-effects model with gamma distribution and analysis of the fixed effects.
model18 <- glmmTMB(CerMol ~ Prenatal * PD22 + (1 | Dam),
                     data = data_GFAPpd22,
                     family = Gamma(link = "log"),
                     na.action = na.exclude)
Anova(model18, type="III")
```

```
#Normality and Homocedasticity checks
e18<-resid(model18) # Pearson's residuals
pre18<-predict(model18) #predicted
shapiro.test(e18)
```

```
## 
##  Shapiro-Wilk normality test
## 
## data:  e18
## W = 0.86857, p-value = 0.02586
```

```
leveneTest(CerMol~Prenatal*PD22, data=data_GFAPpd22)
```

```
par(mfrow = c(1, 2))
plot(pre18, e18, xlab="Predicted", ylab="Pearson's residuals",main="Dispersion of RE vs PRED",cex.main=.8 )
abline(0,0)
qqnorm(e18, cex.main=.8)
qqline(e18)
```

## Figure 4B: GFAP density in the granular cell layer of the cerebellum at PD22

```
#Fit mixed-effects model using dam as random factor.Log transformation to adjust normality of data.
model19<-lme(log(CerGran)~Prenatal*PD22, data=data_GFAPpd22, na.action=na.exclude, random=~1|Dam)
Anova(model19, type="III")
```

```
#Normality and Homocedasticity checks
e19<-resid(model19) # Pearson's residuals
pre19<-predict(model19) #predicted
shapiro.test(e19)
```

```
## 
##  Shapiro-Wilk normality test
## 
## data:  e19
## W = 0.8997, p-value = 0.07949
```

```
leveneTest(log(CerGran)~Prenatal*PD22, data=data_GFAPpd22)
```

```
par(mfrow = c(1, 2))
plot(pre19, e19, xlab="Predicted", ylab="Pearson's residuals",main="Dispersion of RE vs PRED",cex.main=.8 )
abline(0,0)
qqnorm(e19, cex.main=.8)
qqline(e19)
```

## Figure 4C and D: IBA1 IN THE CEREBELLUM AT PD22

```
data_IBA1pd22 <- read_excel("VPAxLPS_Data.xlsx", sheet = "IBA1_PD22")
data_IBA1pd22[data_IBA1pd22==""]<-NA
```

## FIGURE 4C: IBA1 in cerebellar molecular layer at PD22

### Total cells

```
#Fit mixed-effects model using dam as random factor.
model20<-lme(CerMol_total~Prenatal*PD22, data=data_IBA1pd22, na.action=na.exclude, random=~1|Dam)
Anova(model20,type="III")
```

```
#Normality and Homocedasticity checks
e20<-resid(model20) # Pearson's residuals
pre20<-predict(model20) #predicted
shapiro.test(e20)
```

```
## 
##  Shapiro-Wilk normality test
## 
## data:  e20
## W = 0.97626, p-value = 0.9035
```

```
leveneTest(CerMol_total~Prenatal*PD22, data=data_IBA1pd22)
```

```
par(mfrow = c(1, 2))
plot(pre20, e20, xlab="Predicted", ylab="Pearson's residuals",main="Dispersion of RE vs PRED",cex.main=.8 )
abline(0,0)
qqnorm(e20, cex.main=.8)
qqline(e20)
```

### Ramified cells

```
#Fit mixed-effects model using dam as random factor.
model21<-lme(CerMol_type1~Prenatal*PD22, data=data_IBA1pd22, na.action=na.exclude, random=~1|Dam)
Anova(model21, type="III")
```

```
#Normality and Homocedasticity checks
e21<-resid(model21) # Pearson's residuals
pre21<-predict(model21) #predicted
shapiro.test(e21)
```

```
## 
##  Shapiro-Wilk normality test
## 
## data:  e21
## W = 0.96078, p-value = 0.6167
```

```
leveneTest(CerMol_type1~Prenatal*PD22, data=data_IBA1pd22)
```

```
par(mfrow = c(1, 2))
plot(pre21, e21, xlab="Predicted", ylab="Pearson's residuals",main="Dispersion of RE vs PRED",cex.main=.8 )
abline(0,0)
qqnorm(e21, cex.main=.8)
qqline(e21)
```

### Hypertrophic cells

```
#Fit mixed-effects model using dam as random factor. SQRT transformation applied for normalizing the data.
model22<-lme(sqrt(CerMol_type2)~Prenatal*PD22, data=data_IBA1pd22, na.action=na.exclude, random=~1|Dam)
Anova(model22,type="III")
```

```
#Normality and Homocedasticity checks
e22<-resid(model22) # Pearson's residuals
pre22<-predict(model22) #predicted
shapiro.test(e22)
```

```
## 
##  Shapiro-Wilk normality test
## 
## data:  e22
## W = 0.91083, p-value = 0.08898
```

```
leveneTest(sqrt(CerMol_type2)~Prenatal*PD22, data=data_IBA1pd22)
```

```
par(mfrow = c(1, 2))
plot(pre22, e22, xlab="Predicted", ylab="Pearson's residuals",main="Dispersion of RE vs PRED",cex.main=.8 )
abline(0,0)
qqnorm(e22, cex.main=.8)
qqline(e22)
```

## FIGURE 4D: IBA1 in cerebellar granular cell layer at PD22

### Total cells

```
#Fit mixed-effects model using dam as random factor.
model23<-lme(CerGran_total~Prenatal*PD22, data=data_IBA1pd22, na.action=na.exclude, random=~1|Dam)
Anova(model23,type="III")
```

```
#Normality and Homocedasticity checks
e23<-resid(model23) # Pearson's residuals
pre23<-predict(model23) #predicted
shapiro.test(e23)
```

```
## 
##  Shapiro-Wilk normality test
## 
## data:  e23
## W = 0.97317, p-value = 0.8547
```

```
leveneTest(CerGran_total~Prenatal*PD22, data=data_IBA1pd22)
```

```
par(mfrow = c(1, 2))
plot(pre23, e23, xlab="Predicted", ylab="Pearson's residuals",main="Dispersion of RE vs PRED",cex.main=.8 )
abline(0,0)
qqnorm(e23, cex.main=.8)
qqline(e23)
```

### Ramified cells

```
#Fit mixed-effects model using dam as random factor.
model24<-lme(CerGran_type1~Prenatal*PD22, data=data_IBA1pd22, na.action=na.exclude, random=~1|Dam)
Anova(model24,type="III")
```

```
#Normality and Homocedasticity checks
e24<-resid(model24) # Pearson's residuals
pre24<-predict(model24) #predicted
shapiro.test(e24)
```

```
## 
##  Shapiro-Wilk normality test
## 
## data:  e24
## W = 0.97518, p-value = 0.8874
```

```
leveneTest(CerGran_type1~Prenatal*PD22, data=data_IBA1pd22)
```

```
par(mfrow = c(1, 2))
plot(pre24, e24, xlab="Predicted", ylab="Pearson's residuals",main="Dispersion of RE vs PRED",cex.main=.8 )
abline(0,0)
qqnorm(e24, cex.main=.8)
qqline(e24)
```

### Hypertrophic cells

```
#Fit mixed-effects model using dam as random factor. Log transformation applied for normalizing the data.
model25<-lme(log(1+CerGran_type2)~Prenatal*PD22, data=data_IBA1pd22, na.action=na.exclude, random=~1|Dam)
Anova(model25,type="III")
```

```
#Normality and Homocedasticity checks
e25<-resid(model25) # Pearson's residuals
pre25<-predict(model25) #predicted
shapiro.test(e25)
```

```
## 
##  Shapiro-Wilk normality test
## 
## data:  e25
## W = 0.85748, p-value = 0.01114
```

```
leveneTest(CerGran_type2~Prenatal*PD22, data=data_IBA1pd22)
```

```
par(mfrow = c(1, 2))
plot(pre25, e25, xlab="Predicted", ylab="Pearson's residuals",main="Dispersion of RE vs PRED",cex.main=.8 )
abline(0,0)
qqnorm(e25, cex.main=.8)
qqline(e25)
```

## Figure 4E and F: GFAP IN THE CEREBELLUM AT PD36

```
data_GFAPpd36 <- read_excel("VPAxLPS_Data.xlsx", sheet = "GFAP_PD36")
data_GFAPpd36[data_GFAPpd36==""]<-NA
```

## Figure 4E: GFAP density in the molecular layer of the cerebellum at PD36

```
#Fit mixed-effects model using dam as random factor
model26<-lme(CerMol~Prenatal*Juvenile, data=data_GFAPpd36, na.action=na.exclude, random=~1|Dam)
Anova(model26,type="III")
```

```
#Normality and Homocedasticity checks
e26<-resid(model26) # Pearson's residuals
pre26<-predict(model26) #predicted
shapiro.test(e26)
```

```
## 
##  Shapiro-Wilk normality test
## 
## data:  e26
## W = 0.9582, p-value = 0.5673
```

```
leveneTest(CerMol~Prenatal*Juvenile, data=data_GFAPpd36)
```

```
par(mfrow = c(1, 2))
plot(pre26, e26, xlab="Predicted", ylab="Pearson's residuals",main="Dispersion of RE vs PRED",cex.main=.8 )
abline(0,0)
qqnorm(e26, cex.main=.8)
qqline(e26)
```

## Figure 4F: GFAP density in the granular cell layer of the cerebellum at PD36

```
#Fit mixed-effects model using dam as random factor
model27<-lme(CerGran~Prenatal*Juvenile, data=data_GFAPpd36, na.action=na.exclude, random=~1|Dam)
Anova(model27,type="III")
```

```
#Normality and Homocedasticity checks
e27<-resid(model27) # Pearson's residuals
pre27<-predict(model27) #predicted
shapiro.test(e27)
```

```
## 
##  Shapiro-Wilk normality test
## 
## data:  e27
## W = 0.95108, p-value = 0.4422
```

```
leveneTest(CerGran~Prenatal*Juvenile, data=data_GFAPpd36)
```

```
par(mfrow = c(1, 2))
plot(pre27, e27, xlab="Predicted", ylab="Pearson's residuals",main="Dispersion of RE vs PRED",cex.main=.8 )
abline(0,0)
qqnorm(e27, cex.main=.8)
qqline(e27)
```

## Figure 4G and H: IBA1 IN THE CEREBELLUM AT PD36

```
data_IBA1pd36 <- read_excel("VPAxLPS_Data.xlsx", sheet = "IBA1_PD36")
data_IBA1pd36[data_IBA1pd36==""]<-NA
```

## FIGURE 4G: IBA1 in cerebellar molecular layer at PD36

### Total cells

```
#Fit mixed-effects model using dam as random factor
model28<-lme(CerMol_total~Prenatal*Juvenile, data=data_IBA1pd36, na.action=na.exclude, random=~1|Dam)
Anova(model28,type="III")
```

```
#Normality and Homocedasticity checks
e28<-resid(model28) # Pearson's residuals
pre28<-predict(model28) #predicted
shapiro.test(e28)
```

```
## 
##  Shapiro-Wilk normality test
## 
## data:  e28
## W = 0.94731, p-value = 0.3844
```

```
leveneTest(CerMol_total~Prenatal*Juvenile, data=data_IBA1pd36)
```

```
par(mfrow = c(1, 2))
plot(pre28, e28, xlab="Predicted", ylab="Pearson's residuals",main="Dispersion of RE vs PRED",cex.main=.8 )
abline(0,0)
qqnorm(e28, cex.main=.8)
qqline(e28)
```

### Ramified cells

```
#Fit mixed-effects model using dam as random factor
model29<-lme(CerMol_type1~Prenatal*Juvenile, data=data_IBA1pd36, na.action=na.exclude, random=~1|Dam)
Anova(model29,type="III")
```

```
#Normality and Homocedasticity checks
e29<-resid(model29) # Pearson's residuals
pre29<-predict(model29) #predicted
shapiro.test(e29)
```

```
## 
##  Shapiro-Wilk normality test
## 
## data:  e29
## W = 0.96026, p-value = 0.6066
```

```
leveneTest(CerMol_type1~Prenatal*Juvenile, data=data_IBA1pd36)
```

```
par(mfrow = c(1, 2))
plot(pre29, e29, xlab="Predicted", ylab="Pearson's residuals",main="Dispersion of RE vs PRED",cex.main=.8 )
abline(0,0)
qqnorm(e29, cex.main=.8)
qqline(e29)
```

### Hypertrophic cells

```
#Fit mixed-effects model using dam as random factor
model30<-lme(CerMol_type2~Prenatal*Juvenile, data=data_IBA1pd36, na.action=na.exclude, random=~1|Dam)
Anova(model30,type="III")
```

```
#Normality and Homocedasticity checks
e30<-resid(model30) # Pearson's residuals
pre30<-predict(model30) #predicted
shapiro.test(e30)
```

```
## 
##  Shapiro-Wilk normality test
## 
## data:  e30
## W = 0.91417, p-value = 0.1019
```

```
leveneTest(CerMol_type2~Prenatal*Juvenile, data=data_IBA1pd36)
```

```
par(mfrow = c(1, 2))
plot(pre30, e30, xlab="Predicted", ylab="Pearson's residuals",main="Dispersion of RE vs PRED",cex.main=.8 )
abline(0,0)
qqnorm(e30, cex.main=.8)
qqline(e30)
```

## FIGURE 4H: IBA1 in cerebellar granular cell layer at PD36

### Total cells

```
#Fit mixed-effects model using dam as random factor
model31<-lme(CerGran_total~Prenatal*Juvenile, data=data_IBA1pd36, na.action=na.exclude, random=~1|Dam)
Anova(model31,type="III")
```

```
#Normality and Homocedasticity checks
e31<-resid(model31) # Pearson's residuals
pre31<-predict(model31) #predicted
shapiro.test(e31)
```

```
## 
##  Shapiro-Wilk normality test
## 
## data:  e31
## W = 0.93798, p-value = 0.2675
```

```
leveneTest(CerGran_total~Prenatal*Juvenile, data=data_IBA1pd36)
```

```
par(mfrow = c(1, 2))
plot(pre31, e31, xlab="Predicted", ylab="Pearson's residuals",main="Dispersion of RE vs PRED",cex.main=.8 )
abline(0,0)
qqnorm(e31, cex.main=.8)
qqline(e31)
```

### Ramified cells

```
#Fit mixed-effects model using dam as random factor
#model32<-gls(CerGran_type1~Prenatal*Juvenile, data=data_IBA1pd36, na.action=na.exclude)
model32<-lme(CerGran_type1~Prenatal*Juvenile, data=data_IBA1pd36, na.action=na.exclude, random=~1|Dam)
Anova(model32,type="III")
```

```
#Normality and Homocedasticity checks
e32<-resid(model32) # Pearson's residuals
pre32<-predict(model32) #predicted
shapiro.test(e32)
```

```
## 
##  Shapiro-Wilk normality test
## 
## data:  e32
## W = 0.93637, p-value = 0.2509
```

```
leveneTest(CerGran_type1~Prenatal*Juvenile, data=data_IBA1pd36)
```

```
par(mfrow = c(1, 2))
plot(pre32, e32, xlab="Predicted", ylab="Pearson's residuals",main="Dispersion of RE vs PRED",cex.main=.8 )
abline(0,0)
qqnorm(e32, cex.main=.8)
qqline(e32)
```

### Hypertrophic cells

```
#Fit mixed-effects model using dam as random factor
model33<-lme(CerGran_type2~Prenatal*Juvenile, data=data_IBA1pd36, na.action=na.exclude, random=~1|Dam)
Anova(model33,type="III")
```

```
#Normality and Homocedasticity checks
e33<-resid(model33) # Pearson's residuals
pre33<-predict(model33) #predicted
shapiro.test(e33)
```

```
## 
##  Shapiro-Wilk normality test
## 
## data:  e33
## W = 0.94945, p-value = 0.4165
```

```
leveneTest(CerGran_type2~Prenatal*Juvenile, data=data_IBA1pd36)
```

```
par(mfrow = c(1, 2))
plot(pre33, e33, xlab="Predicted", ylab="Pearson's residuals",main="Dispersion of RE vs PRED",cex.main=.8 )
abline(0,0)
qqnorm(e33, cex.main=.8)
qqline(e33)
```
