# Supplementary material for: Juvenile peripheral LPS exposure overrides female resilience to prenatal VPA effects on adult sociability in mice
Source: Sci Rep. 2024 May 19;14:11435. doi: 10.1038/s41598-024-62217-6 (PMC11102908; doi:10.1038/s41598-024-62217-6)
Supplement: Supplementary file 4 — Supplementary Information 3. [file 41598_2024_62217_MOESM4_ESM.html]

VPAxLPS\_RStatistics\_Suppl


# VPAxLPS\_RStatistics\_Suppl

#### 2024-02-16

### Libraries

```
library(reshape2)
library(TMB)
library(readxl)
library(glmmTMB)
```

```
## Warning in checkDepPackageVersion(dep_pkg = "TMB"): Package version inconsistency detected.
## glmmTMB was built with TMB version 1.9.6
## Current TMB version is 1.9.10
## Please re-install glmmTMB from source or restore original 'TMB' package (see '?reinstalling' for more information)
```

```
library(dplyr)
```

```
## 
## Attaching package: 'dplyr'
```

```
## The following objects are masked from 'package:stats':
## 
##     filter, lag
```

```
## The following objects are masked from 'package:base':
## 
##     intersect, setdiff, setequal, union
```

```
library(tidymodels)
```

```
## ── Attaching packages ────────────────────────────────────── tidymodels 1.1.0 ──
```

```
## ✔ broom        1.0.5     ✔ rsample      1.1.1
## ✔ dials        1.2.0     ✔ tibble       3.2.1
## ✔ ggplot2      3.4.3     ✔ tidyr        1.3.0
## ✔ infer        1.0.4     ✔ tune         1.1.1
## ✔ modeldata    1.2.0     ✔ workflows    1.1.3
## ✔ parsnip      1.1.0     ✔ workflowsets 1.0.1
## ✔ purrr        1.0.2     ✔ yardstick    1.2.0
## ✔ recipes      1.0.7
```

```
## ── Conflicts ───────────────────────────────────────── tidymodels_conflicts() ──
## ✖ purrr::discard() masks scales::discard()
## ✖ dplyr::filter()  masks stats::filter()
## ✖ dplyr::lag()     masks stats::lag()
## ✖ recipes::step()  masks stats::step()
## • Dig deeper into tidy modeling with R at https://www.tmwr.org
```

```
library(tidyverse)
```

```
## ── Attaching core tidyverse packages ──────────────────────── tidyverse 2.0.0 ──
## ✔ forcats   1.0.0     ✔ readr     2.1.4
## ✔ lubridate 1.9.2     ✔ stringr   1.5.0
```

```
## ── Conflicts ────────────────────────────────────────── tidyverse_conflicts() ──
## ✖ readr::col_factor() masks scales::col_factor()
## ✖ purrr::discard()    masks scales::discard()
## ✖ dplyr::filter()     masks stats::filter()
## ✖ stringr::fixed()    masks recipes::fixed()
## ✖ dplyr::lag()        masks stats::lag()
## ✖ readr::spec()       masks yardstick::spec()
## ℹ Use the conflicted package (<http://conflicted.r-lib.org/>) to force all conflicts to become errors
```

```
library(emmeans)
library(car)
```

```
## Loading required package: carData
## 
## Attaching package: 'car'
## 
## The following object is masked from 'package:purrr':
## 
##     some
## 
## The following object is masked from 'package:dplyr':
## 
##     recode
```

```
library(DHARMa)
```

```
## This is DHARMa 0.4.6. For overview type '?DHARMa'. For recent changes, type news(package = 'DHARMa')
```

```
library(lme4)
```

```
## Loading required package: Matrix
## 
## Attaching package: 'Matrix'
## 
## The following objects are masked from 'package:tidyr':
## 
##     expand, pack, unpack
```

```
library(nlme)
```

```
## 
## Attaching package: 'nlme'
## 
## The following object is masked from 'package:lme4':
## 
##     lmList
## 
## The following object is masked from 'package:dplyr':
## 
##     collapse
```

```
library(reshape2)
library(lmerTest)
```

```
## 
## Attaching package: 'lmerTest'
## 
## The following object is masked from 'package:lme4':
## 
##     lmer
## 
## The following object is masked from 'package:recipes':
## 
##     step
## 
## The following object is masked from 'package:stats':
## 
##     step
```

```
library(tidyr)
```

# SUPPLEMENTARY FIGURE 1: NEUROINFLAMMATION IN THE ADULT HIPPOCAMPAL CA1

```
data_GFAPAdult <- read_excel("VPAxLPS_Data.xlsx", sheet = "GFAP_Adult")
data_GFAPAdult[data_GFAPAdult==""]<-NA
data_IBA1Adult <- read_excel("VPAxLPS_Data.xlsx", sheet = "IBA1_Adult")
data_IBA1Adult[data_IBA1Adult==""]<-NA
```

## SUPPLEMENTARY FIGURE 1A: GFAP in the Stratum oriens of the CA1 at adulthood

```
#Fit mixed-effects model using dam as random factor
model1<-lme(CA1or~Prenatal*Juvenile, data=data_GFAPAdult, na.action=na.exclude, random=~1|Dam)
Anova(model1, type="III")
```

```
## Analysis of Deviance Table (Type III tests)
## 
## Response: CA1or
##                      Chisq Df Pr(>Chisq)    
## (Intercept)       365.8649  1     <2e-16 ***
## Prenatal            0.2946  1     0.5873    
## Juvenile            0.0742  1     0.7854    
## Prenatal:Juvenile   0.4947  1     0.4818    
## ---
## Signif. codes:  0 '***' 0.001 '**' 0.01 '*' 0.05 '.' 0.1 ' ' 1
```

```
#Normality and Homocedasticity checks
e1<-resid(model1) # Pearson's residuals
pre1<-predict(model1) #predicted
shapiro.test(e1)
```

```
## 
##  Shapiro-Wilk normality test
## 
## data:  e1
## W = 0.95492, p-value = 0.477
```

```
leveneTest(CA1or~Prenatal*Juvenile, data=data_GFAPAdult)
```

```
## Levene's Test for Homogeneity of Variance (center = median)
##       Df F value Pr(>F)
## group  3  0.5417 0.6611
##       15
```

```
par(mfrow = c(1, 2))
plot(pre1, e1, xlab="Predicted", ylab="Pearson's residuals",main="Dispersion of RE vs PRED",cex.main=.8 )
abline(0,0)
qqnorm(e1, cex.main=.8)
qqline(e1)
```

## SUPPLEMENTARY FIGURE 1B: GFAP in the Pyramidal cell layer of the CA1 at adulthood

```
#Fit mixed-effects model using dam as random factor
model2<-lme(CA1pyr~Prenatal*Juvenile, data=data_GFAPAdult, na.action=na.exclude, random=~1|Dam)
Anova(model2, type = "III")
```

```
## Analysis of Deviance Table (Type III tests)
## 
## Response: CA1pyr
##                      Chisq Df Pr(>Chisq)    
## (Intercept)       176.2653  1  < 2.2e-16 ***
## Prenatal            7.0906  1   0.007749 ** 
## Juvenile            0.3986  1   0.527820    
## Prenatal:Juvenile   0.0193  1   0.889377    
## ---
## Signif. codes:  0 '***' 0.001 '**' 0.01 '*' 0.05 '.' 0.1 ' ' 1
```

```
#Normality and Homocedasticity checks
e2<-resid(model2) # Pearson's residuals
pre2<-predict(model2) #predicted
shapiro.test(e2)
```

```
## 
##  Shapiro-Wilk normality test
## 
## data:  e2
## W = 0.95591, p-value = 0.4947
```

```
leveneTest(CA1pyr~Prenatal*Juvenile, data=data_GFAPAdult)
```

```
## Levene's Test for Homogeneity of Variance (center = median)
##       Df F value Pr(>F)
## group  3  0.1507 0.9276
##       15
```

```
par(mfrow = c(1, 2))
plot(pre2, e2, xlab="Predicted", ylab="Pearson's residuals",main="Dispersion of RE vs PRED",cex.main=.8 )
abline(0,0)
qqnorm(e2, cex.main=.8)
qqline(e2)
```

## SUPPLEMENTARY FIGURE 1C: GFAP in the Stratum radiatum of the CA1 at adulthood

```
#Fit mixed-effects model using dam as random factor
model3<-lme(CA1rad~Prenatal*Juvenile, data=data_GFAPAdult, na.action=na.exclude, random=~1|Dam)
Anova(model3, type="III")
```

```
## Analysis of Deviance Table (Type III tests)
## 
## Response: CA1rad
##                      Chisq Df Pr(>Chisq)    
## (Intercept)       320.3969  1     <2e-16 ***
## Prenatal            0.1408  1     0.7075    
## Juvenile            0.1561  1     0.6928    
## Prenatal:Juvenile   0.1174  1     0.7318    
## ---
## Signif. codes:  0 '***' 0.001 '**' 0.01 '*' 0.05 '.' 0.1 ' ' 1
```

```
#Normality and Homogeneity of variance checks
e3<-resid(model3) # Pearson's residuals
pre3<-predict(model3) #predicted

shapiro.test(e3)
```

```
## 
##  Shapiro-Wilk normality test
## 
## data:  e3
## W = 0.98285, p-value = 0.9702
```

```
leveneTest(CA1rad~Prenatal*Juvenile, data=data_GFAPAdult)
```

```
## Levene's Test for Homogeneity of Variance (center = median)
##       Df F value Pr(>F)
## group  3  0.4485  0.722
##       15
```

```
par(mfrow = c(1, 2))
plot(pre3, e3, xlab="Predicted", ylab="Pearson's residuals",main="Dispersion of RE vs PRED",cex.main=.8 )
abline(0,0)
qqnorm(e3, cex.main=.8)
qqline(e3)
```

## SUPPLEMENTARY FIGURE 1D: IBA1 in the Stratum oriens of the CA1 at adulthood

### Total cells

```
#Fit mixed-effects model using dam as random factor
model4<-lme(CA1or_total~Prenatal*Juvenile, data=data_IBA1Adult, na.action=na.exclude, random=~1|Dam)
Anova(model4, type="III")
```

```
## Analysis of Deviance Table (Type III tests)
## 
## Response: CA1or_total
##                      Chisq Df Pr(>Chisq)    
## (Intercept)       117.3179  1     <2e-16 ***
## Prenatal            0.1265  1     0.7221    
## Juvenile            0.0280  1     0.8670    
## Prenatal:Juvenile   0.4142  1     0.5199    
## ---
## Signif. codes:  0 '***' 0.001 '**' 0.01 '*' 0.05 '.' 0.1 ' ' 1
```

```
#Normality and Homocedasticity checks
e4<-resid(model4) # Pearson's residuals
pre4<-predict(model4) #predicted

shapiro.test(e4)
```

```
## 
##  Shapiro-Wilk normality test
## 
## data:  e4
## W = 0.9409, p-value = 0.2737
```

```
leveneTest(CA1or_total~Prenatal*Juvenile, data=data_IBA1Adult)
```

```
## Levene's Test for Homogeneity of Variance (center = median)
##       Df F value Pr(>F)
## group  3  1.1219 0.3715
##       15
```

```
par(mfrow = c(1, 2))
plot(pre4, e4, xlab="Predicted", ylab="Pearson's residuals",main="Dispersion of RE vs PRED",cex.main=.8 )
abline(0,0)
qqnorm(e4, cex.main=.8)
qqline(e4)
```

### Ramified cells

```
#Fit mixed-effects model using dam as random factor
model5<-lme(CA1or_type1~Prenatal*Juvenile, data=data_IBA1Adult, na.action=na.exclude, random=~1|Dam)
Anova(model5, type="III")
```

```
## Analysis of Deviance Table (Type III tests)
## 
## Response: CA1or_type1
##                      Chisq Df Pr(>Chisq)    
## (Intercept)       105.6284  1     <2e-16 ***
## Prenatal            0.1468  1     0.7016    
## Juvenile            0.1212  1     0.7277    
## Prenatal:Juvenile   0.3145  1     0.5749    
## ---
## Signif. codes:  0 '***' 0.001 '**' 0.01 '*' 0.05 '.' 0.1 ' ' 1
```

```
#Normality and Homocedasticity checks
e5<-resid(model5) # Pearson's residuals
pre5<-predict(model5) #predicted

shapiro.test(e5)
```

```
## 
##  Shapiro-Wilk normality test
## 
## data:  e5
## W = 0.93472, p-value = 0.2115
```

```
leveneTest(CA1or_type1~Prenatal*Juvenile, data=data_IBA1Adult)
```

```
## Levene's Test for Homogeneity of Variance (center = median)
##       Df F value Pr(>F)
## group  3  1.3401 0.2988
##       15
```

```
par(mfrow = c(1, 2))
plot(pre5, e5, xlab="Predicted", ylab="Pearson's residuals",main="Dispersion of RE vs PRED",cex.main=.8 )
abline(0,0)
qqnorm(e5, cex.main=.8)
qqline(e5)
```

### Hypertrophic cells

```
#Fit mixed-effects model using dam as random factor
model6<-lme(CA1or_type2~Prenatal*Juvenile, data=data_IBA1Adult, na.action=na.exclude, random=~1|Dam)
Anova(model6,type="III")
```

```
## Analysis of Deviance Table (Type III tests)
## 
## Response: CA1or_type2
##                     Chisq Df Pr(>Chisq)   
## (Intercept)       10.5736  1   0.001147 **
## Prenatal           0.0431  1   0.835451   
## Juvenile           0.4146  1   0.519664   
## Prenatal:Juvenile  0.1243  1   0.724444   
## ---
## Signif. codes:  0 '***' 0.001 '**' 0.01 '*' 0.05 '.' 0.1 ' ' 1
```

```
#Normality and Homocedasticity checks
e6<-resid(model6) # Pearson's residuals
pre6<-predict(model6) #predicted
shapiro.test(e6)
```

```
## 
##  Shapiro-Wilk normality test
## 
## data:  e6
## W = 0.97756, p-value = 0.9105
```

```
leveneTest(CA1or_type2~Prenatal*Juvenile, data=data_IBA1Adult)
```

```
## Levene's Test for Homogeneity of Variance (center = median)
##       Df F value Pr(>F)
## group  3  0.3349 0.8004
##       15
```

```
par(mfrow = c(1, 2))
plot(pre6, e6, xlab="Predicted", ylab="Pearson's residuals",main="Dispersion of RE vs PRED",cex.main=.8 )
abline(0,0)
qqnorm(e6, cex.main=.8)
qqline(e6)
```

## SUPPLEMENTARY FIGURE 1E: IBA1 in the Pyramidal cell layer of the CA1 at adulthood

### Total cells

```
#Fit mixed-effects model using dam as random factor
model7<-lme(CA1pyr_total~Prenatal*Juvenile, data=data_IBA1Adult, na.action=na.exclude, random=~1|Dam)
Anova(model7, type="III")
```

```
## Analysis of Deviance Table (Type III tests)
## 
## Response: CA1pyr_total
##                     Chisq Df Pr(>Chisq)    
## (Intercept)       95.2922  1     <2e-16 ***
## Prenatal           0.2223  1     0.6373    
## Juvenile           0.0010  1     0.9747    
## Prenatal:Juvenile  0.0086  1     0.9262    
## ---
## Signif. codes:  0 '***' 0.001 '**' 0.01 '*' 0.05 '.' 0.1 ' ' 1
```

```
#Normality and Homocedasticity checks
e7<-resid(model7) # Pearson's residuals
pre7<-predict(model7) #predicted
shapiro.test(e7)
```

```
## 
##  Shapiro-Wilk normality test
## 
## data:  e7
## W = 0.98379, p-value = 0.9771
```

```
leveneTest(CA1pyr_total~Prenatal*Juvenile, data=data_IBA1Adult)
```

```
## Levene's Test for Homogeneity of Variance (center = median)
##       Df F value Pr(>F)
## group  3  1.2624 0.3228
##       15
```

```
par(mfrow = c(1, 2))
plot(pre7, e7, xlab="Predicted", ylab="Pearson's residuals",main="Dispersion of RE vs PRED",cex.main=.8 )
abline(0,0)
qqnorm(e7, cex.main=.8)
qqline(e7)
```

### Ramified cells

```
#Fit mixed-effects model using dam as random factor
model8<-lme(CA1pyr_type1~Prenatal*Juvenile, data=data_IBA1Adult, na.action=na.exclude, random=~1|Dam)
Anova(model8,type="III")
```

```
## Analysis of Deviance Table (Type III tests)
## 
## Response: CA1pyr_type1
##                     Chisq Df Pr(>Chisq)    
## (Intercept)       93.2971  1     <2e-16 ***
## Prenatal           0.2072  1     0.6490    
## Juvenile           0.0000  1     0.9963    
## Prenatal:Juvenile  0.0151  1     0.9022    
## ---
## Signif. codes:  0 '***' 0.001 '**' 0.01 '*' 0.05 '.' 0.1 ' ' 1
```

```
#Normality and Homocedasticity checks
e8<-resid(model8) # Pearson's residuals
pre8<-predict(model8) #predicted
shapiro.test(e8)
```

```
## 
##  Shapiro-Wilk normality test
## 
## data:  e8
## W = 0.96693, p-value = 0.7139
```

```
leveneTest(CA1pyr_type1~Prenatal*Juvenile, data=data_IBA1Adult)
```

```
## Levene's Test for Homogeneity of Variance (center = median)
##       Df F value Pr(>F)
## group  3   0.868 0.4793
##       15
```

```
par(mfrow = c(1, 2))
plot(pre8, e8, xlab="Predicted", ylab="Pearson's residuals",main="Dispersion of RE vs PRED",cex.main=.8 )
abline(0,0)
qqnorm(e8, cex.main=.8)
qqline(e8)
```

### Hypertrophic

#### These data have many ceros and are therefore not normal. The analysis proposed does not meet the assumption of normality, but zero-inflated models were no good fit.

```
#Fit mixed-effects model using dam as random factor
model9<-lme(log(1+CA1pyr_type2)~Prenatal*Juvenile, data=data_IBA1Adult, na.action=na.exclude, random=~1|Dam)
Anova(model9,type="III")
```

```
## Analysis of Deviance Table (Type III tests)
## 
## Response: log(1 + CA1pyr_type2)
##                    Chisq Df Pr(>Chisq)  
## (Intercept)       4.0829  1    0.04332 *
## Prenatal          0.0042  1    0.94853  
## Juvenile          0.0740  1    0.78566  
## Prenatal:Juvenile 0.0077  1    0.92992  
## ---
## Signif. codes:  0 '***' 0.001 '**' 0.01 '*' 0.05 '.' 0.1 ' ' 1
```

```
#Normality and Homocedasticity checks
e9<-resid(model9) # Pearson's residuals
pre9<-predict(model9) #predicted
shapiro.test(e9)
```

```
## 
##  Shapiro-Wilk normality test
## 
## data:  e9
## W = 0.79531, p-value = 0.0009778
```

```
leveneTest(CA1pyr_type2~Prenatal*Juvenile, data=data_IBA1Adult)
```

```
## Levene's Test for Homogeneity of Variance (center = median)
##       Df F value Pr(>F)
## group  3  0.1125 0.9514
##       15
```

```
par(mfrow = c(1, 2))
plot(pre9, e9, xlab="Predicted", ylab="Pearson's residuals",main="Dispersion of RE vs PRED",cex.main=.8 )
abline(0,0)
qqnorm(e9, cex.main=.8)
qqline(e9)
```

## SUPPLEMENTARY FIGURE 1F: IBA1 in the Stratum radiatum of the CA1 at adulthood

### Total cells

```
#Fit mixed-effects model using dam as random factor
model10<-lme(CA1rad_total~Prenatal*Juvenile, data=data_IBA1Adult, na.action=na.exclude, random=~1|Dam)
Anova(model10,type="III")
```

```
## Analysis of Deviance Table (Type III tests)
## 
## Response: CA1rad_total
##                      Chisq Df Pr(>Chisq)    
## (Intercept)       218.6837  1    < 2e-16 ***
## Prenatal            0.4520  1    0.50137    
## Juvenile            1.2825  1    0.25743    
## Prenatal:Juvenile   2.9914  1    0.08371 .  
## ---
## Signif. codes:  0 '***' 0.001 '**' 0.01 '*' 0.05 '.' 0.1 ' ' 1
```

```
#Normality and Homocedasticity checks
e10<-resid(model10) # Pearson's residuals
pre10<-predict(model10) #predicted
shapiro.test(e10)
```

```
## 
##  Shapiro-Wilk normality test
## 
## data:  e10
## W = 0.97678, p-value = 0.8989
```

```
leveneTest(CA1rad_total~Prenatal*Juvenile, data=data_IBA1Adult)
```

```
## Levene's Test for Homogeneity of Variance (center = median)
##       Df F value Pr(>F)
## group  3  0.3645 0.7796
##       15
```

```
par(mfrow = c(1, 2))
plot(pre10, e10, xlab="Predicted", ylab="Pearson's residuals",main="Dispersion of RE vs PRED",cex.main=.8 )
abline(0,0)
qqnorm(e10, cex.main=.8)
qqline(e10)
```

### Ramified cells

```
#Fit mixed-effects model using dam as random factor
model11<-lme(CA1rad_type1~Prenatal*Juvenile, data=data_IBA1Adult, na.action=na.exclude, random=~1|Dam)
Anova(model11,type="III")
```

```
## Analysis of Deviance Table (Type III tests)
## 
## Response: CA1rad_type1
##                      Chisq Df Pr(>Chisq)    
## (Intercept)       169.8762  1    < 2e-16 ***
## Prenatal            0.3102  1    0.57758    
## Juvenile            0.5882  1    0.44312    
## Prenatal:Juvenile   2.9623  1    0.08523 .  
## ---
## Signif. codes:  0 '***' 0.001 '**' 0.01 '*' 0.05 '.' 0.1 ' ' 1
```

```
#Normality and Homocedasticity checks
e11<-resid(model11) # Pearson's residuals
pre11<-predict(model11) #predicted
shapiro.test(e11)
```

```
## 
##  Shapiro-Wilk normality test
## 
## data:  e11
## W = 0.96895, p-value = 0.7553
```

```
leveneTest(CA1or_type1~Prenatal*Juvenile, data=data_IBA1Adult)
```

```
## Levene's Test for Homogeneity of Variance (center = median)
##       Df F value Pr(>F)
## group  3  1.3401 0.2988
##       15
```

```
par(mfrow = c(1, 2))
plot(pre11, e11, xlab="Predicted", ylab="Pearson's residuals",main="Dispersion of RE vs PRED",cex.main=.8 )
abline(0,0)
qqnorm(e11, cex.main=.8)
qqline(e11)
```

### Hypertrophic

```
#Fit mixed-effects model using dam as random factor
model12<-lme(CA1rad_type2~Prenatal*Juvenile, data=data_IBA1Adult, na.action=na.exclude, random=~1|Dam)
Anova(model12,type="III")
```

```
## Analysis of Deviance Table (Type III tests)
## 
## Response: CA1rad_type2
##                    Chisq Df Pr(>Chisq)   
## (Intercept)       8.3132  1   0.003936 **
## Prenatal          0.0287  1   0.865406   
## Juvenile          0.4294  1   0.512299   
## Prenatal:Juvenile 0.0353  1   0.850984   
## ---
## Signif. codes:  0 '***' 0.001 '**' 0.01 '*' 0.05 '.' 0.1 ' ' 1
```

```
#Normality and Homocedasticity checks
e12<-resid(model12) # Pearson's residuals
pre12<-predict(model12) #predicted
shapiro.test(e12)
```

```
## 
##  Shapiro-Wilk normality test
## 
## data:  e12
## W = 0.9162, p-value = 0.09628
```

```
leveneTest(CA1rad_type2~Prenatal*Juvenile, data=data_IBA1Adult)
```

```
## Levene's Test for Homogeneity of Variance (center = median)
##       Df F value Pr(>F)
## group  3  0.1494 0.9285
##       15
```

```
par(mfrow = c(1, 2))
plot(pre12, e12, xlab="Predicted", ylab="Pearson's residuals",main="Dispersion of RE vs PRED",cex.main=.8 )
abline(0,0)
qqnorm(e12, cex.main=.8)
qqline(e12)
```

# SUPPLEMENTARY FIGURE 2: NEUROINFLAMMATION IN THE ADULT HIPPOCAMPAL DG

```
data_GFAPAdult <- read_excel("VPAxLPS_Data.xlsx", sheet = "GFAP_Adult")
data_GFAPAdult[data_GFAPAdult==""]<-NA
data_IBA1Adult <- read_excel("VPAxLPS_Data.xlsx", sheet = "IBA1_Adult")
data_IBA1Adult[data_IBA1Adult==""]<-NA
```

## SUPPLEMENTARY FIGURE 2A: GFAP in the Molecular layer of the DG at adulthood

```
#Fit mixed-effects model using dam as random factor
model13<-lme(DGmol~Prenatal*Juvenile, data=data_GFAPAdult, na.action=na.exclude, random=~1|Dam)
Anova(model13, type="III")
```

```
## Analysis of Deviance Table (Type III tests)
## 
## Response: DGmol
##                      Chisq Df Pr(>Chisq)    
## (Intercept)       326.4330  1  < 2.2e-16 ***
## Prenatal            5.0763  1   0.024255 *  
## Juvenile            3.5464  1   0.059675 .  
## Prenatal:Juvenile  10.2964  1   0.001333 ** 
## ---
## Signif. codes:  0 '***' 0.001 '**' 0.01 '*' 0.05 '.' 0.1 ' ' 1
```

```
#Normality and Homocedasticity checks
e13<-resid(model13) # Pearson's residuals
pre13<-predict(model13) #predicted
shapiro.test(e13)
```

```
## 
##  Shapiro-Wilk normality test
## 
## data:  e13
## W = 0.97345, p-value = 0.8427
```

```
leveneTest(DGmol~Prenatal*Juvenile, data=data_GFAPAdult)
```

```
## Levene's Test for Homogeneity of Variance (center = median)
##       Df F value Pr(>F)
## group  3  0.9962 0.4215
##       15
```

```
par(mfrow = c(1, 2))
plot(pre13, e13, xlab="Predicted", ylab="Pearson's residuals",main="Dispersion of RE vs PRED",cex.main=.8 )
abline(0,0)
qqnorm(e13, cex.main=.8)
qqline(e13)
```

```
emmeans(model13, pairwise~Prenatal*Juvenile)
```

```
## $emmeans
##  Prenatal Juvenile emmean   SE df lower.CL upper.CL
##  VEH      LPS        27.3 1.51 14     24.1     30.6
##  VPA      LPS        22.6 1.48 13     19.4     25.8
##  VEH      SAL        23.4 1.48  2     17.1     29.8
##  VPA      SAL        28.4 1.70  2     21.1     35.7
## 
## Degrees-of-freedom method: containment 
## Confidence level used: 0.95 
## 
## $contrasts
##  contrast          estimate   SE df t.ratio p.value
##  VEH LPS - VPA LPS     4.76 2.11 13   2.253  0.1603
##  VEH LPS - VEH SAL     3.92 2.08  2   1.883  0.4379
##  VEH LPS - VPA SAL    -1.07 2.28  2  -0.472  0.9588
##  VPA LPS - VEH SAL    -0.84 2.09  2  -0.403  0.9731
##  VPA LPS - VPA SAL    -5.84 2.22  2  -2.634  0.2758
##  VEH SAL - VPA SAL    -5.00 2.25  2  -2.218  0.3539
## 
## Degrees-of-freedom method: containment 
## P value adjustment: tukey method for comparing a family of 4 estimates
```

## SUPPLEMENTARY FIGURE 2B: GFAP in the Granular cell layer of the DG at adulthood

```
#Fit mixed-effects model using dam as random factor
model14<-lme(DGgran~Prenatal*Juvenile, data=data_GFAPAdult, na.action=na.exclude, random=~1|Dam)
Anova(model14,type="III")
```

```
## Analysis of Deviance Table (Type III tests)
## 
## Response: DGgran
##                     Chisq Df Pr(>Chisq)    
## (Intercept)       40.3832  1  2.087e-10 ***
## Prenatal           0.1390  1     0.7093    
## Juvenile           0.2111  1     0.6459    
## Prenatal:Juvenile  0.3804  1     0.5374    
## ---
## Signif. codes:  0 '***' 0.001 '**' 0.01 '*' 0.05 '.' 0.1 ' ' 1
```

```
#Normality and Homocedasticity checks
e14<-resid(model14) # Pearson's residuals
pre14<-predict(model14) #predicted
shapiro.test(e14)
```

```
## 
##  Shapiro-Wilk normality test
## 
## data:  e14
## W = 0.9284, p-value = 0.1618
```

```
leveneTest(DGgran~Prenatal*Juvenile, data=data_GFAPAdult)
```

```
## Levene's Test for Homogeneity of Variance (center = median)
##       Df F value Pr(>F)
## group  3  0.0901 0.9644
##       15
```

```
par(mfrow = c(1, 2))
plot(pre14, e14, xlab="Predicted", ylab="Pearson's residuals",main="Dispersion of RE vs PRED",cex.main=.8 )
abline(0,0)
qqnorm(e14, cex.main=.8)
qqline(e14)
```

## SUPPLEMENTARY FIGURE 2C: GFAP in the Hilus of the DG at adulthood

```
#Fit mixed-effects model using dam as random factor
model15<-lme(DGhilus~Prenatal*Juvenile, data=data_GFAPAdult, na.action=na.exclude, random=~1|Dam)
Anova(model15, type="III")
```

```
## Analysis of Deviance Table (Type III tests)
## 
## Response: DGhilus
##                      Chisq Df Pr(>Chisq)    
## (Intercept)       413.2890  1     <2e-16 ***
## Prenatal            0.1056  1     0.7452    
## Juvenile            0.0001  1     0.9926    
## Prenatal:Juvenile   0.5617  1     0.4536    
## ---
## Signif. codes:  0 '***' 0.001 '**' 0.01 '*' 0.05 '.' 0.1 ' ' 1
```

```
#Normality and Homocedasticity checks
e15<-resid(model15) # Pearson's residuals
pre15<-predict(model15) #predicted
shapiro.test(e15)
```

```
## 
##  Shapiro-Wilk normality test
## 
## data:  e15
## W = 0.95387, p-value = 0.4587
```

```
leveneTest(DGhilus~Prenatal*Juvenile, data=data_GFAPAdult)
```

```
## Levene's Test for Homogeneity of Variance (center = median)
##       Df F value Pr(>F)
## group  3  0.5456 0.6586
##       15
```

```
par(mfrow = c(1, 2))
plot(pre15, e15, xlab="Predicted", ylab="Pearson's residuals",main="Dispersion of RE vs PRED",cex.main=.8 )
abline(0,0)
qqnorm(e15, cex.main=.8)
qqline(e15)
```

## SUPPLEMENTARY FIGURE 2D: IBA1 in the Molecular layer of the DG at adulthood

### Total cells

```
#Fit mixed-effects model using dam as random factor
model16<-lme(DGmol_total~Prenatal*Juvenile, data=data_IBA1Adult, na.action=na.exclude, random=~1|Dam)
Anova(model16,type="III")
```

```
## Analysis of Deviance Table (Type III tests)
## 
## Response: DGmol_total
##                      Chisq Df Pr(>Chisq)    
## (Intercept)       316.8275  1     <2e-16 ***
## Prenatal            0.1626  1     0.6868    
## Juvenile            0.1173  1     0.7320    
## Prenatal:Juvenile   0.3458  1     0.5565    
## ---
## Signif. codes:  0 '***' 0.001 '**' 0.01 '*' 0.05 '.' 0.1 ' ' 1
```

```
#Normality and Homocedasticity checks
e16<-resid(model16) # Pearson's residuals
pre16<-predict(model16) #predicted
shapiro.test(e16)
```

```
## 
##  Shapiro-Wilk normality test
## 
## data:  e16
## W = 0.97801, p-value = 0.9169
```

```
leveneTest(DGmol_total~Prenatal*Juvenile, data=data_IBA1Adult)
```

```
## Levene's Test for Homogeneity of Variance (center = median)
##       Df F value Pr(>F)
## group  3  0.7991 0.5134
##       15
```

```
par(mfrow = c(1, 2))
plot(pre16, e16, xlab="Predicted", ylab="Pearson's residuals",main="Dispersion of RE vs PRED",cex.main=.8 )
abline(0,0)
qqnorm(e16, cex.main=.8)
qqline(e16)
```

### Ramified cells

```
#Fit mixed-effects model using dam as random factor
model17<-lme(DGmol_type1~Prenatal*Juvenile, data=data_IBA1Adult, na.action=na.exclude, random=~1|Dam)
Anova(model17,type="III")
```

```
## Analysis of Deviance Table (Type III tests)
## 
## Response: DGmol_type1
##                      Chisq Df Pr(>Chisq)    
## (Intercept)       429.5696  1     <2e-16 ***
## Prenatal            0.3355  1     0.5624    
## Juvenile            0.2697  1     0.6036    
## Prenatal:Juvenile   0.0993  1     0.7527    
## ---
## Signif. codes:  0 '***' 0.001 '**' 0.01 '*' 0.05 '.' 0.1 ' ' 1
```

```
#Normality and Homocedasticity checks
e17<-resid(model17) # Pearson's residuals
pre17<-predict(model17) #predicted
shapiro.test(e17)
```

```
## 
##  Shapiro-Wilk normality test
## 
## data:  e17
## W = 0.92372, p-value = 0.1326
```

```
leveneTest(DGmol_type1~Prenatal*Juvenile, data=data_IBA1Adult)
```

```
## Levene's Test for Homogeneity of Variance (center = median)
##       Df F value Pr(>F)
## group  3  1.2531 0.3258
##       15
```

```
par(mfrow = c(1, 2))
plot(pre17, e17, xlab="Predicted", ylab="Pearson's residuals",main="Dispersion of RE vs PRED",cex.main=.8 )
abline(0,0)
qqnorm(e17, cex.main=.8)
qqline(e17)
```

### Hypertrophic cells

```
#Fit mixed-effects model using dam as random factor
model18<-lme(DGmol_type2~Prenatal*Juvenile, data=data_IBA1Adult, na.action=na.exclude, random=~1|Dam)
Anova(model18,type="III")
```

```
## Analysis of Deviance Table (Type III tests)
## 
## Response: DGmol_type2
##                    Chisq Df Pr(>Chisq)   
## (Intercept)       9.0315  1   0.002654 **
## Prenatal          0.0325  1   0.856842   
## Juvenile          0.0463  1   0.829685   
## Prenatal:Juvenile 0.8775  1   0.348896   
## ---
## Signif. codes:  0 '***' 0.001 '**' 0.01 '*' 0.05 '.' 0.1 ' ' 1
```

```
#Normality and Homocedasticity checks
e18<-resid(model18) # Pearson's residuals
pre18<-predict(model18) #predicted
shapiro.test(e18)
```

```
## 
##  Shapiro-Wilk normality test
## 
## data:  e18
## W = 0.94203, p-value = 0.2867
```

```
leveneTest(DGmol_type2~Prenatal*Juvenile, data=data_IBA1Adult)
```

```
## Levene's Test for Homogeneity of Variance (center = median)
##       Df F value Pr(>F)
## group  3  0.8385 0.4937
##       15
```

```
par(mfrow = c(1, 2))
plot(pre18, e18, xlab="Predicted", ylab="Pearson's residuals",main="Dispersion of RE vs PRED",cex.main=.8 )
abline(0,0)
qqnorm(e18, cex.main=.8)
qqline(e18)
```

## SUPPLEMENTARY FIGURE 2E: IBA1 in the Granular cell layer of the DG at adulthood

### Total cells

```
#Fit mixed-effects model using dam as random factor
model19<-lme(DGgran_total~Prenatal*Juvenile, data=data_IBA1Adult, na.action=na.exclude, random=~1|Dam)
Anova(model19,type="III")
```

```
## Analysis of Deviance Table (Type III tests)
## 
## Response: DGgran_total
##                      Chisq Df Pr(>Chisq)    
## (Intercept)       116.2819  1     <2e-16 ***
## Prenatal            0.0664  1     0.7966    
## Juvenile            0.2232  1     0.6366    
## Prenatal:Juvenile   1.3688  1     0.2420    
## ---
## Signif. codes:  0 '***' 0.001 '**' 0.01 '*' 0.05 '.' 0.1 ' ' 1
```

```
#Normality and Homocedasticity checks
e19<-resid(model19) # Pearson's residuals
pre19<-predict(model19) #predicted
shapiro.test(e19)
```

```
## 
##  Shapiro-Wilk normality test
## 
## data:  e19
## W = 0.95362, p-value = 0.4543
```

```
leveneTest(DGgran_total~Prenatal*Juvenile, data=data_IBA1Adult)
```

```
## Levene's Test for Homogeneity of Variance (center = median)
##       Df F value Pr(>F)
## group  3  0.2531 0.8579
##       15
```

```
par(mfrow = c(1, 2))
plot(pre19, e19, xlab="Predicted", ylab="Pearson's residuals",main="Dispersion of RE vs PRED",cex.main=.8 )
abline(0,0)
qqnorm(e19, cex.main=.8)
qqline(e19)
```

### Ramified cells

```
#Fit mixed-effects model using dam as random factor
model20<-lme(DGgran_type1~Prenatal*Juvenile, data=data_IBA1Adult, na.action=na.exclude, random=~1|Dam)
Anova(model20,type="III")
```

```
## Analysis of Deviance Table (Type III tests)
## 
## Response: DGgran_type1
##                     Chisq Df Pr(>Chisq)    
## (Intercept)       87.7895  1     <2e-16 ***
## Prenatal           0.1045  1     0.7465    
## Juvenile           0.1709  1     0.6793    
## Prenatal:Juvenile  0.6777  1     0.4104    
## ---
## Signif. codes:  0 '***' 0.001 '**' 0.01 '*' 0.05 '.' 0.1 ' ' 1
```

```
#Normality and Homocedasticity checks
e20<-resid(model20) # Pearson's residuals
pre20<-predict(model20) #predicted
shapiro.test(e20)
```

```
## 
##  Shapiro-Wilk normality test
## 
## data:  e20
## W = 0.95354, p-value = 0.4529
```

```
leveneTest(DGgran_type1~Prenatal*Juvenile, data=data_IBA1Adult)
```

```
## Levene's Test for Homogeneity of Variance (center = median)
##       Df F value Pr(>F)
## group  3  0.3339 0.8011
##       15
```

```
par(mfrow = c(1, 2))
plot(pre20, e20, xlab="Predicted", ylab="Pearson's residuals",main="Dispersion of RE vs PRED",cex.main=.8 )
abline(0,0)
qqnorm(e20, cex.main=.8)
qqline(e20)
```

### Hypertrophic

```
#Fit mixed-effects model using dam as random factor
model21<-lme(DGgran_type2~Prenatal*Juvenile, data=data_IBA1Adult, na.action=na.exclude, random=~1|Dam)
Anova(model21,type="III")
```

```
## Analysis of Deviance Table (Type III tests)
## 
## Response: DGgran_type2
##                     Chisq Df Pr(>Chisq)   
## (Intercept)       10.1381  1   0.001452 **
## Prenatal           0.0851  1   0.770485   
## Juvenile           0.1667  1   0.683029   
## Prenatal:Juvenile  0.9228  1   0.336735   
## ---
## Signif. codes:  0 '***' 0.001 '**' 0.01 '*' 0.05 '.' 0.1 ' ' 1
```

```
#Normality and Homocedasticity checks
e21<-resid(model21) # Pearson's residuals
pre21<-predict(model21) #predicted
shapiro.test(e21)
```

```
## 
##  Shapiro-Wilk normality test
## 
## data:  e21
## W = 0.97332, p-value = 0.8404
```

```
leveneTest(DGgran_type2~Prenatal*Juvenile, data=data_IBA1Adult)
```

```
## Levene's Test for Homogeneity of Variance (center = median)
##       Df F value Pr(>F)
## group  3  0.7623 0.5326
##       15
```

```
par(mfrow = c(1, 2))
plot(pre21, e21, xlab="Predicted", ylab="Pearson's residuals",main="Dispersion of RE vs PRED",cex.main=.8 )
abline(0,0)
qqnorm(e21, cex.main=.8)
qqline(e21)
```

## SUPPLEMENTARY FIGURE 2F: IBA1 in the Hilus of the DG at adulthood

### Total cells

```
#Fit mixed-effects model using dam as random factor
model22<-lme(DGhilus_total~Prenatal*Juvenile, data=data_IBA1Adult, na.action=na.exclude, random=~1|Dam)
Anova(model22,type="III")
```

```
## Analysis of Deviance Table (Type III tests)
## 
## Response: DGhilus_total
##                     Chisq Df Pr(>Chisq)    
## (Intercept)       94.7511  1     <2e-16 ***
## Prenatal           0.9788  1     0.3225    
## Juvenile           0.9028  1     0.3420    
## Prenatal:Juvenile  0.7652  1     0.3817    
## ---
## Signif. codes:  0 '***' 0.001 '**' 0.01 '*' 0.05 '.' 0.1 ' ' 1
```

```
#Normality and Homocedasticity checks
e22<-resid(model22) # Pearson's residuals
pre22<-predict(model22) #predicted
shapiro.test(e22)
```

```
## 
##  Shapiro-Wilk normality test
## 
## data:  e22
## W = 0.92623, p-value = 0.1475
```

```
leveneTest(DGhilus_total~Prenatal*Juvenile, data=data_IBA1Adult)
```

```
## Levene's Test for Homogeneity of Variance (center = median)
##       Df F value Pr(>F)
## group  3  0.1563  0.924
##       15
```

```
par(mfrow = c(1, 2))
plot(pre22, e22, xlab="Predicted", ylab="Pearson's residuals",main="Dispersion of RE vs PRED",cex.main=.8 )
abline(0,0)
qqnorm(e22, cex.main=.8)
qqline(e22)
```

### Ramified cells

```
#Fit mixed-effects model using dam as random factor
model23<-lme(DGhilus_type1~Prenatal*Juvenile, data=data_IBA1Adult, na.action=na.exclude, random=~1|Dam)
Anova(model23,type="III")
```

```
## Analysis of Deviance Table (Type III tests)
## 
## Response: DGhilus_type1
##                     Chisq Df Pr(>Chisq)    
## (Intercept)       83.6641  1     <2e-16 ***
## Prenatal           0.8803  1     0.3481    
## Juvenile           0.6581  1     0.4172    
## Prenatal:Juvenile  0.5635  1     0.4529    
## ---
## Signif. codes:  0 '***' 0.001 '**' 0.01 '*' 0.05 '.' 0.1 ' ' 1
```

```
#Normality and Homocedasticity checks
e23<-resid(model23) # Pearson's residuals
pre23<-predict(model23) #predicted
shapiro.test(e23)
```

```
## 
##  Shapiro-Wilk normality test
## 
## data:  e23
## W = 0.93059, p-value = 0.1776
```

```
leveneTest(DGhilus_type1~Prenatal*Juvenile, data=data_IBA1Adult)
```

```
## Levene's Test for Homogeneity of Variance (center = median)
##       Df F value Pr(>F)
## group  3  0.2604 0.8528
##       15
```

```
par(mfrow = c(1, 2))
plot(pre23, e23, xlab="Predicted", ylab="Pearson's residuals",main="Dispersion of RE vs PRED",cex.main=.8 )
abline(0,0)
qqnorm(e23, cex.main=.8)
qqline(e23)
```

### Hypertrophic

```
#Fit mixed-effects model using dam as random factor
model24<-lme(DGhilus_type2~Prenatal*Juvenile, data=data_IBA1Adult, na.action=na.exclude, random=~1|Dam)
Anova(model24,type="III")
```

```
## Analysis of Deviance Table (Type III tests)
## 
## Response: DGhilus_type2
##                     Chisq Df Pr(>Chisq)    
## (Intercept)       44.8969  1  2.077e-11 ***
## Prenatal           0.5015  1     0.4788    
## Juvenile           1.4615  1     0.2267    
## Prenatal:Juvenile  0.9532  1     0.3289    
## ---
## Signif. codes:  0 '***' 0.001 '**' 0.01 '*' 0.05 '.' 0.1 ' ' 1
```

```
#Normality and Homocedasticity checks
e24<-resid(model24) # Pearson's residuals
pre24<-predict(model24) #predicted
shapiro.test(e24)
```

```
## 
##  Shapiro-Wilk normality test
## 
## data:  e24
## W = 0.97993, p-value = 0.9415
```

```
leveneTest(DGhilus_type2~Prenatal*Juvenile, data=data_IBA1Adult)
```

```
## Levene's Test for Homogeneity of Variance (center = median)
##       Df F value Pr(>F)
## group  3  0.1785 0.9093
##       15
```

```
par(mfrow = c(1, 2))
plot(pre24, e24, xlab="Predicted", ylab="Pearson's residuals",main="Dispersion of RE vs PRED",cex.main=.8 )
abline(0,0)
qqnorm(e24, cex.main=.8)
qqline(e24)
```

# SUPPLEMENTARY FIGURE 3: MICROGLIA PARAMETERS IN THE ADULT CEREBELLUM

```
data_ShollCerMol_Av <- read_excel("VPAxLPS_Data.xlsx", sheet = "Sholl_CerMol_Ad_Av")
data_ShollCerMol_Av[data_ShollCerMol_Av==""]<-NA
data_ShollCerGran_Av <- read_excel("VPAxLPS_Data.xlsx", sheet = "Sholl_CerGran_Ad_Av")
data_ShollCerGran_Av[data_ShollCerGran_Av==""]<-NA
```

## SUPPLEMENTARY FIGURE 3A: CELL AREA IN THE MOLECULAR LAYER

```
#Fit mixed-effects model using Dam as random factor.
model25<-lme(CerMol_Area~Prenatal*Juvenile, data=data_ShollCerMol_Av, na.action=na.exclude, random=~1|Dam)
Anova(model25, type="III")
```

```
## Analysis of Deviance Table (Type III tests)
## 
## Response: CerMol_Area
##                     Chisq Df Pr(>Chisq)    
## (Intercept)       89.9747  1  < 2.2e-16 ***
## Prenatal           0.6321  1     0.4266    
## Juvenile          17.5323  1  2.825e-05 ***
## Prenatal:Juvenile  0.5468  1     0.4596    
## ---
## Signif. codes:  0 '***' 0.001 '**' 0.01 '*' 0.05 '.' 0.1 ' ' 1
```

```
#Normality and Homocedasticity checks
e25<-resid(model25) # Pearson's residuals
pre25<-predict(model25) #predicted
shapiro.test(e25)
```

```
## 
##  Shapiro-Wilk normality test
## 
## data:  e25
## W = 0.96275, p-value = 0.6555
```

```
leveneTest(CerMol_Area~Prenatal*Juvenile, data=data_ShollCerMol_Av)
```

```
## Levene's Test for Homogeneity of Variance (center = median)
##       Df F value Pr(>F)
## group  3    1.29 0.3165
##       14
```

```
par(mfrow = c(1, 2))
plot(pre25, e25, xlab="Predicted", ylab="Pearson's residuals",main="Dispersion of RE vs PRED",cex.main=.8 )
abline(0,0)
qqnorm(e25, cex.main=.8)
qqline(e25)
```

## SUPPLEMENTARY FIGURE 3B: CELL AREA IN THE GRANULAR CELL LAYER

```
#Fit mixed-effects model using Dam as random factor.
model26<-lme(CerGran_Area~Prenatal*Juvenile, data=data_ShollCerGran_Av, na.action=na.exclude, random=~1|Dam)
Anova(model26, type="III")
```

```
## Analysis of Deviance Table (Type III tests)
## 
## Response: CerGran_Area
##                     Chisq Df Pr(>Chisq)    
## (Intercept)       39.3389  1  3.563e-10 ***
## Prenatal           1.0177  1     0.3131    
## Juvenile           0.0080  1     0.9286    
## Prenatal:Juvenile  1.1743  1     0.2785    
## ---
## Signif. codes:  0 '***' 0.001 '**' 0.01 '*' 0.05 '.' 0.1 ' ' 1
```

```
#Normality and Homocedasticity checks
e26<-resid(model26) # Pearson's residuals
pre26<-predict(model26) #predicted
shapiro.test(e26)
```

```
## 
##  Shapiro-Wilk normality test
## 
## data:  e26
## W = 0.96782, p-value = 0.7558
```

```
leveneTest(CerGran_Area~Prenatal*Juvenile, data=data_ShollCerGran_Av)
```

```
## Levene's Test for Homogeneity of Variance (center = median)
##       Df F value Pr(>F)
## group  3  1.2283 0.3363
##       14
```

```
par(mfrow = c(1, 2))
plot(pre26, e26, xlab="Predicted", ylab="Pearson's residuals",main="Dispersion of RE vs PRED",cex.main=.8 )
abline(0,0)
qqnorm(e26, cex.main=.8)
qqline(e26)
```

## SUPPLEMENTARY FIGURE 3C: MICROGLIAL SOMA SPHERICITY IN THE MOLECULAR LAYER

```
#Fit mixed-effects model using Dam as random factor.
model27<-lme(CerMol_Sphericity~Prenatal*Juvenile, data=data_ShollCerMol_Av, na.action=na.exclude, random=~1|Dam)
Anova(model27,type="III")
```

```
## Analysis of Deviance Table (Type III tests)
## 
## Response: CerMol_Sphericity
##                      Chisq Df Pr(>Chisq)    
## (Intercept)       223.8346  1     <2e-16 ***
## Prenatal            0.0830  1     0.7732    
## Juvenile            0.4150  1     0.5195    
## Prenatal:Juvenile   0.5882  1     0.4431    
## ---
## Signif. codes:  0 '***' 0.001 '**' 0.01 '*' 0.05 '.' 0.1 ' ' 1
```

```
#Normality and Homocedasticity checks
e27<-resid(model27) # Pearson's residuals
pre27<-predict(model27) #predicted
shapiro.test(e27)
```

```
## 
##  Shapiro-Wilk normality test
## 
## data:  e27
## W = 0.96079, p-value = 0.617
```

```
leveneTest(log(CerMol_Sphericity)~Prenatal*Juvenile, data=data_ShollCerMol_Av)
```

```
## Levene's Test for Homogeneity of Variance (center = median)
##       Df F value Pr(>F)
## group  3  0.4038 0.7526
##       14
```

```
par(mfrow = c(1, 2))
plot(pre27, e27, xlab="Predicted", ylab="Pearson's residuals",main="Dispersion of RE vs PRED",cex.main=.8 )
abline(0,0)
qqnorm(e27, cex.main=.8)
qqline(e27)
```

## SUPPLEMENTARY FIGURE 3D: MICROGLIAL SOMA SPHERICITY IN THE GRANULAR CELL LAYER

```
#Fit mixed-effects model using Dam as random factor.
model28<-lme(CerGran_Sphericity~Prenatal*Juvenile, data=data_ShollCerGran_Av, na.action=na.exclude, random=~1|Dam)
Anova(model28,type="III")
```

```
## Analysis of Deviance Table (Type III tests)
## 
## Response: CerGran_Sphericity
##                      Chisq Df Pr(>Chisq)    
## (Intercept)       462.7267  1     <2e-16 ***
## Prenatal            1.1499  1     0.2836    
## Juvenile            0.0140  1     0.9058    
## Prenatal:Juvenile   0.1571  1     0.6918    
## ---
## Signif. codes:  0 '***' 0.001 '**' 0.01 '*' 0.05 '.' 0.1 ' ' 1
```

```
#Normality and Homocedasticity checks
e28<-resid(model28) # Pearson's residuals
pre28<-predict(model28) #predicted
shapiro.test(e28)
```

```
## 
##  Shapiro-Wilk normality test
## 
## data:  e28
## W = 0.9658, p-value = 0.716
```

```
leveneTest(CerGran_Sphericity~Prenatal*Juvenile, data=data_ShollCerGran_Av)
```

```
## Levene's Test for Homogeneity of Variance (center = median)
##       Df F value Pr(>F)
## group  3  0.5763   0.64
##       14
```

```
par(mfrow = c(1, 2))
plot(pre28, e28, xlab="Predicted", ylab="Pearson's residuals",main="Dispersion of RE vs PRED",cex.main=.8 )
abline(0,0)
qqnorm(e28, cex.main=.8)
qqline(e28)
```

## SUPPLEMENTARY FIGURE 3E: MICROGLIAL CELL MAXIMAL RADIO IN THE MOLECULAR LAYER

```
#Fit mixed-effects model using Dam as random factor.
model29<-lme(CerMol_Max_radio~Prenatal*Juvenile, data=data_ShollCerMol_Av, na.action=na.exclude, random=~1|Dam)
Anova(model29, type="III")
```

```
## Analysis of Deviance Table (Type III tests)
## 
## Response: CerMol_Max_radio
##                      Chisq Df Pr(>Chisq)    
## (Intercept)       276.5386  1     <2e-16 ***
## Prenatal            0.0679  1     0.7945    
## Juvenile            1.5691  1     0.2103    
## Prenatal:Juvenile   0.0024  1     0.9610    
## ---
## Signif. codes:  0 '***' 0.001 '**' 0.01 '*' 0.05 '.' 0.1 ' ' 1
```

```
#Normality and Homocedasticity checks
e29<-resid(model29) # Pearson's residuals
pre29<-predict(model29) #predicted
shapiro.test(e29)
```

```
## 
##  Shapiro-Wilk normality test
## 
## data:  e29
## W = 0.95614, p-value = 0.5292
```

```
leveneTest(CerMol_Max_radio~Prenatal*Juvenile, data=data_ShollCerMol_Av)
```

```
## Levene's Test for Homogeneity of Variance (center = median)
##       Df F value Pr(>F)
## group  3  1.1119 0.3774
##       14
```

```
par(mfrow = c(1, 2))
plot(pre29, e29, xlab="Predicted", ylab="Pearson's residuals",main="Dispersion of RE vs PRED",cex.main=.8 )
abline(0,0)
qqnorm(e29, cex.main=.8)
qqline(e29)
```

## SUPPLEMENTARY FIGURE 3F: MICROGLIAL CELL MAXIMAL RADIO IN THE GRANULAR CELL LAYER

```
#Fit mixed-effects model using Dam as random factor.
model30<-lme(CerGran_Max_radio~Prenatal*Juvenile, data=data_ShollCerGran_Av, na.action=na.exclude, random=~1|Dam)
Anova(model30,type="III")
```

```
## Analysis of Deviance Table (Type III tests)
## 
## Response: CerGran_Max_radio
##                      Chisq Df Pr(>Chisq)    
## (Intercept)       246.1694  1    < 2e-16 ***
## Prenatal            0.0514  1    0.82058    
## Juvenile            3.9474  1    0.04694 *  
## Prenatal:Juvenile   2.4241  1    0.11948    
## ---
## Signif. codes:  0 '***' 0.001 '**' 0.01 '*' 0.05 '.' 0.1 ' ' 1
```

```
#Normality and Homocedasticity checks
e30<-resid(model30) # Pearson's residuals
pre30<-predict(model30) #predicted
shapiro.test(e30)
```

```
## 
##  Shapiro-Wilk normality test
## 
## data:  e30
## W = 0.96318, p-value = 0.664
```

```
leveneTest(CerGran_Max_radio~Prenatal*Juvenile, data=data_ShollCerGran_Av)
```

```
## Levene's Test for Homogeneity of Variance (center = median)
##       Df F value Pr(>F)
## group  3  0.2106 0.8874
##       14
```

```
par(mfrow = c(1, 2))
plot(pre30, e30, xlab="Predicted", ylab="Pearson's residuals",main="Dispersion of RE vs PRED",cex.main=.8 )
abline(0,0)
qqnorm(e30, cex.main=.8)
qqline(e30)
```

# SUPPLEMENTARY FIGURE 4: NEUROINFLAMMATION IN THE HIPPOCAMPAL CA1 AT PD22 AND PD36

# GFAP AT PD22

```
data_GFAPpd22 <- read_excel("VPAxLPS_Data.xlsx", sheet = "GFAP_PD22")
#my_data <- read_excel("my_file.xlsx", sheet = "data")
data_GFAPpd22[data_GFAPpd22==""]<-NA
```

## SUPPLEMENTARY FIGURE 4A: GFAP in CA1 stratum oriens at PD22

```
#Fit mixed-effects model using dam as random factor
model31<-lme(CA1or~Prenatal*PD22, data=data_GFAPpd22, na.action=na.exclude, random=~1|Dam)
Anova(model31, type="III")
```

```
## Analysis of Deviance Table (Type III tests)
## 
## Response: CA1or
##                  Chisq Df Pr(>Chisq)    
## (Intercept)   136.2414  1    < 2e-16 ***
## Prenatal        1.2507  1    0.26343    
## PD22            3.4363  1    0.06378 .  
## Prenatal:PD22   2.1940  1    0.13855    
## ---
## Signif. codes:  0 '***' 0.001 '**' 0.01 '*' 0.05 '.' 0.1 ' ' 1
```

```
#Normality and Homocedasticity checks
e31<-resid(model31) # Pearson's residuals
pre31<-predict(model31) #predicted
shapiro.test(e31)
```

```
## 
##  Shapiro-Wilk normality test
## 
## data:  e31
## W = 0.94892, p-value = 0.5075
```

```
leveneTest(CA1or~Prenatal*PD22, data=data_GFAPpd22)
```

```
## Levene's Test for Homogeneity of Variance (center = median)
##       Df F value Pr(>F)
## group  3  0.6324 0.6093
##       11
```

```
par(mfrow = c(1, 2))
plot(pre31, e31, xlab="Predicted", ylab="Pearson's residuals",main="Dispersion of RE vs PRED",cex.main=.8 )
abline(0,0)
qqnorm(e31, cex.main=.8)
qqline(e31)
```

## SUPPLEMENTARY FIGURE 4B: GFAP in CA1 pyramidal cell layer at PD22

```
#Fit mixed-effects model using dam as random factor
model32<-lme(CA1pyr~Prenatal*PD22, data=data_GFAPpd22, na.action=na.exclude, random=~1|Dam)
Anova(model32,type="III")
```

```
## Analysis of Deviance Table (Type III tests)
## 
## Response: CA1pyr
##                Chisq Df Pr(>Chisq)   
## (Intercept)   8.2442  1   0.004088 **
## Prenatal      0.0873  1   0.767632   
## PD22          0.6029  1   0.437482   
## Prenatal:PD22 0.0125  1   0.911083   
## ---
## Signif. codes:  0 '***' 0.001 '**' 0.01 '*' 0.05 '.' 0.1 ' ' 1
```

```
#Normality and Homocedasticity checks
e32<-resid(model32) # Pearson's residuals
pre32<-predict(model32) #predicted
shapiro.test(e32)
```

```
## 
##  Shapiro-Wilk normality test
## 
## data:  e32
## W = 0.9617, p-value = 0.7219
```

```
leveneTest(CA1pyr~Prenatal*PD22, data=data_GFAPpd22)
```

```
## Levene's Test for Homogeneity of Variance (center = median)
##       Df F value Pr(>F)
## group  3  0.5932 0.6323
##       11
```

```
par(mfrow = c(1, 2))
plot(pre32, e32, xlab="Predicted", ylab="Pearson's residuals",main="Dispersion of RE vs PRED",cex.main=.8 )
abline(0,0)
qqnorm(e32, cex.main=.8)
qqline(e32)
```

## SUPPLEMENTARY FIGURE 4C: GFAP in CA1 stratum radiatum at PD22

```
#Fit mixed-effects model using dam as random factor
model33<-lme(CA1rad~Prenatal*PD22, data=data_GFAPpd22, na.action=na.exclude, random=~1|Dam)
Anova(model33, type="III")
```

```
## Analysis of Deviance Table (Type III tests)
## 
## Response: CA1rad
##                 Chisq Df Pr(>Chisq)    
## (Intercept)   58.0192  1  2.596e-14 ***
## Prenatal       0.2687  1  0.6042085    
## PD22          12.6173  1  0.0003822 ***
## Prenatal:PD22  0.3916  1  0.5314376    
## ---
## Signif. codes:  0 '***' 0.001 '**' 0.01 '*' 0.05 '.' 0.1 ' ' 1
```

```
#Normality and Homocedasticity checks
e33<-resid(model33) # Pearson's residuals
pre33<-predict(model33) #predicted
shapiro.test(e33)
```

```
## 
##  Shapiro-Wilk normality test
## 
## data:  e33
## W = 0.96964, p-value = 0.8526
```

```
leveneTest(CA1rad~Prenatal*PD22, data=data_GFAPpd22)
```

```
## Levene's Test for Homogeneity of Variance (center = median)
##       Df F value Pr(>F)
## group  3  0.8377  0.501
##       11
```

```
par(mfrow = c(1, 2))
plot(pre33, e33, xlab="Predicted", ylab="Pearson's residuals",main="Dispersion of RE vs PRED",cex.main=.8 )
abline(0,0)
qqnorm(e33, cex.main=.8)
qqline(e33)
```

# IBA1 AT PD22

```
data_IBA1pd22 <- read_excel("VPAxLPS_Data.xlsx", sheet = "IBA1_PD22")
#my_data <- read_excel("my_file.xlsx", sheet = "data")
data_IBA1pd22[data_IBA1pd22==""]<-NA
```

## SUPPLEMENTARY FIGURE 4D: IBA1 in hippocampal CA1 stratum oriens at PD22

### Total cells

```
#Fit mixed-effects model using dam as random factor
model34<-lme(CA1or_total~Prenatal*PD22, data=data_IBA1pd22, na.action=na.exclude, random=~1|Dam)
Anova(model34,type="III")
```

```
## Analysis of Deviance Table (Type III tests)
## 
## Response: CA1or_total
##                 Chisq Df Pr(>Chisq)    
## (Intercept)   49.1994  1  2.312e-12 ***
## Prenatal       0.0060  1     0.9380    
## PD22           0.0685  1     0.7936    
## Prenatal:PD22  0.6958  1     0.4042    
## ---
## Signif. codes:  0 '***' 0.001 '**' 0.01 '*' 0.05 '.' 0.1 ' ' 1
```

```
#Normality and Homocedasticity checks
e34<-resid(model34) # Pearson's residuals
pre34<-predict(model34) #predicted
shapiro.test(e34)
```

```
## 
##  Shapiro-Wilk normality test
## 
## data:  e34
## W = 0.96688, p-value = 0.7374
```

```
leveneTest(CA1or_total~Prenatal*PD22, data=data_IBA1pd22)
```

```
## Levene's Test for Homogeneity of Variance (center = median)
##       Df F value Pr(>F)
## group  3  0.1016 0.9577
##       14
```

```
par(mfrow = c(1, 2))
plot(pre34, e34, xlab="Predicted", ylab="Pearson's residuals",main="Dispersion of RE vs PRED",cex.main=.8 )
abline(0,0)
qqnorm(e34, cex.main=.8)
qqline(e34)
```

### Ramified cells

```
#Fit mixed-effects model using dam as random factor
model35<-lme(CA1or_type1~Prenatal*PD22, data=data_IBA1pd22, na.action=na.exclude, random=~1|Dam)
Anova(model35,type="III")
```

```
## Analysis of Deviance Table (Type III tests)
## 
## Response: CA1or_type1
##                 Chisq Df Pr(>Chisq)    
## (Intercept)   66.3896  1    3.7e-16 ***
## Prenatal       0.1199  1     0.7292    
## PD22           0.0361  1     0.8494    
## Prenatal:PD22  0.3573  1     0.5500    
## ---
## Signif. codes:  0 '***' 0.001 '**' 0.01 '*' 0.05 '.' 0.1 ' ' 1
```

```
#Normality and Homocedasticity checks
e35<-resid(model35) # Pearson's residuals
pre35<-predict(model35) #predicted
shapiro.test(e35)
```

```
## 
##  Shapiro-Wilk normality test
## 
## data:  e35
## W = 0.94061, p-value = 0.2968
```

```
leveneTest(CA1or_type1~Prenatal*PD22, data=data_IBA1pd22)
```

```
## Levene's Test for Homogeneity of Variance (center = median)
##       Df F value Pr(>F)
## group  3  0.1212 0.9461
##       14
```

```
par(mfrow = c(1, 2))
plot(pre35, e35, xlab="Predicted", ylab="Pearson's residuals",main="Dispersion of RE vs PRED",cex.main=.8 )
abline(0,0)
qqnorm(e35, cex.main=.8)
qqline(e35)
```

### Hypertrophic cells

```
#Fit linear model using generalized least squares and ANOVA
model36<-lme(CA1or_type2~Prenatal*PD22, data=data_IBA1pd22, na.action=na.exclude, random=~1|Dam)
Anova(model36,type="III")
```

```
## Analysis of Deviance Table (Type III tests)
## 
## Response: CA1or_type2
##                Chisq Df Pr(>Chisq)
## (Intercept)   0.4349  1     0.5096
## Prenatal      1.2903  1     0.2560
## PD22          1.8953  1     0.1686
## Prenatal:PD22 0.8247  1     0.3638
```

```
#Normality and Homocedasticity checks
e36<-resid(model36) # Pearson's residuals
pre36<-predict(model36) #predicted
shapiro.test(e36)
```

```
## 
##  Shapiro-Wilk normality test
## 
## data:  e36
## W = 0.93407, p-value = 0.2288
```

```
leveneTest(CA1or_type2~Prenatal*PD22, data=data_IBA1pd22)
```

```
## Levene's Test for Homogeneity of Variance (center = median)
##       Df F value Pr(>F)
## group  3  1.4484 0.2711
##       14
```

```
par(mfrow = c(1, 2))
plot(pre36, e36, xlab="Predicted", ylab="Pearson's residuals",main="Dispersion of RE vs PRED",cex.main=.8 )
abline(0,0)
qqnorm(e36, cex.main=.8)
qqline(e36)
```

## SUPPLEMENTARY FIGURE 4E: IBA1 in hippocampal CA1 pyramidal cell layer at PD22

### Total cells

```
#Fit mixed-effects model using dam as random factor
model37<-lme(CA1pyr_total~Prenatal*PD22, data=data_IBA1pd22, na.action=na.exclude, random=~1|Dam)
Anova(model37,type="III")
```

```
## Analysis of Deviance Table (Type III tests)
## 
## Response: CA1pyr_total
##                 Chisq Df Pr(>Chisq)    
## (Intercept)   45.1302  1  1.844e-11 ***
## Prenatal       4.2143  1    0.04008 *  
## PD22           3.9198  1    0.04772 *  
## Prenatal:PD22  4.4096  1    0.03574 *  
## ---
## Signif. codes:  0 '***' 0.001 '**' 0.01 '*' 0.05 '.' 0.1 ' ' 1
```

```
#Normality and Homocedasticity checks
e37<-resid(model37) # Pearson's residuals
pre37<-predict(model37) #predicted
shapiro.test(e37)
```

```
## 
##  Shapiro-Wilk normality test
## 
## data:  e37
## W = 0.93175, p-value = 0.2084
```

```
leveneTest(log(CA1pyr_total)~Prenatal*PD22, data=data_IBA1pd22)
```

```
## Levene's Test for Homogeneity of Variance (center = median)
##       Df F value Pr(>F)
## group  3  0.1627 0.9197
##       14
```

```
par(mfrow = c(1, 2))
plot(pre37, e37, xlab="Predicted", ylab="Pearson's residuals",main="Dispersion of RE vs PRED",cex.main=.8 )
abline(0,0)
qqnorm(e37, cex.main=.8)
qqline(e37)
```

```
#Posthoc comparisons
emmeans(model37, pairwise~Prenatal*PD22)
```

```
## $emmeans
##  Prenatal PD22 emmean   SE df lower.CL upper.CL
##  VEH      LPS   13.80 2.05 11     9.28     18.3
##  VPA      LPS    7.84 2.05 10     3.26     12.4
##  VEH      SAL    8.42 1.84  4     3.31     13.5
##  VPA      SAL   10.52 1.86  4     5.36     15.7
## 
## Degrees-of-freedom method: containment 
## Confidence level used: 0.95 
## 
## $contrasts
##  contrast          estimate   SE df t.ratio p.value
##  VEH LPS - VPA LPS    5.965 2.91 10   2.053  0.2327
##  VEH LPS - VEH SAL    5.388 2.72  4   1.980  0.3282
##  VEH LPS - VPA SAL    3.279 2.77  4   1.184  0.6664
##  VPA LPS - VEH SAL   -0.577 2.76  4  -0.209  0.9962
##  VPA LPS - VPA SAL   -2.686 2.72  4  -0.989  0.7640
##  VEH SAL - VPA SAL   -2.109 2.61  4  -0.807  0.8488
## 
## Degrees-of-freedom method: containment 
## P value adjustment: tukey method for comparing a family of 4 estimates
```

### Ramified cells

```
#Fit mixed-effects model using dam as random factor
model38<-lme(CA1pyr_type1~Prenatal*PD22, data=data_IBA1pd22, na.action=na.exclude, random=~1|Dam)
Anova(model38,type="III")
```

```
## Analysis of Deviance Table (Type III tests)
## 
## Response: CA1pyr_type1
##                 Chisq Df Pr(>Chisq)    
## (Intercept)   53.9745  1  2.031e-13 ***
## Prenatal       4.2628  1    0.03896 *  
## PD22           4.0810  1    0.04337 *  
## Prenatal:PD22  5.6097  1    0.01786 *  
## ---
## Signif. codes:  0 '***' 0.001 '**' 0.01 '*' 0.05 '.' 0.1 ' ' 1
```

```
#Normality and Homocedasticity checks
e38<-resid(model38) # Pearson's residuals
pre38<-predict(model38) #predicted
shapiro.test(e38)
```

```
## 
##  Shapiro-Wilk normality test
## 
## data:  e38
## W = 0.90529, p-value = 0.07108
```

```
leveneTest(CA1pyr_type1~Prenatal*PD22, data=data_IBA1pd22)
```

```
## Levene's Test for Homogeneity of Variance (center = median)
##       Df F value Pr(>F)
## group  3   0.019 0.9963
##       14
```

```
par(mfrow = c(1, 2))
plot(pre38, e38, xlab="Predicted", ylab="Pearson's residuals",main="Dispersion of RE vs PRED",cex.main=.8 )
abline(0,0)
qqnorm(e38, cex.main=.8)
qqline(e38)
```

```
#Posthoc comparisons
emmeans(model38, pairwise~Prenatal*PD22)
```

```
## $emmeans
##  Prenatal PD22 emmean   SE df lower.CL upper.CL
##  VEH      LPS   11.95 1.63 11     8.37     15.5
##  VPA      LPS    7.20 1.63 10     3.57     10.8
##  VEH      SAL    7.77 1.46  4     3.72     11.8
##  VPA      SAL    9.90 1.52  4     5.68     14.1
## 
## Degrees-of-freedom method: containment 
## Confidence level used: 0.95 
## 
## $contrasts
##  contrast          estimate   SE df t.ratio p.value
##  VEH LPS - VPA LPS    4.750 2.30 10   2.065  0.2289
##  VEH LPS - VEH SAL    4.176 2.07  4   2.020  0.3157
##  VEH LPS - VPA SAL    2.050 2.22  4   0.922  0.7965
##  VPA LPS - VEH SAL   -0.574 2.19  4  -0.262  0.9927
##  VPA LPS - VPA SAL   -2.700 2.04  4  -1.325  0.5961
##  VEH SAL - VPA SAL   -2.127 2.10  4  -1.011  0.7533
## 
## Degrees-of-freedom method: containment 
## P value adjustment: tukey method for comparing a family of 4 estimates
```

### Hypertrophic cells

```
#Fit mixed-effects model using dam as random factor
model39<-lme(CA1pyr_type2~Prenatal*PD22, data=data_IBA1pd22, na.action=na.exclude, random=~1|Dam)
Anova(model39,type="III")
```

```
## Analysis of Deviance Table (Type III tests)
## 
## Response: CA1pyr_type2
##                Chisq Df Pr(>Chisq)  
## (Intercept)   6.1250  1    0.01333 *
## Prenatal      0.9677  1    0.32526  
## PD22          0.7918  1    0.37356  
## Prenatal:PD22 0.2896  1    0.59045  
## ---
## Signif. codes:  0 '***' 0.001 '**' 0.01 '*' 0.05 '.' 0.1 ' ' 1
```

```
#Normality and Homocedasticity checks
e39<-resid(model39) # Pearson's residuals
pre39<-predict(model39) #predicted
shapiro.test(e39)
```

```
## 
##  Shapiro-Wilk normality test
## 
## data:  e39
## W = 0.91815, p-value = 0.1199
```

```
leveneTest(CA1pyr_type2~Prenatal*PD22, data=data_IBA1pd22)
```

```
## Levene's Test for Homogeneity of Variance (center = median)
##       Df F value Pr(>F)
## group  3  1.2616 0.3255
##       14
```

```
par(mfrow = c(1, 2))
plot(pre39, e39, xlab="Predicted", ylab="Pearson's residuals",main="Dispersion of RE vs PRED",cex.main=.8 )
abline(0,0)
qqnorm(e39, cex.main=.8)
qqline(e39)
```

## SUPPLEMENTARY FIGURE 4F: IBA1 in hippocampal CA1 stratum radiatum at PD22

### Total cells

```
#Fit mixed-effects model using dam as random factor
model40<-lme(CA1rad_total~Prenatal*PD22, data=data_IBA1pd22, na.action=na.exclude, random=~1|Dam)
Anova(model40,type="III")
```

```
## Analysis of Deviance Table (Type III tests)
## 
## Response: CA1rad_total
##                 Chisq Df Pr(>Chisq)    
## (Intercept)   86.7847  1     <2e-16 ***
## Prenatal       1.5403  1     0.2146    
## PD22           2.6409  1     0.1041    
## Prenatal:PD22  1.5630  1     0.2112    
## ---
## Signif. codes:  0 '***' 0.001 '**' 0.01 '*' 0.05 '.' 0.1 ' ' 1
```

```
#Normality and Homocedasticity checks
e40<-resid(model40) # Pearson's residuals
pre40<-predict(model40) #predicted
shapiro.test(e40)
```

```
## 
##  Shapiro-Wilk normality test
## 
## data:  e40
## W = 0.95175, p-value = 0.4531
```

```
leveneTest(CA1rad_total~Prenatal*PD22, data=data_IBA1pd22)
```

```
## Levene's Test for Homogeneity of Variance (center = median)
##       Df F value Pr(>F)
## group  3  0.2713 0.8451
##       14
```

```
par(mfrow = c(1, 2))
plot(pre40, e40, xlab="Predicted", ylab="Pearson's residuals",main="Dispersion of RE vs PRED",cex.main=.8 )
abline(0,0)
qqnorm(e40, cex.main=.8)
qqline(e40)
```

### Ramified cells

```
#Fit mixed-effects model using dam as random factor
model41<-lme(CA1rad_type1~Prenatal*PD22, data=data_IBA1pd22, na.action=na.exclude, random=~1|Dam)
Anova(model41,type="III")
```

```
## Analysis of Deviance Table (Type III tests)
## 
## Response: CA1rad_type1
##                 Chisq Df Pr(>Chisq)    
## (Intercept)   85.3591  1     <2e-16 ***
## Prenatal       2.1132  1     0.1460    
## PD22           2.5721  1     0.1088    
## Prenatal:PD22  2.3449  1     0.1257    
## ---
## Signif. codes:  0 '***' 0.001 '**' 0.01 '*' 0.05 '.' 0.1 ' ' 1
```

```
#Normality and Homocedasticity checks
e41<-resid(model41) # Pearson's residuals
pre41<-predict(model41) #predicted
shapiro.test(e41)
```

```
## 
##  Shapiro-Wilk normality test
## 
## data:  e41
## W = 0.94325, p-value = 0.329
```

```
leveneTest(CA1or_type1~Prenatal*PD22, data=data_IBA1pd22)
```

```
## Levene's Test for Homogeneity of Variance (center = median)
##       Df F value Pr(>F)
## group  3  0.1212 0.9461
##       14
```

```
par(mfrow = c(1, 2))
plot(pre41, e41, xlab="Predicted", ylab="Pearson's residuals",main="Dispersion of RE vs PRED",cex.main=.8 )
abline(0,0)
qqnorm(e41, cex.main=.8)
qqline(e41)
```

### Hypertrophic cells

```
#Fit mixed-effects model using dam as random factor
model42<-lme(CA1rad_type2~Prenatal*PD22, data=data_IBA1pd22, na.action=na.exclude, random=~1|Dam)
Anova(model42,type="III")
```

```
## Analysis of Deviance Table (Type III tests)
## 
## Response: CA1rad_type2
##                 Chisq Df Pr(>Chisq)   
## (Intercept)   10.0416  1    0.00153 **
## Prenatal       0.5213  1    0.47028   
## PD22           0.3521  1    0.55293   
## Prenatal:PD22  1.1469  1    0.28420   
## ---
## Signif. codes:  0 '***' 0.001 '**' 0.01 '*' 0.05 '.' 0.1 ' ' 1
```

```
#Normality and Homocedasticity checks
e42<-resid(model42) # Pearson's residuals
pre42<-predict(model42) #predicted
shapiro.test(e42)
```

```
## 
##  Shapiro-Wilk normality test
## 
## data:  e42
## W = 0.91053, p-value = 0.08791
```

```
leveneTest(CA1rad_type2~Prenatal*PD22, data=data_IBA1pd22)
```

```
## Levene's Test for Homogeneity of Variance (center = median)
##       Df F value Pr(>F)
## group  3  0.0798 0.9699
##       14
```

```
par(mfrow = c(1, 2))
plot(pre42, e42, xlab="Predicted", ylab="Pearson's residuals",main="Dispersion of RE vs PRED",cex.main=.8 )
abline(0,0)
qqnorm(e42, cex.main=.8)
qqline(e42)
```

# GFAP AT PD36

```
data_GFAPpd36 <- read_excel("VPAxLPS_Data.xlsx", sheet = "GFAP_PD36")
data_GFAPpd36[data_GFAPpd36==""]<-NA
```

## SUPPLEMENTARY FIGURE 4G: GFAP in CA1 stratum oriens at PD36

```
#Fit mixed-effects model using dam as random factor
model43<-lme(CA1or~Prenatal*Juvenile, data=data_GFAPpd36, na.action=na.exclude, random=~1|Dam)
Anova(model43,type="III")
```

```
## Analysis of Deviance Table (Type III tests)
## 
## Response: CA1or
##                     Chisq Df Pr(>Chisq)    
## (Intercept)       62.4000  1  2.803e-15 ***
## Prenatal           0.0154  1    0.90128    
## Juvenile           3.4081  1    0.06488 .  
## Prenatal:Juvenile  0.5823  1    0.44542    
## ---
## Signif. codes:  0 '***' 0.001 '**' 0.01 '*' 0.05 '.' 0.1 ' ' 1
```

```
#Normality and Homocedasticity checks
e43<-resid(model43) # Pearson's residuals
pre43<-predict(model43) #predicted
shapiro.test(e43)
```

```
## 
##  Shapiro-Wilk normality test
## 
## data:  e43
## W = 0.94839, p-value = 0.4003
```

```
leveneTest(CA1or~Prenatal*Juvenile, data=data_GFAPpd36)
```

```
## Levene's Test for Homogeneity of Variance (center = median)
##       Df F value Pr(>F)
## group  3  0.7214 0.5557
##       14
```

```
par(mfrow = c(1, 2))
plot(pre43, e43, xlab="Predicted", ylab="Pearson's residuals",main="Dispersion of RE vs PRED",cex.main=.8 )
abline(0,0)
qqnorm(e43, cex.main=.8)
qqline(e43)
```

## SUPPLEMENTARY FIGURE 4H: GFAP in CA1 pyramidal cell layer at PD36

```
#Fit mixed-effects model using dam as random factor
model44<-lme(log(CA1pyr)~Prenatal*Juvenile, data=data_GFAPpd36, na.action=na.exclude, random=~1|Dam)
Anova(model44,type="III")
```

```
## Analysis of Deviance Table (Type III tests)
## 
## Response: log(CA1pyr)
##                      Chisq Df Pr(>Chisq)    
## (Intercept)       104.7555  1     <2e-16 ***
## Prenatal            0.1913  1     0.6619    
## Juvenile            1.2585  1     0.2619    
## Prenatal:Juvenile   0.8747  1     0.3497    
## ---
## Signif. codes:  0 '***' 0.001 '**' 0.01 '*' 0.05 '.' 0.1 ' ' 1
```

```
#Normality and Homocedasticity checks
e44<-resid(model44) # Pearson's residuals
pre44<-predict(model44) #predicted
shapiro.test(e44)
```

```
## 
##  Shapiro-Wilk normality test
## 
## data:  e44
## W = 0.95666, p-value = 0.5386
```

```
leveneTest(CA1pyr~Prenatal*Juvenile, data=data_GFAPpd36)
```

```
## Levene's Test for Homogeneity of Variance (center = median)
##       Df F value Pr(>F)
## group  3  0.2885 0.8329
##       14
```

```
par(mfrow = c(1, 2))
plot(pre44, e44, xlab="Predicted", ylab="Pearson's residuals",main="Dispersion of RE vs PRED",cex.main=.8 )
abline(0,0)
qqnorm(e44, cex.main=.8)
qqline(e44)
```

## SUPPLEMENTARY FIGURE 4I: GFAP in CA1 stratum radiatum at PD36

```
#Fit mixed-effects model using dam as random factor. Variance was models due to lack of homocedasticity of the data
model45 <- lme(CA1rad ~ Prenatal * Juvenile, 
               data = data_GFAPpd36, 
               na.action = na.exclude, 
               random = list(Dam = pdDiag(~1)))
Anova(model45,type="III")
```

```
## Analysis of Deviance Table (Type III tests)
## 
## Response: CA1rad
##                     Chisq Df Pr(>Chisq)    
## (Intercept)       63.2042  1  1.863e-15 ***
## Prenatal           0.0038  1    0.95064    
## Juvenile           2.8757  1    0.08993 .  
## Prenatal:Juvenile  0.7159  1    0.39750    
## ---
## Signif. codes:  0 '***' 0.001 '**' 0.01 '*' 0.05 '.' 0.1 ' ' 1
```

```
#Normality and Homocedasticity checks
e45<-resid(model45) # Pearson's residuals
pre45<-predict(model45) #predicted
shapiro.test(e45)
```

```
## 
##  Shapiro-Wilk normality test
## 
## data:  e45
## W = 0.91822, p-value = 0.1202
```

```
leveneTest(CA1rad~Prenatal*Juvenile, data=data_GFAPpd36)
```

```
## Levene's Test for Homogeneity of Variance (center = median)
##       Df F value  Pr(>F)  
## group  3  4.3398 0.02326 *
##       14                  
## ---
## Signif. codes:  0 '***' 0.001 '**' 0.01 '*' 0.05 '.' 0.1 ' ' 1
```

```
par(mfrow = c(1, 2))
plot(pre45, e45, xlab="Predicted", ylab="Pearson's residuals",main="Dispersion of RE vs PRED",cex.main=.8 )
abline(0,0)
qqnorm(e45, cex.main=.8)
qqline(e45)
```

# IBA1 AT PD36

```
data_IBA1pd36 <- read_excel("VPAxLPS_Data.xlsx", sheet = "IBA1_PD36")
data_IBA1pd36[data_IBA1pd36==""]<-NA
```

## SUPPLEMENTARY FIGURE 4J: IBA1 in hippocampal CA1 stratum oriens at PD36

### Total cells

```
#Fit mixed-effects model using dam as random factor
model46<-lme(CA1or_total~Prenatal*Juvenile, data=data_IBA1pd36, na.action=na.exclude, random=~1|Dam)
Anova(model46,type="III")
```

```
## Analysis of Deviance Table (Type III tests)
## 
## Response: CA1or_total
##                     Chisq Df Pr(>Chisq)    
## (Intercept)       36.9630  1  1.204e-09 ***
## Prenatal           0.3813  1     0.5369    
## Juvenile           1.2842  1     0.2571    
## Prenatal:Juvenile  2.0962  1     0.1477    
## ---
## Signif. codes:  0 '***' 0.001 '**' 0.01 '*' 0.05 '.' 0.1 ' ' 1
```

```
#Normality and Homocedasticity checks
e46<-resid(model46) # Pearson's residuals
pre46<-predict(model46) #predicted
shapiro.test(e46)
```

```
## 
##  Shapiro-Wilk normality test
## 
## data:  e46
## W = 0.89734, p-value = 0.05165
```

```
leveneTest(log(CA1or_total)~Prenatal*Juvenile, data=data_IBA1pd36)
```

```
## Levene's Test for Homogeneity of Variance (center = median)
##       Df F value Pr(>F)
## group  3  0.6969 0.5692
##       14
```

```
par(mfrow = c(1, 2))
plot(pre46, e46, xlab="Predicted", ylab="Pearson's residuals",main="Dispersion of RE vs PRED",cex.main=.8 )
abline(0,0)
qqnorm(e46, cex.main=.8)
qqline(e46)
```

### Ramified cells

```
#Fit mixed-effects model using dam as random factor
model47<-lme(log(CA1or_type1)~Prenatal*Juvenile, data=data_IBA1pd36, na.action=na.exclude, random=~1|Dam)
Anova(model47,type="III")
```

```
## Analysis of Deviance Table (Type III tests)
## 
## Response: log(CA1or_type1)
##                      Chisq Df Pr(>Chisq)    
## (Intercept)       305.4260  1     <2e-16 ***
## Prenatal            0.5796  1     0.4465    
## Juvenile            0.3245  1     0.5689    
## Prenatal:Juvenile   2.3599  1     0.1245    
## ---
## Signif. codes:  0 '***' 0.001 '**' 0.01 '*' 0.05 '.' 0.1 ' ' 1
```

```
#Normality and Homocedasticity checks
e47<-resid(model47) # Pearson's residuals
pre47<-predict(model47) #predicted
shapiro.test(e47)
```

```
## 
##  Shapiro-Wilk normality test
## 
## data:  e47
## W = 0.91785, p-value = 0.1184
```

```
leveneTest(log(CA1or_type1)~Prenatal*Juvenile, data=data_IBA1pd36)
```

```
## Levene's Test for Homogeneity of Variance (center = median)
##       Df F value Pr(>F)
## group  3  0.5345 0.6661
##       14
```

```
par(mfrow = c(1, 2))
plot(pre47, e47, xlab="Predicted", ylab="Pearson's residuals",main="Dispersion of RE vs PRED",cex.main=.8 )
abline(0,0)
qqnorm(e47, cex.main=.8)
qqline(e47)
```

### Hypertrophic cells

```
#Fit mixed-effects model using dam as random factor
model48<-lme(CA1or_type2~Prenatal*Juvenile, data=data_IBA1pd36, na.action=na.exclude, random=~1|Dam)
Anova(model48,type="III")
```

```
## Analysis of Deviance Table (Type III tests)
## 
## Response: CA1or_type2
##                    Chisq Df Pr(>Chisq)  
## (Intercept)       3.1622  1    0.07536 .
## Prenatal          0.0711  1    0.78972  
## Juvenile          1.4433  1    0.22960  
## Prenatal:Juvenile 0.1086  1    0.74174  
## ---
## Signif. codes:  0 '***' 0.001 '**' 0.01 '*' 0.05 '.' 0.1 ' ' 1
```

```
#Normality and Homocedasticity checks
e48<-resid(model48) # Pearson's residuals
pre48<-predict(model48) #predicted
shapiro.test(e48)
```

```
## 
##  Shapiro-Wilk normality test
## 
## data:  e48
## W = 0.95925, p-value = 0.5872
```

```
leveneTest(CA1or_type2~Prenatal*Juvenile, data=data_IBA1pd36)
```

```
## Levene's Test for Homogeneity of Variance (center = median)
##       Df F value Pr(>F)
## group  3  1.6573 0.2215
##       14
```

```
par(mfrow = c(1, 2))
plot(pre48, e48, xlab="Predicted", ylab="Pearson's residuals",main="Dispersion of RE vs PRED",cex.main=.8 )
abline(0,0)
qqnorm(e48, cex.main=.8)
qqline(e48)
```

## SUPPLEMENTARY FIGURE 4K: IBA1 in hippocampal CA1 pyramidal cell layer at PD36

### Total cells

```
#Fit mixed-effects model using dam as random factor
model49<-lme(CA1pyr_total~Prenatal*Juvenile, data=data_IBA1pd36, na.action=na.exclude, random=~1|Dam)
Anova(model49,type="III")
```

```
## Analysis of Deviance Table (Type III tests)
## 
## Response: CA1pyr_total
##                    Chisq Df Pr(>Chisq)    
## (Intercept)        8.929  1  0.0028068 ** 
## Prenatal           0.861  1  0.3534640    
## Juvenile          14.077  1  0.0001754 ***
## Prenatal:Juvenile  6.879  1  0.0087215 ** 
## ---
## Signif. codes:  0 '***' 0.001 '**' 0.01 '*' 0.05 '.' 0.1 ' ' 1
```

```
#Normality and Homocedasticity checks
e49<-resid(model49) # Pearson's residuals
pre49<-predict(model49) #predicted
shapiro.test(e49)
```

```
## 
##  Shapiro-Wilk normality test
## 
## data:  e49
## W = 0.98039, p-value = 0.954
```

```
leveneTest(CA1pyr_total~Prenatal*Juvenile, data=data_IBA1pd36)
```

```
## Levene's Test for Homogeneity of Variance (center = median)
##       Df F value Pr(>F)
## group  3  1.4627 0.2673
##       14
```

```
par(mfrow = c(1, 2))
plot(pre49, e49, xlab="Predicted", ylab="Pearson's residuals",main="Dispersion of RE vs PRED",cex.main=.8 )
abline(0,0)
qqnorm(e49, cex.main=.8)
qqline(e49)
```

```
#Posthoc comparisons
emmeans(model49, pairwise~Prenatal*Juvenile)
```

```
## $emmeans
##  Prenatal Juvenile emmean   SE df lower.CL upper.CL
##  Veh      LPS        5.73 1.92  5    0.801     10.7
##  VPA      LPS        8.45 2.22  4    2.299     14.6
##  Veh      SAL       12.18 1.76  5    7.648     16.7
##  VPA      SAL        7.91 2.48  4    1.035     14.8
## 
## Degrees-of-freedom method: containment 
## Confidence level used: 0.95 
## 
## $contrasts
##  contrast          estimate   SE df t.ratio p.value
##  Veh LPS - VPA LPS   -2.721 2.93  4  -0.928  0.7936
##  Veh LPS - Veh SAL   -6.444 1.72 10  -3.752  0.0167
##  Veh LPS - VPA SAL   -2.178 3.13  4  -0.695  0.8941
##  VPA LPS - Veh SAL   -3.723 2.83  4  -1.315  0.6009
##  VPA LPS - VPA SAL    0.542 2.04 10   0.266  0.9930
##  Veh SAL - VPA SAL    4.266 3.04  4   1.403  0.5581
## 
## Degrees-of-freedom method: containment 
## P value adjustment: tukey method for comparing a family of 4 estimates
```

### Ramified cells

```
#Fit mixed-effects model using dam as random factor
model50<-lme(CA1pyr_type1~Prenatal*Juvenile, data=data_IBA1pd36, na.action=na.exclude, random=~1|Dam)
Anova(model50,type="III")
```

```
## Analysis of Deviance Table (Type III tests)
## 
## Response: CA1pyr_type1
##                     Chisq Df Pr(>Chisq)    
## (Intercept)        7.0131  1   0.008091 ** 
## Prenatal           0.7030  1   0.401777    
## Juvenile          15.9444  1  6.523e-05 ***
## Prenatal:Juvenile  9.4742  1   0.002084 ** 
## ---
## Signif. codes:  0 '***' 0.001 '**' 0.01 '*' 0.05 '.' 0.1 ' ' 1
```

```
#Normality and Homocedasticity checks
e50<-resid(model50) # Pearson's residuals
pre50<-predict(model50) #predicted
shapiro.test(e50)
```

```
## 
##  Shapiro-Wilk normality test
## 
## data:  e50
## W = 0.98724, p-value = 0.9945
```

```
leveneTest(CA1pyr_type1~Prenatal*Juvenile, data=data_IBA1pd36)
```

```
## Levene's Test for Homogeneity of Variance (center = median)
##       Df F value Pr(>F)
## group  3  1.6135  0.231
##       14
```

```
par(mfrow = c(1, 2))
plot(pre50, e50, xlab="Predicted", ylab="Pearson's residuals",main="Dispersion of RE vs PRED",cex.main=.8 )
abline(0,0)
qqnorm(e50, cex.main=.8)
qqline(e50)
```

```
#Posthoc comparisons
emmeans(model50, pairwise~Prenatal*Juvenile)
```

```
## $emmeans
##  Prenatal Juvenile emmean   SE df lower.CL upper.CL
##  Veh      LPS        5.23 1.97  5    0.153     10.3
##  VPA      LPS        7.83 2.39  4    1.179     14.5
##  Veh      SAL       11.41 1.84  5    6.678     16.1
##  VPA      SAL        6.57 2.61  4   -0.672     13.8
## 
## Degrees-of-freedom method: containment 
## Confidence level used: 0.95 
## 
## $contrasts
##  contrast          estimate   SE df t.ratio p.value
##  Veh LPS - VPA LPS    -2.60 3.10  4  -0.838  0.8349
##  Veh LPS - Veh SAL    -6.19 1.55 10  -3.993  0.0114
##  Veh LPS - VPA SAL    -1.34 3.27  4  -0.410  0.9739
##  VPA LPS - Veh SAL    -3.58 3.02  4  -1.187  0.6649
##  VPA LPS - VPA SAL     1.26 1.86 10   0.679  0.9028
##  Veh SAL - VPA SAL     4.85 3.19  4   1.518  0.5049
## 
## Degrees-of-freedom method: containment 
## P value adjustment: tukey method for comparing a family of 4 estimates
```

### Hypertrophic cells

```
#Fit mixed-effects model using dam as random factor. Variance was modeled because of lack of homocedasticity
model51 <- lme(CA1pyr_type2 ~ Prenatal * Juvenile, 
               data = data_IBA1pd36, 
               na.action = na.exclude, 
               random = list(Dam = pdDiag(~1)))
Anova(model51,type="III")
```

```
## Analysis of Deviance Table (Type III tests)
## 
## Response: CA1pyr_type2
##                    Chisq Df Pr(>Chisq)
## (Intercept)       0.4634  1     0.4960
## Prenatal          0.6440  1     0.4223
## Juvenile          0.9709  1     0.3245
## Prenatal:Juvenile 0.3022  1     0.5825
```

```
#Normality and Homocedasticity checks
e51<-resid(model51) # Pearson's residuals
pre51<-predict(model51) #predicted
shapiro.test(e51)
```

```
## 
##  Shapiro-Wilk normality test
## 
## data:  e51
## W = 0.93829, p-value = 0.2708
```

```
leveneTest(CA1pyr_type2~Prenatal*Juvenile, data=data_IBA1pd36)
```

```
## Levene's Test for Homogeneity of Variance (center = median)
##       Df F value  Pr(>F)  
## group  3  4.8139 0.01658 *
##       14                  
## ---
## Signif. codes:  0 '***' 0.001 '**' 0.01 '*' 0.05 '.' 0.1 ' ' 1
```

```
par(mfrow = c(1, 2))
plot(pre51, e51, xlab="Predicted", ylab="Pearson's residuals",main="Dispersion of RE vs PRED",cex.main=.8 )
abline(0,0)
qqnorm(e51, cex.main=.8)
qqline(e51)
```

## SUPPLEMENTARY FIGURE 4L: IBA1 in hippocampal CA1 stratum radiatum at PD36

### Total cells

```
#Fit mixed-effects model using dam as random factor
model52<-lme(CA1rad_total~Prenatal*Juvenile, data=data_IBA1pd36, na.action=na.exclude, random=~1|Dam)
Anova(model52,type="III")
```

```
## Analysis of Deviance Table (Type III tests)
## 
## Response: CA1rad_total
##                     Chisq Df Pr(>Chisq)    
## (Intercept)       58.2025  1  2.365e-14 ***
## Prenatal           0.9149  1     0.3388    
## Juvenile           0.3320  1     0.5645    
## Prenatal:Juvenile  0.5553  1     0.4561    
## ---
## Signif. codes:  0 '***' 0.001 '**' 0.01 '*' 0.05 '.' 0.1 ' ' 1
```

```
#Normality and Homocedasticity checks
e52<-resid(model52) # Pearson's residuals
pre52<-predict(model52) #predicted
shapiro.test(e52)
```

```
## 
##  Shapiro-Wilk normality test
## 
## data:  e52
## W = 0.96785, p-value = 0.7565
```

```
leveneTest(CA1rad_total~Prenatal*Juvenile, data=data_IBA1pd36)
```

```
## Levene's Test for Homogeneity of Variance (center = median)
##       Df F value Pr(>F)
## group  3  0.1277 0.9421
##       14
```

```
par(mfrow = c(1, 2))
plot(pre52, e52, xlab="Predicted", ylab="Pearson's residuals",main="Dispersion of RE vs PRED",cex.main=.8 )
abline(0,0)
qqnorm(e52, cex.main=.8)
qqline(e52)
```

### Ramified cells

```
#Fit mixed-effects model using dam as random factor
model53<-lme(CA1rad_type1~Prenatal*Juvenile, data=data_IBA1pd36, na.action=na.exclude, random=~1|Dam)
Anova(model53,type="III")
```

```
## Analysis of Deviance Table (Type III tests)
## 
## Response: CA1rad_type1
##                     Chisq Df Pr(>Chisq)    
## (Intercept)       40.0644  1  2.457e-10 ***
## Prenatal           0.7596  1     0.3834    
## Juvenile           0.4750  1     0.4907    
## Prenatal:Juvenile  0.5542  1     0.4566    
## ---
## Signif. codes:  0 '***' 0.001 '**' 0.01 '*' 0.05 '.' 0.1 ' ' 1
```

```
#Normality and Homocedasticity checks
e53<-resid(model53) # Pearson's residuals
pre53<-predict(model53) #predicted
shapiro.test(e53)
```

```
## 
##  Shapiro-Wilk normality test
## 
## data:  e53
## W = 0.91672, p-value = 0.1131
```

```
leveneTest(CA1or_type1~Prenatal*Juvenile, data=data_IBA1pd36)
```

```
## Levene's Test for Homogeneity of Variance (center = median)
##       Df F value Pr(>F)
## group  3  0.5777 0.6392
##       14
```

```
par(mfrow = c(1, 2))
plot(pre53, e53, xlab="Predicted", ylab="Pearson's residuals",main="Dispersion of RE vs PRED",cex.main=.8 )
abline(0,0)
qqnorm(e53, cex.main=.8)
qqline(e53)
```

### Hypertrophic cells

```
#Fit mixed-effects model using dam as random factor
model54<-lme(CA1rad_type2~Prenatal*Juvenile, data=data_IBA1pd36, na.action=na.exclude, random=~1|Dam)
Anova(model54,type="III")
```

```
## Analysis of Deviance Table (Type III tests)
## 
## Response: CA1rad_type2
##                    Chisq Df Pr(>Chisq)   
## (Intercept)       9.1178  1   0.002531 **
## Prenatal          0.0359  1   0.849721   
## Juvenile          0.3204  1   0.571351   
## Prenatal:Juvenile 0.2507  1   0.616607   
## ---
## Signif. codes:  0 '***' 0.001 '**' 0.01 '*' 0.05 '.' 0.1 ' ' 1
```

```
#Normality and Homocedasticity checks
e54<-resid(model54) # Pearson's residuals
pre54<-predict(model54) #predicted
shapiro.test(e54)
```

```
## 
##  Shapiro-Wilk normality test
## 
## data:  e54
## W = 0.97429, p-value = 0.8733
```

```
leveneTest(CA1rad_type2~Prenatal*Juvenile, data=data_IBA1pd36)
```

```
## Levene's Test for Homogeneity of Variance (center = median)
##       Df F value Pr(>F)
## group  3  0.2352 0.8703
##       14
```

```
par(mfrow = c(1, 2))
plot(pre54, e54, xlab="Predicted", ylab="Pearson's residuals",main="Dispersion of RE vs PRED",cex.main=.8 )
abline(0,0)
qqnorm(e54, cex.main=.8)
qqline(e54)
```

# SUPPLEMENTARY FIGURE 5: NEUROINFLAMMATION IN THE HIPPOCAMPAL DG AT PD22 AND PD36

# GFAP AT PD22

```
data_GFAPpd22 <- read_excel("VPAxLPS_Data.xlsx", sheet = "GFAP_PD22")
data_GFAPpd22[data_GFAPpd22==""]<-NA
```

## SUPPLEMENTARY FIGURE 5A: GFAP in DG molecular layer at PD22

```
#Fit mixed-effects model using Dam as random factor.
model55<-lme(DGmol~Prenatal*PD22, data=data_GFAPpd22, na.action=na.exclude, random=~1|Dam)
Anova(model55, type="III")
```

```
## Analysis of Deviance Table (Type III tests)
## 
## Response: DGmol
##                 Chisq Df Pr(>Chisq)    
## (Intercept)   98.2728  1    < 2e-16 ***
## Prenatal       1.1359  1    0.28652    
## PD22           0.3474  1    0.55557    
## Prenatal:PD22  4.2071  1    0.04025 *  
## ---
## Signif. codes:  0 '***' 0.001 '**' 0.01 '*' 0.05 '.' 0.1 ' ' 1
```

```
#Normality and Homocedasticity checks
e55<-resid(model55) # Pearson's residuals
pre55<-predict(model55) #predicted
shapiro.test(e55)
```

```
## 
##  Shapiro-Wilk normality test
## 
## data:  e55
## W = 0.95852, p-value = 0.6667
```

```
leveneTest(DGmol~Prenatal*PD22, data=data_GFAPpd22)
```

```
## Levene's Test for Homogeneity of Variance (center = median)
##       Df F value Pr(>F)
## group  3  0.6466 0.6012
##       11
```

```
par(mfrow = c(1, 2))
plot(pre55, e55, xlab="Predicted", ylab="Pearson's residuals",main="Dispersion of RE vs PRED",cex.main=.8 )
abline(0,0)
qqnorm(e55, cex.main=.8)
qqline(e55)
```

```
#Posthoc comparisons
emmeans(model55, pairwise~Prenatal*PD22)
```

```
## $emmeans
##  Prenatal PD22 emmean   SE df lower.CL upper.CL
##  VEH      LPS    35.1 3.54 10     27.2     43.0
##  VPA      LPS    29.8 3.54  9     21.8     37.8
##  VEH      SAL    38.1 3.54  2     22.8     53.3
##  VPA      SAL    47.9 4.09  2     30.3     65.5
## 
## Degrees-of-freedom method: containment 
## Confidence level used: 0.95 
## 
## $contrasts
##  contrast          estimate   SE df t.ratio p.value
##  VEH LPS - VPA LPS     5.34 5.01  9   1.066  0.7174
##  VEH LPS - VEH SAL    -2.95 5.01  2  -0.589  0.9271
##  VEH LPS - VPA SAL   -12.74 5.41  2  -2.354  0.3255
##  VPA LPS - VEH SAL    -8.29 5.01  2  -1.655  0.5084
##  VPA LPS - VPA SAL   -18.08 5.41  2  -3.341  0.1884
##  VEH SAL - VPA SAL    -9.79 5.41  2  -1.808  0.4598
## 
## Degrees-of-freedom method: containment 
## P value adjustment: tukey method for comparing a family of 4 estimates
```

## SUPPLEMENTARY FIGURE 5B: GFAP in DG granular cell layer at PD22

```
#Fit mixed-effects model using Dam as random factor.
model56<-lme(DGgran~Prenatal*PD22, data=data_GFAPpd22, na.action=na.exclude, random=~1|Dam)
Anova(model56,type="III")
```

```
## Analysis of Deviance Table (Type III tests)
## 
## Response: DGgran
##                Chisq Df Pr(>Chisq)   
## (Intercept)   7.2064  1   0.007265 **
## Prenatal      0.9704  1   0.324591   
## PD22          1.2270  1   0.267998   
## Prenatal:PD22 0.0087  1   0.925606   
## ---
## Signif. codes:  0 '***' 0.001 '**' 0.01 '*' 0.05 '.' 0.1 ' ' 1
```

```
#Normality and Homocedasticity checks
e56<-resid(model56) # Pearson's residuals
pre56<-predict(model56) #predicted
shapiro.test(e56)
```

```
## 
##  Shapiro-Wilk normality test
## 
## data:  e56
## W = 0.96876, p-value = 0.8393
```

```
leveneTest(DGgran~Prenatal*PD22, data=data_GFAPpd22)
```

```
## Levene's Test for Homogeneity of Variance (center = median)
##       Df F value Pr(>F)
## group  3  0.3072 0.8197
##       11
```

```
par(mfrow = c(1, 2))
plot(pre56, e56, xlab="Predicted", ylab="Pearson's residuals",main="Dispersion of RE vs PRED",cex.main=.8 )
abline(0,0)
qqnorm(e56, cex.main=.8)
qqline(e56)
```

## SUPPLEMENTARY FIGURE 5C: GFAP in DG hilus at PD22

```
#Fit mixed-effects model using Dam as random factor.
#model57<-gls(log(DGhilus)~Prenatal*PD22, data=data_GFAPpd22, na.action=na.exclude)
model57<-lme(log(DGhilus)~Prenatal*PD22, data=data_GFAPpd22, na.action=na.exclude, random=~1|Dam)
Anova(model57, type="III")
```

```
## Analysis of Deviance Table (Type III tests)
## 
## Response: log(DGhilus)
##                  Chisq Df Pr(>Chisq)    
## (Intercept)   649.2461  1     <2e-16 ***
## Prenatal        2.5607  1     0.1095    
## PD22            1.8963  1     0.1685    
## Prenatal:PD22   0.8382  1     0.3599    
## ---
## Signif. codes:  0 '***' 0.001 '**' 0.01 '*' 0.05 '.' 0.1 ' ' 1
```

```
#Normality and Homocedasticity checks
e57<-resid(model57) # Pearson's residuals
pre57<-predict(model57) #predicted
shapiro.test(e57)
```

```
## 
##  Shapiro-Wilk normality test
## 
## data:  e57
## W = 0.844, p-value = 0.0143
```

```
leveneTest(DGhilus~Prenatal*PD22, data=data_GFAPpd22)
```

```
## Levene's Test for Homogeneity of Variance (center = median)
##       Df F value Pr(>F)
## group  3  1.1404 0.3755
##       11
```

```
par(mfrow = c(1, 2))
plot(pre57, e57, xlab="Predicted", ylab="Pearson's residuals",main="Dispersion of RE vs PRED",cex.main=.8 )
abline(0,0)
qqnorm(e57, cex.main=.8)
qqline(e57)
```

# IBA1 AT PD22

```
data_IBA1pd22 <- read_excel("VPAxLPS_Data.xlsx", sheet = "IBA1_PD22")
#my_data <- read_excel("my_file.xlsx", sheet = "data")
data_IBA1pd22[data_IBA1pd22==""]<-NA
```

## SUPPLEMENTARY FIGURE 5D: IBA1 in hippocampal DG molecular layer at PD22

### Total cells

```
#Fit mixed-effects model using dam as random factor
model58<-lme(DGmol_total~Prenatal*PD22, data=data_IBA1pd22, na.action=na.exclude, random=~1|Dam)
Anova(model58,type="III")
```

```
## Analysis of Deviance Table (Type III tests)
## 
## Response: DGmol_total
##                 Chisq Df Pr(>Chisq)    
## (Intercept)   41.7274  1  1.049e-10 ***
## Prenatal       1.3507  1  0.2451625    
## PD22           5.4690  1  0.0193569 *  
## Prenatal:PD22 13.1047  1  0.0002945 ***
## ---
## Signif. codes:  0 '***' 0.001 '**' 0.01 '*' 0.05 '.' 0.1 ' ' 1
```

```
#Normality and Homocedasticity checks
e58<-resid(model58) # Pearson's residuals
pre58<-predict(model58) #predicted
shapiro.test(e58)
```

```
## 
##  Shapiro-Wilk normality test
## 
## data:  e58
## W = 0.98389, p-value = 0.9811
```

```
leveneTest(DGmol_total~Prenatal*PD22, data=data_IBA1pd22)
```

```
## Levene's Test for Homogeneity of Variance (center = median)
##       Df F value Pr(>F)
## group  3  2.2739 0.1248
##       14
```

```
par(mfrow = c(1, 2))
plot(pre58, e58, xlab="Predicted", ylab="Pearson's residuals",main="Dispersion of RE vs PRED",cex.main=.8 )
abline(0,0)
qqnorm(e58, cex.main=.8)
qqline(e58)
```

```
#Posthoc comparisons
emmeans(model58, pairwise~Prenatal*PD22)
```

```
## $emmeans
##  Prenatal PD22 emmean   SE df lower.CL upper.CL
##  VEH      LPS    11.5 1.78 11     7.57     15.4
##  VPA      LPS    14.5 1.96 10    10.18     18.9
##  VEH      SAL    14.6 1.70  4     9.87     19.3
##  VPA      SAL    11.3 1.95  4     5.89     16.7
## 
## Degrees-of-freedom method: containment 
## Confidence level used: 0.95 
## 
## $contrasts
##  contrast          estimate   SE df t.ratio p.value
##  VEH LPS - VPA LPS  -3.0726 2.64 10  -1.162  0.6622
##  VEH LPS - VEH SAL  -3.1231 1.34  4  -2.339  0.2321
##  VEH LPS - VPA SAL   0.1602 2.64  4   0.061  0.9999
##  VPA LPS - VEH SAL  -0.0505 2.60  4  -0.019  1.0000
##  VPA LPS - VPA SAL   3.2328 1.14  4   2.836  0.1447
##  VEH SAL - VPA SAL   3.2832 2.59  4   1.266  0.6250
## 
## Degrees-of-freedom method: containment 
## P value adjustment: tukey method for comparing a family of 4 estimates
```

### Ramified cells

```
#Fit mixed-effects model using dam as random factor
model59<-lme(DGmol_type1~Prenatal*PD22, data=data_IBA1pd22, na.action=na.exclude, random=~1|Dam)
Anova(model59, type="III")
```

```
## Analysis of Deviance Table (Type III tests)
## 
## Response: DGmol_type1
##                 Chisq Df Pr(>Chisq)    
## (Intercept)   45.6460  1  1.417e-11 ***
## Prenatal       0.8626  1   0.353020    
## PD22           2.5595  1   0.109631    
## Prenatal:PD22  9.9851  1   0.001578 ** 
## ---
## Signif. codes:  0 '***' 0.001 '**' 0.01 '*' 0.05 '.' 0.1 ' ' 1
```

```
#Normality and Homocedasticity checks
e59<-resid(model59) # Pearson's residuals
pre59<-predict(model59) #predicted
shapiro.test(e59)
```

```
## 
##  Shapiro-Wilk normality test
## 
## data:  e59
## W = 0.94175, p-value = 0.3104
```

```
leveneTest(DGmol_type1~Prenatal*PD22, data=data_IBA1pd22)
```

```
## Levene's Test for Homogeneity of Variance (center = median)
##       Df F value Pr(>F)
## group  3  1.0427 0.4042
##       14
```

```
par(mfrow = c(1, 2))
plot(pre59, e59, xlab="Predicted", ylab="Pearson's residuals",main="Dispersion of RE vs PRED",cex.main=.8 )
abline(0,0)
qqnorm(e59, cex.main=.8)
qqline(e59)
```

```
#Posthoc comparisons
emmeans(model59, pairwise~Prenatal*PD22)
```

```
## $emmeans
##  Prenatal PD22 emmean   SE df lower.CL upper.CL
##  VEH      LPS    11.5 1.70 11     7.73     15.2
##  VPA      LPS    13.8 1.85 10     9.67     17.9
##  VEH      SAL    13.6 1.62  4     9.15     18.1
##  VPA      SAL    10.3 1.85  4     5.16     15.4
## 
## Degrees-of-freedom method: containment 
## Confidence level used: 0.95 
## 
## $contrasts
##  contrast          estimate   SE df t.ratio p.value
##  VEH LPS - VPA LPS   -2.331 2.51 10  -0.929  0.7906
##  VEH LPS - VEH SAL   -2.184 1.37  4  -1.600  0.4692
##  VEH LPS - VPA SAL    1.176 2.51  4   0.469  0.9621
##  VPA LPS - VEH SAL    0.147 2.46  4   0.060  0.9999
##  VPA LPS - VPA SAL    3.507 1.17  4   2.985  0.1261
##  VEH SAL - VPA SAL    3.360 2.45  4   1.369  0.5743
## 
## Degrees-of-freedom method: containment 
## P value adjustment: tukey method for comparing a family of 4 estimates
```

### Hypertrophic cells

```
#Fit mixed-effects model using dam as random factor
model60<-lme(log(1+DGmol_type2)~Prenatal*PD22, data=data_IBA1pd22, na.action=na.exclude, random=~1|Dam)
Anova(model60, type="III")
```

```
## Analysis of Deviance Table (Type III tests)
## 
## Response: log(1 + DGmol_type2)
##                Chisq Df Pr(>Chisq)
## (Intercept)   2.2033  1     0.1377
## Prenatal      0.2002  1     0.6545
## PD22          0.1532  1     0.6955
## Prenatal:PD22 0.3400  1     0.5599
```

```
#Normality and Homocedasticity checks
e60<-resid(model60) # Pearson's residuals
pre60<-predict(model60) #predicted
shapiro.test(e60)
```

```
## 
##  Shapiro-Wilk normality test
## 
## data:  e60
## W = 0.85634, p-value = 0.01068
```

```
leveneTest(DGmol_type2~Prenatal*PD22, data=data_IBA1pd22)
```

```
## Levene's Test for Homogeneity of Variance (center = median)
##       Df F value Pr(>F)
## group  3  0.2276 0.8756
##       14
```

```
par(mfrow = c(1, 2))
plot(pre60, e60, xlab="Predicted", ylab="Pearson's residuals",main="Dispersion of RE vs PRED",cex.main=.8 )
abline(0,0)
qqnorm(e60, cex.main=.8)
qqline(e60)
```

## SUPPLEMENTARY FIGURE 5E: IBA1 in hippocampal DG granular cell layer at PD22

### Total cells

```
#Fit mixed-effects model using dam as random factor
model61<-lme(log(DGgran_total)~Prenatal*PD22, data=data_IBA1pd22, na.action=na.exclude, random=~1|Dam)
Anova(model61,type="III")
```

```
## Analysis of Deviance Table (Type III tests)
## 
## Response: log(DGgran_total)
##                  Chisq Df Pr(>Chisq)    
## (Intercept)   150.8627  1     <2e-16 ***
## Prenatal        0.5848  1     0.4444    
## PD22            2.2131  1     0.1368    
## Prenatal:PD22   0.2865  1     0.5925    
## ---
## Signif. codes:  0 '***' 0.001 '**' 0.01 '*' 0.05 '.' 0.1 ' ' 1
```

```
#Normality and Homocedasticity checks
e61<-resid(model61) # Pearson's residuals
pre61<-predict(model61) #predicted
shapiro.test(e61)
```

```
## 
##  Shapiro-Wilk normality test
## 
## data:  e61
## W = 0.8867, p-value = 0.03389
```

```
leveneTest(DGgran_total~Prenatal*PD22, data=data_IBA1pd22)
```

```
## Levene's Test for Homogeneity of Variance (center = median)
##       Df F value Pr(>F)
## group  3  0.1783 0.9093
##       14
```

```
par(mfrow = c(1, 2))
plot(pre61, e61, xlab="Predicted", ylab="Pearson's residuals",main="Dispersion of RE vs PRED",cex.main=.8 )
abline(0,0)
qqnorm(e61, cex.main=.8)
qqline(e61)
```

### Ramified cells

```
#Fit mixed-effects model using dam as random factor
model62<-lme(log(DGgran_type1)~Prenatal*PD22, data=data_IBA1pd22, na.action=na.exclude, random=~1|Dam)
Anova(model62,type="III")
```

```
## Analysis of Deviance Table (Type III tests)
## 
## Response: log(DGgran_type1)
##                  Chisq Df Pr(>Chisq)    
## (Intercept)   148.0788  1     <2e-16 ***
## Prenatal        0.7480  1     0.3871    
## PD22            2.0589  1     0.1513    
## Prenatal:PD22   0.3891  1     0.5328    
## ---
## Signif. codes:  0 '***' 0.001 '**' 0.01 '*' 0.05 '.' 0.1 ' ' 1
```

```
#Normality and Homocedasticity checks
e62<-resid(model62) # Pearson's residuals
pre62<-predict(model62) #predicted
shapiro.test(e62)
```

```
## 
##  Shapiro-Wilk normality test
## 
## data:  e62
## W = 0.87476, p-value = 0.02133
```

```
leveneTest(DGgran_type1~Prenatal*PD22, data=data_IBA1pd22)
```

```
## Levene's Test for Homogeneity of Variance (center = median)
##       Df F value Pr(>F)
## group  3  0.2216 0.8798
##       14
```

```
par(mfrow = c(1, 2))
plot(pre62, e62, xlab="Predicted", ylab="Pearson's residuals",main="Dispersion of RE vs PRED",cex.main=.8 )
abline(0,0)
qqnorm(e62, cex.main=.8)
qqline(e62)
```

### Hypertrophic cells

```
#Fit mixed-effects model using dam as random factor
model63<-lme(DGgran_type2~Prenatal*PD22, data=data_IBA1pd22, na.action=na.exclude, random=~1|Dam)
Anova(model63,type="III")
```

```
## Analysis of Deviance Table (Type III tests)
## 
## Response: DGgran_type2
##                Chisq Df Pr(>Chisq)  
## (Intercept)   6.4321  1    0.01121 *
## Prenatal      0.0368  1    0.84793  
## PD22          1.8213  1    0.17716  
## Prenatal:PD22 0.0023  1    0.96215  
## ---
## Signif. codes:  0 '***' 0.001 '**' 0.01 '*' 0.05 '.' 0.1 ' ' 1
```

```
#Normality and Homocedasticity checks
e63<-resid(model63) # Pearson's residuals
pre63<-predict(model63) #predicted
shapiro.test(e63)
```

```
## 
##  Shapiro-Wilk normality test
## 
## data:  e63
## W = 0.93689, p-value = 0.2562
```

```
leveneTest(DGgran_type2~Prenatal*PD22, data=data_IBA1pd22)
```

```
## Levene's Test for Homogeneity of Variance (center = median)
##       Df F value Pr(>F)
## group  3  2.2447 0.1281
##       14
```

```
par(mfrow = c(1, 2))
plot(pre63, e63, xlab="Predicted", ylab="Pearson's residuals",main="Dispersion of RE vs PRED",cex.main=.8 )
abline(0,0)
qqnorm(e63, cex.main=.8)
qqline(e63)
```

## SUPPLEMENTARY FIGURE 5F: IBA1 in hippocampal DG hilus at PD22

### Total cells

```
#Fit mixed-effects model using dam as random factor
model64<-lme(DGhilus_total~Prenatal*PD22, data=data_IBA1pd22, na.action=na.exclude, random=~1|Dam)
Anova(model64,type="III")
```

```
## Analysis of Deviance Table (Type III tests)
## 
## Response: DGhilus_total
##                 Chisq Df Pr(>Chisq)    
## (Intercept)   46.6958  1  8.291e-12 ***
## Prenatal       0.0691  1     0.7927    
## PD22           0.0062  1     0.9373    
## Prenatal:PD22  0.0294  1     0.8639    
## ---
## Signif. codes:  0 '***' 0.001 '**' 0.01 '*' 0.05 '.' 0.1 ' ' 1
```

```
#Normality and Homocedasticity checks
e64<-resid(model64) # Pearson's residuals
pre64<-predict(model64) #predicted
shapiro.test(e64)
```

```
## 
##  Shapiro-Wilk normality test
## 
## data:  e64
## W = 0.94701, p-value = 0.3801
```

```
leveneTest(DGhilus_total~Prenatal*PD22, data=data_IBA1pd22)
```

```
## Levene's Test for Homogeneity of Variance (center = median)
##       Df F value Pr(>F)
## group  3  0.1788  0.909
##       14
```

```
par(mfrow = c(1, 2))
plot(pre64, e64, xlab="Predicted", ylab="Pearson's residuals",main="Dispersion of RE vs PRED",cex.main=.8 )
abline(0,0)
qqnorm(e64, cex.main=.8)
qqline(e64)
```

### Ramified cells

```
#Fit mixed-effects model using dam as random factor
model65<-lme(DGhilus_type1~Prenatal*PD22, data=data_IBA1pd22, na.action=na.exclude, random=~1|Dam)
Anova(model65,type="III")
```

```
## Analysis of Deviance Table (Type III tests)
## 
## Response: DGhilus_type1
##                 Chisq Df Pr(>Chisq)    
## (Intercept)   46.0844  1  1.133e-11 ***
## Prenatal       0.1681  1     0.6818    
## PD22           0.0593  1     0.8076    
## Prenatal:PD22  0.2093  1     0.6473    
## ---
## Signif. codes:  0 '***' 0.001 '**' 0.01 '*' 0.05 '.' 0.1 ' ' 1
```

```
#Normality and Homocedasticity checks
e65<-resid(model65) # Pearson's residuals
pre65<-predict(model65) #predicted
shapiro.test(e65)
```

```
## 
##  Shapiro-Wilk normality test
## 
## data:  e65
## W = 0.93134, p-value = 0.205
```

```
leveneTest(DGhilus_type1~Prenatal*PD22, data=data_IBA1pd22)
```

```
## Levene's Test for Homogeneity of Variance (center = median)
##       Df F value Pr(>F)
## group  3  0.3436 0.7943
##       14
```

```
par(mfrow = c(1, 2))
plot(pre65, e65, xlab="Predicted", ylab="Pearson's residuals",main="Dispersion of RE vs PRED",cex.main=.8 )
abline(0,0)
qqnorm(e65, cex.main=.8)
qqline(e65)
```

### Hypertrophic cells

```
#Fit mixed-effects model using dam as random factor
model66<-lme(DGhilus_type2~Prenatal*PD22, data=data_IBA1pd22, na.action=na.exclude, random=~1|Dam)
Anova(model66,type="III")
```

```
## Analysis of Deviance Table (Type III tests)
## 
## Response: DGhilus_type2
##                Chisq Df Pr(>Chisq)  
## (Intercept)   2.8339  1    0.09229 .
## Prenatal      0.7683  1    0.38073  
## PD22          1.0375  1    0.30840  
## Prenatal:PD22 3.3660  1    0.06655 .
## ---
## Signif. codes:  0 '***' 0.001 '**' 0.01 '*' 0.05 '.' 0.1 ' ' 1
```

```
#Normality and Homocedasticity checks
e66<-resid(model66) # Pearson's residuals
pre66<-predict(model66) #predicted
shapiro.test(e66)
```

```
## 
##  Shapiro-Wilk normality test
## 
## data:  e66
## W = 0.90384, p-value = 0.06706
```

```
leveneTest(DGhilus_type2~Prenatal*PD22, data=data_IBA1pd22)
```

```
## Levene's Test for Homogeneity of Variance (center = median)
##       Df F value Pr(>F)
## group  3  1.2796 0.3198
##       14
```

```
par(mfrow = c(1, 2))
plot(pre66, e66, xlab="Predicted", ylab="Pearson's residuals",main="Dispersion of RE vs PRED",cex.main=.8 )
abline(0,0)
qqnorm(e66, cex.main=.8)
qqline(e66)
```

# GFAP AT PD36

```
data_GFAPpd36 <- read_excel("VPAxLPS_Data.xlsx", sheet = "GFAP_PD36")
data_GFAPpd36[data_GFAPpd36==""]<-NA
```

## SUPPLEMENTARY FIGURE 5G: GFAP in DG molecular layer at PD36

```
#Fit linear model using generalized least squares and ANOVA
model67<-lme(DGmol~Prenatal*Juvenile, data=data_GFAPpd36, na.action=na.exclude, random=~1|Dam)
Anova(model67,type="III")
```

```
## Analysis of Deviance Table (Type III tests)
## 
## Response: DGmol
##                     Chisq Df Pr(>Chisq)    
## (Intercept)       84.3207  1     <2e-16 ***
## Prenatal           0.4431  1     0.5057    
## Juvenile           1.0194  1     0.3127    
## Prenatal:Juvenile  0.0012  1     0.9726    
## ---
## Signif. codes:  0 '***' 0.001 '**' 0.01 '*' 0.05 '.' 0.1 ' ' 1
```

```
#Normality and Homocedasticity checks
e67<-resid(model67) # Pearson's residuals
pre67<-predict(model67) #predicted
shapiro.test(e67)
```

```
## 
##  Shapiro-Wilk normality test
## 
## data:  e67
## W = 0.97966, p-value = 0.9464
```

```
leveneTest(DGmol~Prenatal*Juvenile, data=data_GFAPpd36)
```

```
## Levene's Test for Homogeneity of Variance (center = median)
##       Df F value Pr(>F)
## group  3  1.6737 0.2181
##       14
```

```
par(mfrow = c(1, 2))
plot(pre67, e67, xlab="Predicted", ylab="Pearson's residuals",main="Dispersion of RE vs PRED",cex.main=.8 )
abline(0,0)
qqnorm(e67, cex.main=.8)
qqline(e67)
```

## SUPPLEMENTARY FIGURE 5H: GFAP in DG granular cell layer at PD36

```
#Fit mixed-effects model using dam as random factor
model68<-lme(DGgran~Prenatal*Juvenile, data=data_GFAPpd36, na.action=na.exclude, random=~1|Dam)
Anova(model68,type="III")
```

```
## Analysis of Deviance Table (Type III tests)
## 
## Response: DGgran
##                    Chisq Df Pr(>Chisq)  
## (Intercept)       5.0101  1     0.0252 *
## Prenatal          0.0485  1     0.8257  
## Juvenile          0.8658  1     0.3521  
## Prenatal:Juvenile 1.0474  1     0.3061  
## ---
## Signif. codes:  0 '***' 0.001 '**' 0.01 '*' 0.05 '.' 0.1 ' ' 1
```

```
#Normality and Homocedasticity checks
e68<-resid(model68) # Pearson's residuals
pre68<-predict(model68) #predicted
shapiro.test(e68)
```

```
## 
##  Shapiro-Wilk normality test
## 
## data:  e68
## W = 0.95658, p-value = 0.5372
```

```
leveneTest(DGgran~Prenatal*Juvenile, data=data_GFAPpd36)
```

```
## Levene's Test for Homogeneity of Variance (center = median)
##       Df F value Pr(>F)
## group  3    1.77  0.199
##       14
```

```
par(mfrow = c(1, 2))
plot(pre68, e68, xlab="Predicted", ylab="Pearson's residuals",main="Dispersion of RE vs PRED",cex.main=.8 )
abline(0,0)
qqnorm(e68, cex.main=.8)
qqline(e68)
```

## SUPPLEMENTARY FIGURE 5I: GFAP in DG hilus at PD36

```
#Fit mixed-effects model using dam as random factor
model69<-lme(DGhilus~Prenatal*Juvenile, data=data_GFAPpd36, na.action=na.exclude, random=~1|Dam)
Anova(model69,type="III")
```

```
## Analysis of Deviance Table (Type III tests)
## 
## Response: DGhilus
##                     Chisq Df Pr(>Chisq)    
## (Intercept)       51.8203  1  6.082e-13 ***
## Prenatal           2.7009  1     0.1003    
## Juvenile           0.3448  1     0.5571    
## Prenatal:Juvenile  0.8225  1     0.3645    
## ---
## Signif. codes:  0 '***' 0.001 '**' 0.01 '*' 0.05 '.' 0.1 ' ' 1
```

```
#Normality and Homocedasticity checks
e69<-resid(model69) # Pearson's residuals
pre69<-predict(model69) #predicted
shapiro.test(e69)
```

```
## 
##  Shapiro-Wilk normality test
## 
## data:  e69
## W = 0.9152, p-value = 0.1063
```

```
leveneTest(DGhilus~Prenatal*Juvenile, data=data_GFAPpd36)
```

```
## Levene's Test for Homogeneity of Variance (center = median)
##       Df F value Pr(>F)
## group  3  1.9419 0.1693
##       14
```

```
par(mfrow = c(1, 2))
plot(pre69, e69, xlab="Predicted", ylab="Pearson's residuals",main="Dispersion of RE vs PRED",cex.main=.8 )
abline(0,0)
qqnorm(e69, cex.main=.8)
qqline(e69)
```

# IBA1 AT PD36

```
data_IBA1pd36 <- read_excel("VPAxLPS_Data.xlsx", sheet = "IBA1_PD36")
data_IBA1pd36[data_IBA1pd36==""]<-NA
```

## SUPPLEMENTARY FIGURE 5J: IBA1 in hippocampal DG molecular layer at PD36

### Total cells

```
#Fit mixed-effects model using dam as random factor
model70<-lme(DGmol_total~Prenatal*Juvenile, data=data_IBA1pd36, na.action=na.exclude, random=~1|Dam)
Anova(model70,type="III")
```

```
## Analysis of Deviance Table (Type III tests)
## 
## Response: DGmol_total
##                     Chisq Df Pr(>Chisq)    
## (Intercept)       55.7783  1  8.112e-14 ***
## Prenatal           0.0001  1     0.9928    
## Juvenile           0.1264  1     0.7222    
## Prenatal:Juvenile  0.0020  1     0.9646    
## ---
## Signif. codes:  0 '***' 0.001 '**' 0.01 '*' 0.05 '.' 0.1 ' ' 1
```

```
#Normality and Homocedasticity checks
e70<-resid(model70) # Pearson's residuals
pre70<-predict(model70) #predicted
shapiro.test(e70)
```

```
## 
##  Shapiro-Wilk normality test
## 
## data:  e70
## W = 0.98762, p-value = 0.9954
```

```
leveneTest(DGmol_total~Prenatal*Juvenile, data=data_IBA1pd36)
```

```
## Levene's Test for Homogeneity of Variance (center = median)
##       Df F value Pr(>F)
## group  3  0.6755 0.5813
##       14
```

```
par(mfrow = c(1, 2))
plot(pre70, e70, xlab="Predicted", ylab="Pearson's residuals",main="Dispersion of RE vs PRED",cex.main=.8 )
abline(0,0)
qqnorm(e70, cex.main=.8)
qqline(e70)
```

### Ramified cells

```
#Fit mixed-effects model using dam as random factor
model71<-lme(DGmol_type1~Prenatal*Juvenile, data=data_IBA1pd36, na.action=na.exclude, random=~1|Dam)
Anova(model71,type="III")
```

```
## Analysis of Deviance Table (Type III tests)
## 
## Response: DGmol_type1
##                     Chisq Df Pr(>Chisq)    
## (Intercept)       38.5210  1  5.417e-10 ***
## Prenatal           0.0264  1     0.8710    
## Juvenile           0.2866  1     0.5924    
## Prenatal:Juvenile  0.0390  1     0.8434    
## ---
## Signif. codes:  0 '***' 0.001 '**' 0.01 '*' 0.05 '.' 0.1 ' ' 1
```

```
#Normality and Homocedasticity checks
e71<-resid(model71) # Pearson's residuals
pre71<-predict(model71) #predicted
shapiro.test(e71)
```

```
## 
##  Shapiro-Wilk normality test
## 
## data:  e71
## W = 0.96351, p-value = 0.6704
```

```
leveneTest(DGmol_type1~Prenatal*Juvenile, data=data_IBA1pd36)
```

```
## Levene's Test for Homogeneity of Variance (center = median)
##       Df F value Pr(>F)
## group  3  0.5307 0.6686
##       14
```

```
par(mfrow = c(1, 2))
plot(pre71, e71, xlab="Predicted", ylab="Pearson's residuals",main="Dispersion of RE vs PRED",cex.main=.8 )
abline(0,0)
qqnorm(e71, cex.main=.8)
qqline(e71)
```

### Hypertrophic cells

```
#Fit mixed-effects model using dam as random factor
model72<-lme(DGmol_type2~Prenatal*Juvenile, data=data_IBA1pd36, na.action=na.exclude, random=~1|Dam)
Anova(model72,type="III")
```

```
## Analysis of Deviance Table (Type III tests)
## 
## Response: DGmol_type2
##                     Chisq Df Pr(>Chisq)    
## (Intercept)       11.0948  1  0.0008657 ***
## Prenatal           1.0222  1  0.3119956    
## Juvenile           1.1428  1  0.2850536    
## Prenatal:Juvenile  0.4427  1  0.5058228    
## ---
## Signif. codes:  0 '***' 0.001 '**' 0.01 '*' 0.05 '.' 0.1 ' ' 1
```

```
#Normality and Homocedasticity checks
e72<-resid(model72) # Pearson's residuals
pre72<-predict(model72) #predicted
shapiro.test(e72)
```

```
## 
##  Shapiro-Wilk normality test
## 
## data:  e72
## W = 0.95061, p-value = 0.4347
```

```
leveneTest(DGmol_type2~Prenatal*Juvenile, data=data_IBA1pd36)
```

```
## Levene's Test for Homogeneity of Variance (center = median)
##       Df F value Pr(>F)
## group  3  0.6458 0.5983
##       14
```

```
par(mfrow = c(1, 2))
plot(pre72, e72, xlab="Predicted", ylab="Pearson's residuals",main="Dispersion of RE vs PRED",cex.main=.8 )
abline(0,0)
qqnorm(e72, cex.main=.8)
qqline(e72)
```

## SUPPLEMENTARY FIGURE 5K: IBA1 in hippocampal DG granular cell layer at PD36

### Total cells

```
#Fit mixed-effects model using dam as random factor
model73<-lme(log(DGgran_total)~Prenatal*Juvenile, data=data_IBA1pd36, na.action=na.exclude, random=~1|Dam)
Anova(model73,type="III")
```

```
## Analysis of Deviance Table (Type III tests)
## 
## Response: log(DGgran_total)
##                      Chisq Df Pr(>Chisq)    
## (Intercept)       207.6594  1  < 2.2e-16 ***
## Prenatal            1.2070  1   0.271922    
## Juvenile            1.1674  1   0.279945    
## Prenatal:Juvenile   6.7469  1   0.009391 ** 
## ---
## Signif. codes:  0 '***' 0.001 '**' 0.01 '*' 0.05 '.' 0.1 ' ' 1
```

```
#Normality and Homocedasticity checks
e73<-resid(model73) # Pearson's residuals
pre73<-predict(model73) #predicted
shapiro.test(e73)
```

```
## 
##  Shapiro-Wilk normality test
## 
## data:  e73
## W = 0.93714, p-value = 0.2587
```

```
leveneTest(DGgran_total~Prenatal*Juvenile, data=data_IBA1pd36)
```

```
## Levene's Test for Homogeneity of Variance (center = median)
##       Df F value Pr(>F)
## group  3  0.8276 0.5004
##       14
```

```
par(mfrow = c(1, 2))
plot(pre73, e73, xlab="Predicted", ylab="Pearson's residuals",main="Dispersion of RE vs PRED",cex.main=.8 )
abline(0,0)
qqnorm(e73, cex.main=.8)
qqline(e73)
```

```
emmeans(model73, pairwise~Prenatal*Juvenile)
```

```
## $emmeans
##  Prenatal Juvenile emmean    SE df lower.CL upper.CL
##  Veh      LPS        2.04 0.141  5     1.67     2.40
##  VPA      LPS        2.27 0.160  4     1.83     2.72
##  Veh      SAL        1.89 0.129  5     1.56     2.23
##  VPA      SAL        1.59 0.180  4     1.09     2.10
## 
## Degrees-of-freedom method: containment 
## Results are given on the log (not the response) scale. 
## Confidence level used: 0.95 
## 
## $contrasts
##  contrast          estimate    SE df t.ratio p.value
##  Veh LPS - VPA LPS   -0.234 0.213  4  -1.099  0.7093
##  Veh LPS - Veh SAL    0.144 0.133 10   1.080  0.7085
##  Veh LPS - VPA SAL    0.443 0.229  4   1.933  0.3435
##  VPA LPS - Veh SAL    0.378 0.205  4   1.841  0.3748
##  VPA LPS - VPA SAL    0.677 0.157 10   4.325  0.0069
##  Veh SAL - VPA SAL    0.299 0.222  4   1.349  0.5840
## 
## Degrees-of-freedom method: containment 
## Results are given on the log (not the response) scale. 
## P value adjustment: tukey method for comparing a family of 4 estimates
```

### Ramified cells

```
#Fit mixed-effects model using dam as random factor
model74<-lme(DGgran_type1~Prenatal*Juvenile, data=data_IBA1pd36, na.action=na.exclude, random=~1|Dam)
Anova(model74,type="III")
```

```
## Analysis of Deviance Table (Type III tests)
## 
## Response: DGgran_type1
##                     Chisq Df Pr(>Chisq)    
## (Intercept)       34.4936  1  4.277e-09 ***
## Prenatal           0.5570  1     0.4555    
## Juvenile           0.3055  1     0.5804    
## Prenatal:Juvenile  2.5887  1     0.1076    
## ---
## Signif. codes:  0 '***' 0.001 '**' 0.01 '*' 0.05 '.' 0.1 ' ' 1
```

```
#Normality and Homocedasticity checks
e74<-resid(model74) # Pearson's residuals
pre74<-predict(model74) #predicted
shapiro.test(e74)
```

```
## 
##  Shapiro-Wilk normality test
## 
## data:  e74
## W = 0.91799, p-value = 0.1191
```

```
leveneTest(DGgran_type1~Prenatal*Juvenile, data=data_IBA1pd36)
```

```
## Levene's Test for Homogeneity of Variance (center = median)
##       Df F value Pr(>F)
## group  3  1.1102  0.378
##       14
```

```
par(mfrow = c(1, 2))
plot(pre74, e74, xlab="Predicted", ylab="Pearson's residuals",main="Dispersion of RE vs PRED",cex.main=.8 )
abline(0,0)
qqnorm(e74, cex.main=.8)
qqline(e74)
```

### Hypertrophic cells

```
#Fit mixed-effects model using dam as random factor
model75<-lme(DGgran_type2~Prenatal*Juvenile, data=data_IBA1pd36, na.action=na.exclude, random=~1|Dam)
Anova(model75,type="III")
```

```
## Analysis of Deviance Table (Type III tests)
## 
## Response: DGgran_type2
##                    Chisq Df Pr(>Chisq)   
## (Intercept)       7.1427  1   0.007527 **
## Prenatal          0.7911  1   0.373767   
## Juvenile          0.4911  1   0.483416   
## Prenatal:Juvenile 0.0385  1   0.844487   
## ---
## Signif. codes:  0 '***' 0.001 '**' 0.01 '*' 0.05 '.' 0.1 ' ' 1
```

```
#Normality and Homocedasticity checks
e75<-resid(model75) # Pearson's residuals
pre75<-predict(model75) #predicted
shapiro.test(e75)
```

```
## 
##  Shapiro-Wilk normality test
## 
## data:  e75
## W = 0.95552, p-value = 0.518
```

```
leveneTest(DGgran_type2~Prenatal*Juvenile, data=data_IBA1pd36)
```

```
## Levene's Test for Homogeneity of Variance (center = median)
##       Df F value Pr(>F)
## group  3  0.3655  0.779
##       14
```

```
par(mfrow = c(1, 2))
plot(pre75, e75, xlab="Predicted", ylab="Pearson's residuals",main="Dispersion of RE vs PRED",cex.main=.8 )
abline(0,0)
qqnorm(e75, cex.main=.8)
qqline(e75)
```

## SUPPLEMENTARY FIGURE 5L: IBA1 in hippocampal DG hilus at PD36

### Total cells

```
#Fit mixed-effects model using dam as random factor
model76<-lme(DGhilus_total~Prenatal*Juvenile, data=data_IBA1pd36, na.action=na.exclude, random=~1|Dam)
Anova(model76,type="III")
```

```
## Analysis of Deviance Table (Type III tests)
## 
## Response: DGhilus_total
##                     Chisq Df Pr(>Chisq)    
## (Intercept)       31.7513  1  1.752e-08 ***
## Prenatal           0.0631  1     0.8017    
## Juvenile           0.8844  1     0.3470    
## Prenatal:Juvenile  0.0131  1     0.9090    
## ---
## Signif. codes:  0 '***' 0.001 '**' 0.01 '*' 0.05 '.' 0.1 ' ' 1
```

```
#Normality and Homocedasticity checks
e76<-resid(model76) # Pearson's residuals
pre76<-predict(model76) #predicted
shapiro.test(e76)
```

```
## 
##  Shapiro-Wilk normality test
## 
## data:  e76
## W = 0.90348, p-value = 0.06609
```

```
leveneTest(DGhilus_total~Prenatal*Juvenile, data=data_IBA1pd36)
```

```
## Levene's Test for Homogeneity of Variance (center = median)
##       Df F value Pr(>F)
## group  3  0.7394 0.5459
##       14
```

```
par(mfrow = c(1, 2))
plot(pre76, e76, xlab="Predicted", ylab="Pearson's residuals",main="Dispersion of RE vs PRED",cex.main=.8 )
abline(0,0)
qqnorm(e76, cex.main=.8)
qqline(e76)
```

### Ramified cells

```
#Fit mixed-effects model using dam as random factor
model77<-lme(DGhilus_type1~Prenatal*Juvenile, data=data_IBA1pd36, na.action=na.exclude, random=~1|Dam)
Anova(model77,type="III")
```

```
## Analysis of Deviance Table (Type III tests)
## 
## Response: DGhilus_type1
##                     Chisq Df Pr(>Chisq)    
## (Intercept)       32.1926  1  1.396e-08 ***
## Prenatal           0.0150  1     0.9026    
## Juvenile           0.6004  1     0.4384    
## Prenatal:Juvenile  0.0582  1     0.8093    
## ---
## Signif. codes:  0 '***' 0.001 '**' 0.01 '*' 0.05 '.' 0.1 ' ' 1
```

```
#Normality and Homocedasticity checks
e77<-resid(model77) # Pearson's residuals
pre77<-predict(model77) #predicted
shapiro.test(e77)
```

```
## 
##  Shapiro-Wilk normality test
## 
## data:  e77
## W = 0.92229, p-value = 0.1419
```

```
leveneTest(DGhilus_type1~Prenatal*Juvenile, data=data_IBA1pd36)
```

```
## Levene's Test for Homogeneity of Variance (center = median)
##       Df F value Pr(>F)
## group  3  0.1081  0.954
##       14
```

```
par(mfrow = c(1, 2))
plot(pre77, e77, xlab="Predicted", ylab="Pearson's residuals",main="Dispersion of RE vs PRED",cex.main=.8 )
abline(0,0)
qqnorm(e77, cex.main=.8)
qqline(e77)
```

### Hypertrophic cells

```
#Fit mixed-effects model using dam as random factor
model78<-lme(DGhilus_type2~Prenatal*Juvenile, data=data_IBA1pd36, na.action=na.exclude, random=~1|Dam)
Anova(model78,type="III")
```

```
## Analysis of Deviance Table (Type III tests)
## 
## Response: DGhilus_type2
##                    Chisq Df Pr(>Chisq)
## (Intercept)       0.7805  1     0.3770
## Prenatal          0.2628  1     0.6082
## Juvenile          0.5948  1     0.4406
## Prenatal:Juvenile 0.1930  1     0.6604
```

```
#Normality and Homocedasticity checks
e78<-resid(model78) # Pearson's residuals
pre78<-predict(model78) #predicted
shapiro.test(e78)
```

```
## 
##  Shapiro-Wilk normality test
## 
## data:  e78
## W = 0.93364, p-value = 0.2249
```

```
leveneTest(DGhilus_type2~Prenatal*Juvenile, data=data_IBA1pd36)
```

```
## Levene's Test for Homogeneity of Variance (center = median)
##       Df F value Pr(>F)
## group  3  0.6269 0.6095
##       14
```

```
par(mfrow = c(1, 2))
plot(pre78, e78, xlab="Predicted", ylab="Pearson's residuals",main="Dispersion of RE vs PRED",cex.main=.8 )
abline(0,0)
qqnorm(e78, cex.main=.8)
qqline(e78)
```

# SUPPLEMENTARY FIGURE 6: EFFECT OF VPA ON LITTERS

```
data_Litters <- read_excel("VPAxLPS_Data.xlsx", sheet = "Litters")
data_Litters[data_Litters==""]<-NA
```

## SUPPLEMENTARY FIGURE 6A: Gestational length

```
# Since the variable has only two values (19 and 20) and in VEH only 3 animals had a 20-day gestation, we performed Fisher's exact test
fisher.test(table(data_Litters$Treatment, data_Litters$Gestational_length))
```

```
## 
##  Fisher's Exact Test for Count Data
## 
## data:  table(data_Litters$Treatment, data_Litters$Gestational_length)
## p-value = 0.1281
## alternative hypothesis: true odds ratio is not equal to 1
## 95 percent confidence interval:
##   0.6218984 27.6097224
## sample estimates:
## odds ratio 
##   3.660149
```

## SUPPLEMENTARY FIGURE 6B: Litter size

```
#Fit linear model using generalized least squares and ANOVA
model1<-gls(Litter_size~Treatment, data=data_Litters, na.action=na.exclude)
anova(model1)
```

```
## Denom. DF: 36 
##             numDF  F-value p-value
## (Intercept)     1 465.3740  <.0001
## Treatment       1   0.7227  0.4009
```

```
#Normality and Homocedasticity checks
e1<-resid(model1) # Pearson's residuals
pre1<-predict(model1) #predicted
shapiro.test(e1)
```

```
## 
##  Shapiro-Wilk normality test
## 
## data:  e1
## W = 0.97689, p-value = 0.6074
```

```
leveneTest(Litter_size~Treatment, data=data_Litters)
```

```
## Warning in leveneTest.default(y = y, group = group, ...): group coerced to
## factor.
```

```
## Levene's Test for Homogeneity of Variance (center = median)
##       Df F value Pr(>F)
## group  1  1.4736 0.2327
##       36
```

```
par(mfrow = c(1, 2))
plot(pre1, e1, xlab="Predicted", ylab="Pearson's residuals",main="Dispersion of RE vs PRED",cex.main=.8 )
abline(0,0)
qqnorm(e1, cex.main=.8)
qqline(e1)
```

## SUPPLEMENTARY FIGURE 6C: Male to female ratio

```
#Fit linear model using generalized least squares and ANOVA
model2<-gls(log(Male_female_ratio)~Treatment, data=data_Litters, na.action=na.exclude)
anova(model2)
```

```
## Denom. DF: 36 
##             numDF   F-value p-value
## (Intercept)     1 0.3190514  0.5757
## Treatment       1 0.0573975  0.8120
```

```
#Normality and Homocedasticity checks
e2<-resid(model2) # Pearson's residuals
pre2<-predict(model2) #predicted
shapiro.test(e2)
```

```
## 
##  Shapiro-Wilk normality test
## 
## data:  e2
## W = 0.92429, p-value = 0.01331
```

```
leveneTest(Male_female_ratio~Treatment, data=data_Litters)
```

```
## Warning in leveneTest.default(y = y, group = group, ...): group coerced to
## factor.
```

```
## Levene's Test for Homogeneity of Variance (center = median)
##       Df F value Pr(>F)
## group  1  0.5143 0.4779
##       36
```

```
par(mfrow = c(1, 2))
plot(pre2, e2, xlab="Predicted", ylab="Pearson's residuals",main="Dispersion of RE vs PRED",cex.main=.8 )
abline(0,0)
qqnorm(e2, cex.main=.8)
qqline(e2)
```
